# Potent Prearranged Positive Allosteric Modulators of the Glucagon‐like Peptide‐1 Receptor

**DOI:** 10.1002/open.201700062

**Published:** 2017-06-05

**Authors:** Ben J. Jones, Rosario Scopelliti, Alejandra Tomas, Stephen R. Bloom, David J. Hodson, Johannes Broichhagen

**Affiliations:** ^1^ Imperial College London, Section of Investigative Medicine Division of Diabetes, Endocrinology and Metabolism London W12 0NN UK; ^2^ École Polytechnique Fédérale de Lausanne ISIC SB, Laboratory of Protein Engineering Av. Forel 2 1015 Lausanne Switzerland; ^3^ Imperial College London Section of Cell Biology and Functional Genomics, Department of Medicine London W12 0NN UK; ^4^ Institute of Metabolism and Systems Research (IMSR) and Centre of Membrane Proteins and Receptors (COMPARE) University of Birmingham Birmingham B15 2TT UK; ^5^ Centre for Endocrinology, Diabetes and Metabolism Birmingham Health Partners Birmingham B15 2TH UK; ^6^ Current address: Max Planck Institute for Medical Research Department of Chemical Biology Jahnstraße 29 69120 Heidelberg Germany

**Keywords:** allosterism, BETP, glucagon-like peptides, glucagon-like peptide-1 receptors, G protein-coupled receptors, stilbene

## Abstract

Drugs that allosterically modulate G protein‐coupled receptor (GPCR) activity display higher specificity and may improve disease treatment. However, the rational design of compounds that target the allosteric site is difficult, as conformations required for receptor activation are poorly understood. Guided by photopharmacology, a set of prearranged positive allosteric modulators (PAMs) with restricted degrees of freedom was designed and tested against the glucagon‐like peptide‐1 receptor (GLP‐1R), a GPCR involved in glucose homeostasis. Compounds incorporating a *trans*‐stilbene comprehensively outperformed those with a *cis*‐stilbene, as well as the benchmark BETP, as GLP‐1R PAMs. We also identified major effects of ligand conformation on GLP‐1R binding kinetics and signal bias. Thus, we describe a photopharmacology‐directed approach for rational drug design, and introduce a new class of stilbene‐containing PAM for the specific regulation of GPCR activity.

G protein‐coupled receptors (GPCRs) are critical for organism homeostasis by converting signals encoded by extracellular molecules into an appropriate cell response.^1^ Classically, ligands bind the extracellularly located orthosteric face of the GPCR, leading to conformational changes and activation of second messenger pathways.^1^ GPCRs are also subject to allosteric regulation, whereby small molecular entities influence orthosteric activation.^2^ Such allosteric modulators are therapeutically desirable, as they demonstrate excellent specificity and selectivity.2a

Recently, we described allosteric optical control of the glucagon‐like peptide‐1 receptor (GLP‐1R),^3^ a prototypical class B GPCR involved in the maintenance of blood glucose levels and a blockbuster target for type 2 diabetes treatment.^4^ As peptide ligands that target the GLP‐1R must be injected, the development of orally available small‐molecule GLP‐1R activators is a priority. In our previous work, an azobenzene‐containing molecular photoswitch termed **PhotoETP** was designed and synthesized,^3^ based upon an “azologue”^5^ of the known GLP‐1R positive allosteric modulator (PAM) 4‐(3‐(benzyloxy)phenyl)‐2‐(ethylsulfinyl)‐6‐(trifluoro‐methyl)pyrimidine (**BETP**).^6^ Notably, signaling responses to glucagon‐like peptide‐1 (GLP‐1) degradation products could be sculpted by the application of blue light to induce formation of the *trans*‐isomer.^3^ We hypothesized that maximal GLP‐1R activation by PAMs requires a molecule with fewer degrees of freedom. As the molecular conformations/interactions required for allosteric activation are largely unknown, we thought that this may be achievable by “prearranging” the molecule.

To produce PAMs with improved activity at the GLP‐1R, the **PhotoETP** azobenzene diazene unit, which exhibits non‐binary photostationary states,^5^ was replaced with a C=C fragment to introduce a stilbene that displays single isomers. A range of prearranged molecules were synthesized and tested for their ability to potentiate GLP‐1R signaling responses. In all cases, compounds incorporating the *trans*‐stilbene outperformed those with *cis*‐stilbene, in addition to native **BETP**. As such, we introduce a photopharmacology‐based strategy to direct the rational design of prearranged PAMs, with broad applicability to the allosteric regulation of GPCRs involved in health and disease.

**BETP** adopts a conformationally free benzyl ether when bound,6b suggesting that, upon activation of the GLP‐1R by orthosteric ligands, it is able to rearrange its shape to fully engage the allosteric site (Figure 1 a). This motion is restricted by light in **PhotoETP**, which possesses a photoisomerizable azobenzene in place of the *O*‐benzyl group (Figure 1 b). We reasoned that replacement of the diazene bridge in **PhotoETP** by a C=C moiety to introduce a stilbene would allow production of prearranged PAMs where the *cis*‐ and *trans*‐states are mimicked, but without complications arising from photostationary states. Stilbenes have the added advantage of being more drug‐like than azobenzenes and may demonstrate better metabolic stability in terms of double‐bond cleavage in the intestine and the possible liberation of reactive anilines. Furthermore, **BETP** uses an ethyl sulfoxide as a leaving group that is replaced by covalent cysteine attachment on the receptor to give ethyl sulfenic acid as a leaving group,6b and other electrophilic moieties, mainly aryl chlorides and aryl sulfones, have also been reported to undergo covalent labelling toward the GLP‐1R.^7^ We, therefore, wanted to explore whether a methyl sulfoxide would be tolerated (Figure 1 c), as this moiety would: 1) be easier and faster to introduce synthetically from commercially‐available substrates, giving cheaper access to PAMs that target the GLP‐1R (Figure 1 d); and 2) exhibit methyl sulfenic acid as an unstable and, therefore, quickly‐cleared leaving group.^8^

**Figure 1:**
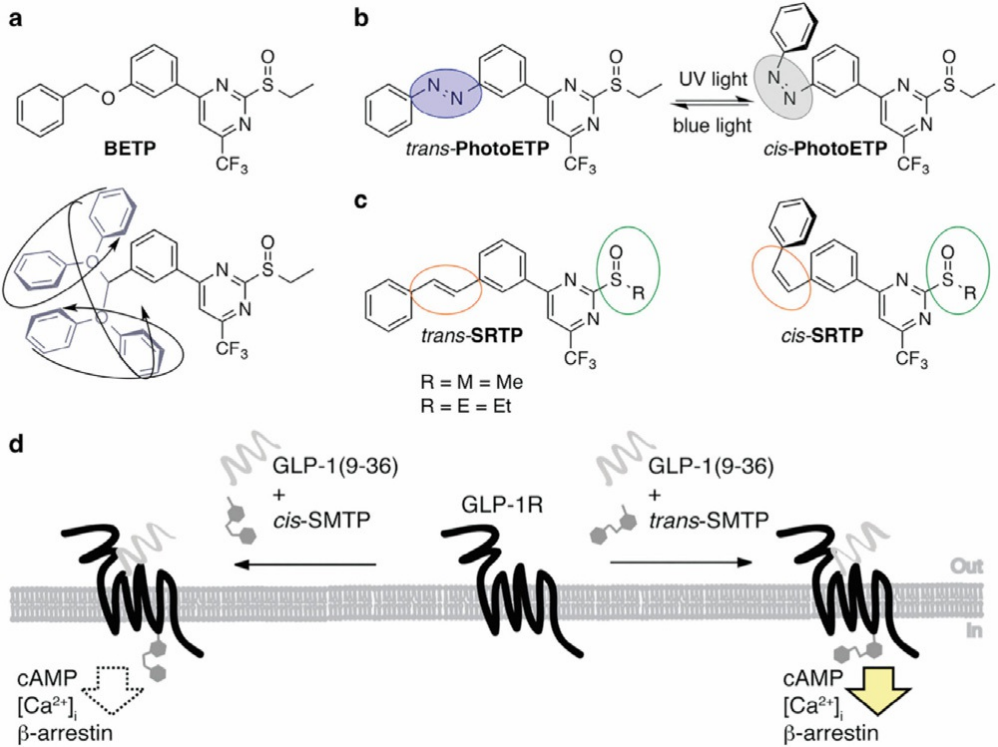
Logic and design of prearranged GLP‐1R positive allosteric modulators. a) **BETP** possesses an *O*‐benzyl ether that can freely rotate and adopt various conformations, occupying a large space, as denoted in the lower drawing. b) **PhotoETP** 3 endows GLP‐1R with light sensitivity when the azobenzene is locked in its active *trans*‐state under blue light illumination (blue circle), which is interchangeable with UV light to its inactive *cis*‐state (gray circle). c) Novel stilbene congeners are constantly locked (orange circle) in their respective state and do not exhibit photostationary states. The functional group for displacement can be a methyl (Me) or ethyl (Et) sulfoxide (green circle), giving pure *trans*‐ and *cis*‐isomers of the prearranged PAMs **SMTP** and **SETP**. d) Schematic representation of GLP‐1R activation with GLP‐1(9–36)NH_2_ in the presence of either *trans*‐ or *cis*‐**SMTP**.

To obtain **BMTP**, that is, the methyl analogue of the lead compound **BETP**, as a first model compound, boronic ester **1** was coupled under Suzuki–Miyaura conditions with chloropyrimidine **2** to biaryl **3** in quantitative yield,^9^ which was monooxidized with one equivalent of *m*CPBA to give **BMTP** in 94 % yield (Scheme 1 a). **SMTP** and **SETP** were synthesized in a comparable synthetic sequence. Commencing with benzyltriphenylphosphonium bromide (**4**) and 3‐bromobenzaldehyde (**5**) that underwent a non‐stereoselective Wittig reaction at room temperature in THF with LiHMDS as base, *cis*‐ and *trans*‐bromo stilbene **6** could be separated through flash column chromatography (FCC) and isolated in 79 % overall yield (Scheme 1 b). Single crystals of *trans*‐**6** were obtained, which unambiguously provided the isomeric identity, and this was further confirmed by the coupling constant ^3^ *J*_HH_ of the olefinic protons through ^1^H NMR spectroscopy, being approximately 12 and 16 Hz for *cis*‐ and *trans*‐isomers, respectively. Next, Miyaura coupling with B_2_pin_2_ using PdCl_2_(dppf) under standard conditions, employing DMSO as the solvent and KOAc as the base,^10^ gave access to *cis*‐ and *trans*‐boronic ester **7** in good yields (78 and 77 %, respectively), which were subsequently subjected to Suzuki–Miyaura Pd cross‐coupling with chloropyrimidine **2** to yield the *cis*‐ and *trans*‐thioether precursor **8** in good 76 % and excellent 95 % yields, respectively. Progressing towards **SMTP**, thioether **6** was monooxidized with one equivalent *m*CPBA to give *cis*‐ and *trans*‐**SMTP** in 91 and 89 %, respectively.

**Scheme 1:**
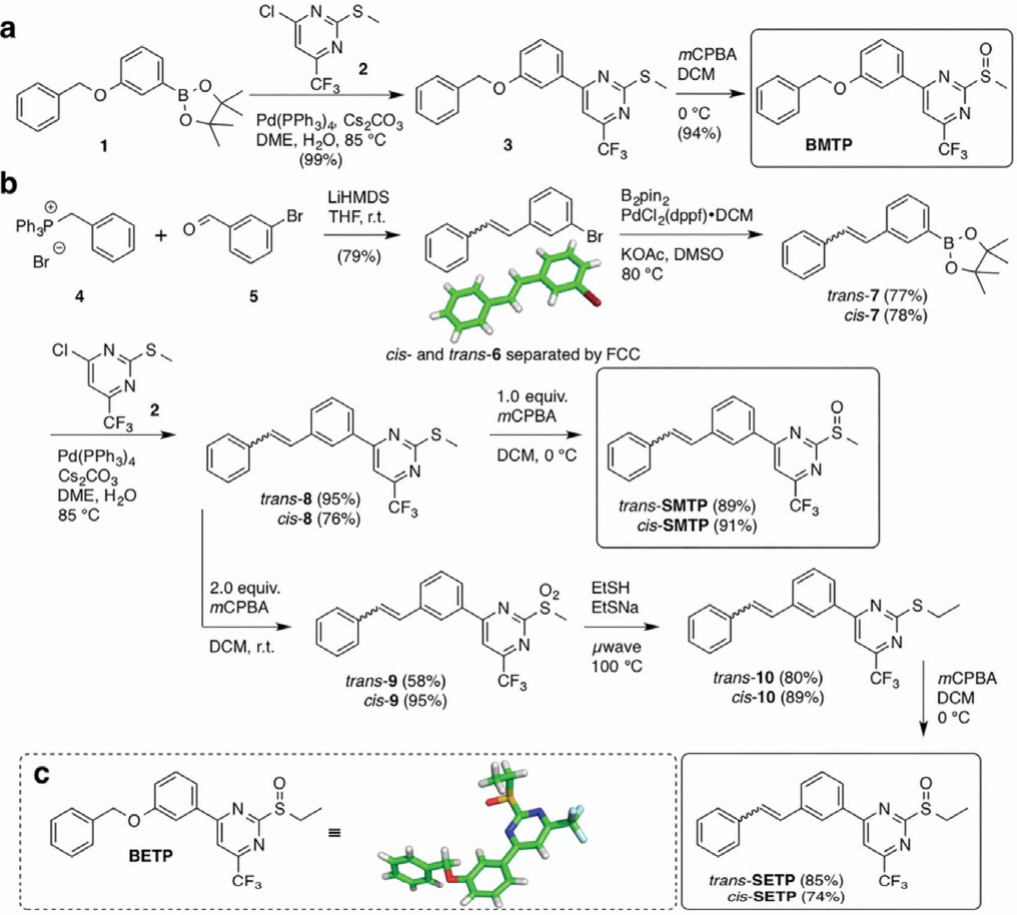
Synthesis of GLP‐1R prearranged PAMs. a) **BMTP** is obtained after a two‐step synthetic sequence from commercially available boronic ester **1** and chloropyrimidine **2** after Pd cross‐coupling and subsequent monooxidation. b) Wittig transformation gives access to *trans*‐ and *cis*‐stilbenes that can be separated by FCC and independently processed towards thioether **8**. *trans*‐ and *cis*‐**SMTP** are obtained as the monooxidized products from **8**, whereas bisoxidation, aromatic substitution, and monooxidation yield the prearranged positive allosteric modulators *trans*‐ and *cis*‐**SETP**. c) Crystal structure of **BETP**.

To progress to the ethyl sulfoxide, thioether **6** was oxidized with two equivalents of *m*CPBA to give the corresponding sulfone **9** (yield for *cis*: 95 %; *trans*: 58 %), which was subjected to aromatic substitution with a mixture of ethylsulfide/sodium thioethanolate to ethyl thioether **10** (*cis*: 89 %; *trans*: 80 %) in a microwave reactor before final installation of the ethyl sulfoxide with one equivalent of *m*CPBA to obtain *cis*‐ and *trans*‐**SETP** in 74 and 85 % yields, respectively. Lastly, we obtained a crystal structure of **BETP** by leaving a DMSO solution open to the atmosphere for 2 weeks (Scheme 1 c), providing atomic coordinates and showing that **BETP** is stable for this timeframe in solution at room temperature and under benchtop light.

The GLP‐1R signals primarily through the generation of cyclic adenosine monophosphate (cAMP).^11^ As expected, **BETP** potently augmented the cAMP responses to the GLP‐1 breakdown product and weak agonist GLP‐1(9–36)NH_2_ (Figure 2 a), but not fully active GLP‐1(7–36)NH_2_ (Figure S1 in the Supporting Information). **BETP** also conferred agonist activity on the GLP‐1R orthosteric antagonist exendin(9–39) (Figure 2 b). Although *cis*‐**SETP** allosterically enhanced cAMP responses, it was outperformed by *trans*‐**SETP**, which displayed a threefold increase in potency as a GLP‐1R PAM (as indicated by *EC_50_* values in Table S1). Similar results were seen with **BMTP** and **SMTP**, suggesting that the replacement of ethyl sulfoxide with its methyl counterpart is well tolerated. Potentiation of GLP‐1(9–36)NH_2_‐induced β‐arrestin2 recruitment to the GLP‐1R was also greatest with *trans*‐isoforms (Figure 2 c).

**Figure 2:**
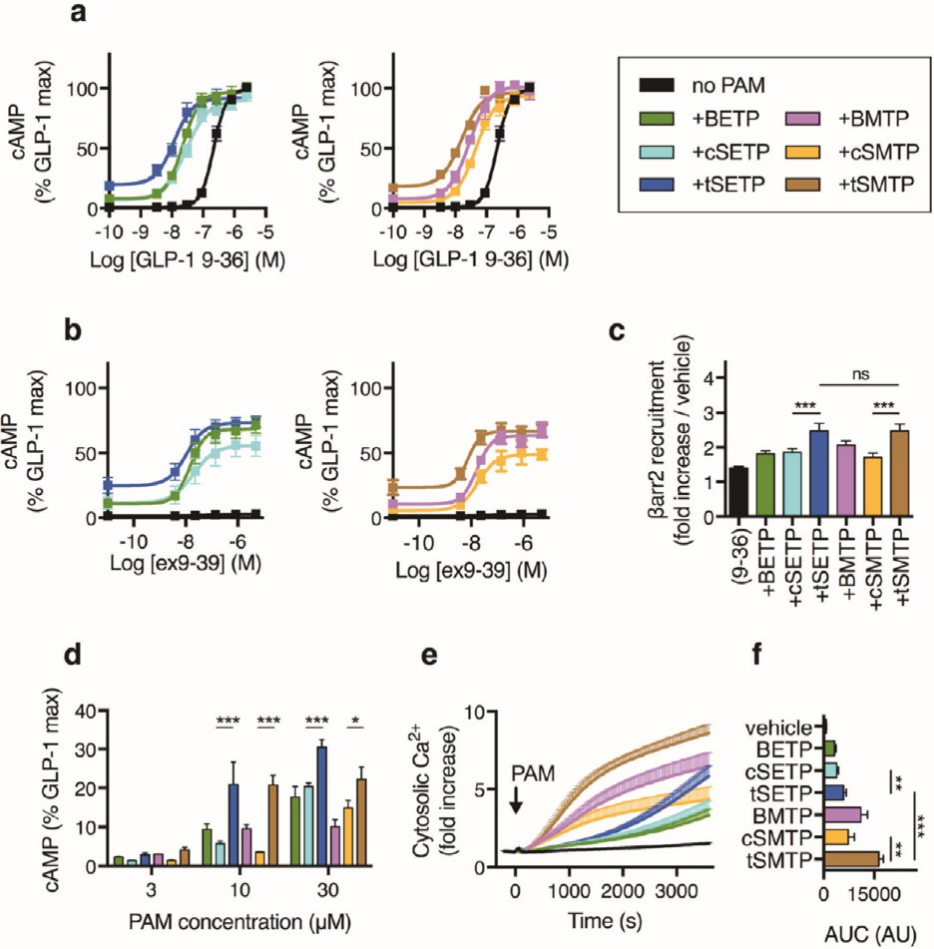
Prearranged PAMs potently enhance GLP‐1R signaling. a) Allosteric enhancement of GLP‐1(9–36)NH_2_ (“9–36”) cAMP responses (30 min incubation; *n=*4) (4‐parameter logistic fit shown). b) As for (a), but with exendin(9–39) (ex9‐39) (*n=*5). c) Allosteric enhancement of 10 μμ GLP‐1(9–36)NH_2_‐induced β‐arrestin2 (βarr2) recruitment (30 min incubation; *n=*5). d) cAMP responses to indicated PAM or prearranged PAM concentration in the absence of orthosteric ligand (30 min incubation; *n=*3). e) Cytosolic Ca^2+^ responses in Calcium 6 dye‐loaded cells, expressed relative to baseline fluorescent signal (60 min incubation; *n=*7). f) Area under curve (AUC) determined from (e). *P<0.05, **P<0.01, ***P<0.001; one‐ or two‐way randomized block ANOVA followed by either Sidak's or Tukey's post‐hoc test. Values represent the mean + or ± SEM. Except where indicated, PAMs or prearranged PAMs were applied at 10 μμ.

PAMs stabilize active receptor conformations and can behave as agonists, even in the absence of the orthosteric ligand. When **BETP**, **BMTP**, and related prearranged PAMs were applied in pure agonist mode, cAMP responses were greatest with *trans*‐diastereomers (Figure 2 d). There was, however, no detectable increase in β‐arrestin2 recruitment with any PAM (Figure S2). We also investigated the ability of these compounds to liberate Ca^2+^ from internal stores. In the absence of GLP‐1, **BMTP** induced strong cytosolic Ca^2+^ rises, more so than **BETP** (Figure 2 e, f). **SETP** and **SMTP** *trans*‐isomers were again most effective. The effects of **BETP** and **BMTP** were likely to be GLP‐1R mediated, as they were almost absent in CHO cells without GLP‐1R overexpression (Figure S3 a) and, like GLP‐1(7–36)NH_2_‐induced responses, could be reduced by inhibiting G‐protein signaling intermediates including Epac2 (ESI09) and phospholipase C (U73122) (Figure S3 b–e). Surprisingly, **BETP** was the only PAM that enhanced the Ca^2+^ response to GLP‐1(7–36)NH_2_ when pre‐incubated, as previously described.2a This may reflect depletion of accessible intracellular Ca^2+^ stores at a rate dependent on the intrinsic activity of the PAM (Figure S4).

PAMs increase receptor affinity for extracellular ligand, coupling to intracellular effectors, or both.^12^ We employed a time‐resolved Förster resonance energy transfer (TR‐FRET) approach to measure ligand‐receptor binding in real‐time, whereby FITC‐conjugated orthosteric ligand binding to SNAP‐GLP‐1R self‐ labeled with Lumi4‐Tb leads to an increase in FRET.^13^ For further details of this technique, see Section 6.6 in the Supporting Information. We determined that GLP‐1R PAMs substantially slow down dissociation of GLP‐1(7–36)NH_2_‐FITC, increasing residence time^14^ (Figure 3 a), and also decreasing the rate of association (Figure 3 b). The overall effect was to increase binding affinity of GLP‐1(7–36)NH_2_‐FITC approximately fivefold (Figure 3 c). *trans*‐**SETP** and *trans*‐**SMTP** increased the residence time and binding affinity more than their *cis*‐counterparts.

**Figure 3:**
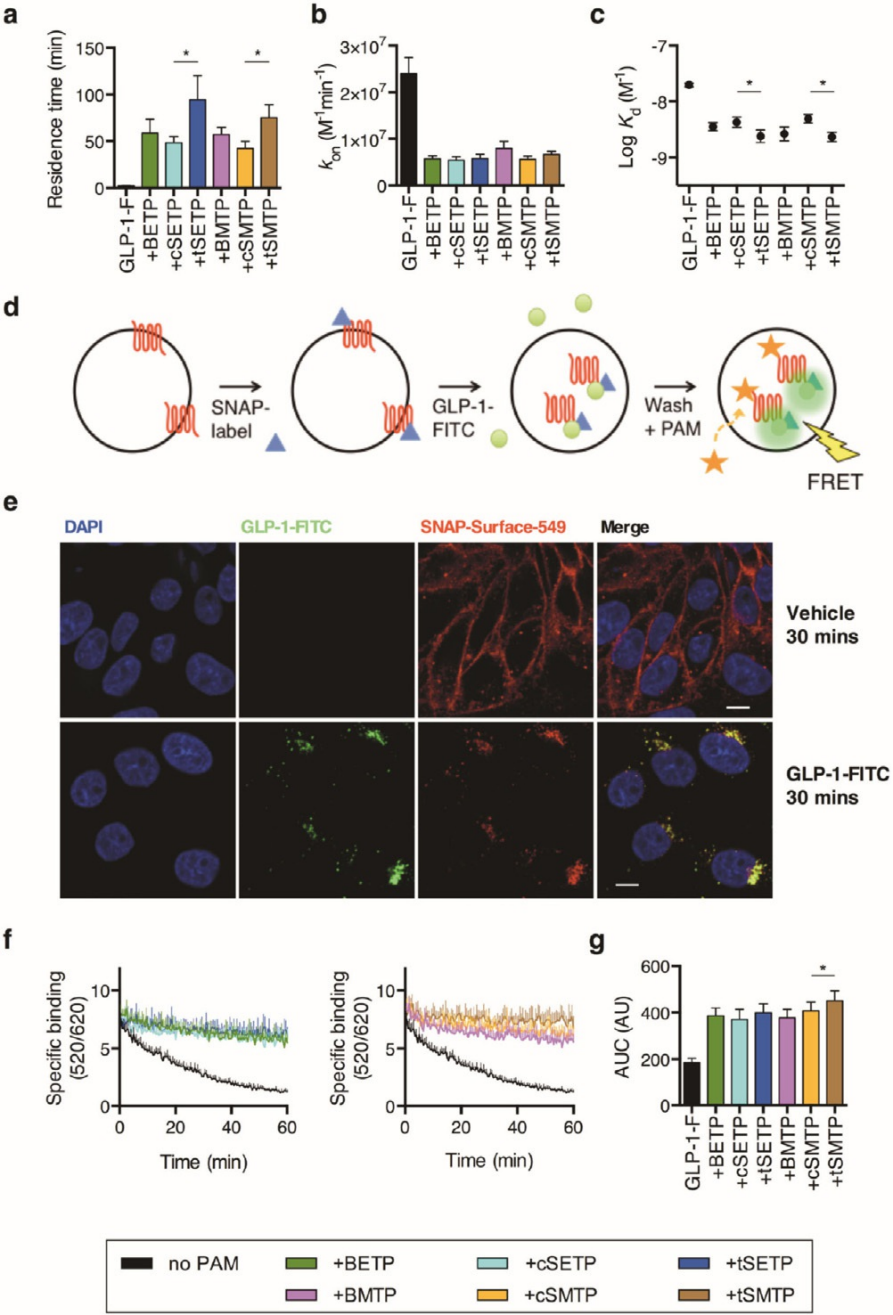
Prearranged PAMs modify GLP‐1 binding kinetics. a) GLP‐1‐FITC residence time (=1/ dissociation rate constant *k*_off_) at surface SNAP‐GLP‐1R after pre‐incubation with PAM or prearranged PAM, determined by non‐linear regression of GLP‐1‐FITC binding data^15^ (*n=*7). b) As for (a), but association rate constant (*k*_on_). c) Effect of PAMs and prearranged PAMs on affinity constants (*K*_d_) for GLP‐1‐FITC (*n=*7). d) Schematic showing the endosomal binding protocol. e) Representative images demonstrating complete internalization of surface‐labeled SNAP‐GLP‐1R by, and co‐localization with, 100 nμ GLP‐1‐FITC (*n=*2) (scale bars; 8 μm). f) Effect of PAMs and prearranged PAMs on endosomal GLP‐1‐FITC dissociation after complete SNAP‐GLP‐1R internalization, agonist washout, and compound application in the presence of exendin(9–39) (*n=*5). g) AUC from (f) relative to zero baseline. *P<0.05; one‐way randomized block ANOVA followed by Sidak's post‐hoc test. Values represent the mean + or ± SEM. PAMs or prearranged PAMs were applied at 10 μμ.

The GLP‐1R undergoes extensive internalization after agonist stimulation, and ongoing cAMP generation by internalized receptors may play a role in GLP‐1R signaling.^16^ We hypothesized that the PAMs described here, which unlike peptide ligands are membrane‐permeating, might access and modulate the behavior of pre‐internalized ligand–receptor complexes (Figure 3 d). After complete GLP‐1R internalization with 100 nμ GLP‐1(7–36)NH_2_‐FITC (Figure 3 e) and washout of remaining extracellular ligand, a steady reduction in FRET was detected, indicative of ligand–receptor dissociation within endosomes. However, when **BETP**, **BMTP**, and prearranged PAMs were applied to the cell immediately post‐washout, marked reductions in dissociation from endosomal receptors were observed (Figure 3 f, g). This effect was most marked with *trans*‐**SMTP**.

In the present study, we describe a photopharmacology‐inspired strategy for the rational design of potent GLP‐1R PAMs based upon the allosteric photoswitch **PhotoETP**.^3^ By restricting degrees of freedom using stilbenes, it could be shown that compounds prearranged as their *trans*‐isomer were more potent than other PAMs, including **BETP**.^6^ Such findings likely reflect the requirement for bound PAMs to undergo fine conformational changes at the allosteric site for full activation in response to orthosteric binding.6b Prearrangement may circumvent this by stabilizing molecule dynamics, allowing PAMs to adopt an allosteric ′ON′ state when unbound. Moreover, replacement of the ethyl sulfoxide moiety with a methyl sulfoxide maintained full activity, whilst promoting cheaper and faster access to PAMs and prearranged PAMs from commercially available chemicals in high yields. Although the differences in signaling described here for the prearranged PAMs may seem small, it should be noted that GLP‐1R PAMs allow less active GLP‐1 metabolites to signal at the orthosteric site,2b and resulting small alterations in cell signaling are expected to translate into large functional differences.

Intriguingly, intracellular Ca^2+^ fluxes induced by **BMTP** exceeded those of **BETP**, despite equivalent cAMP responses. As such, the prearranged PAMs may provide useful tools to tease apart the receptor conformations required for biased signaling and, more widely, the impact of second messenger preference on relevant biological endpoints. Although our studies implicated classical GLP‐1R pathways, the precise mechanism(s) underlying differences in Ca^2+^ responses remain unclear. The different leaving groups for **BMTP** versus **BETP** (methyl versus ethyl sulfenate) are potential candidates, as sulfenic acids are known signaling intermediaries in the context of cysteine modification.^17^ In addition, further studies using recently described conformational FRET sensors,^18^ or concentration responses to calculate alpha values,^12^, ^19^ are required to unambiguously demonstrate the differential actions of the prearranged PAMs at the GLP‐1R. It should be noted that the herein‐described prearranged PAMs, as well as **BETP**, exist as racemates, and in the future it will be interesting to study the labeling kinetics of the separate enantiomers before chirality is lost through covalent modification of GLP‐1R.

Finally, we provide the first demonstration that GLP‐1R PAMs can markedly increase agonist residence time, proposed as a therapeutic strategy to drive sustained responses in vitro and in vivo.^20^ These molecules can also directly access pre‐internalized GLP‐1Rs to modulate ligand–receptor binding within endosomes. They could, therefore, be used to prolong non‐canonical cAMP signaling from internalized GLP‐1Rs,^16^ delineate effects of membrane versus endosomal GLP‐1R signaling, or study the influence of occupancy on post‐endocytic receptor trafficking.^21^

In summary, we unveil a new class of positive allosteric modulator PAMs that are prearranged. These compounds perform better than their compacted stablemates (i.e. those incorporating a *cis*‐stilbene), as well as benchmark PAM, **BETP**, and also display signal bias. Thus, prearranged PAMs provide a template for the production of newer and more potent allosteric modulators of the GLP‐1R, with broad‐applicability to GPCR research and drug discovery.

## Experimental Section

Experimental details including synthesis, spectroscopic and spectrometric characterization, and biology can be found in the Supporting Information. CCDC 1530565 and 1530566 contain the supplementary crystallographic data for this paper. The data can be obtained free of charge from The Cambridge Crystallographic Data Centre via www.ccdc.cam.ac.uk/structures.

## Conflict of interest

*The authors declare no conflict of interest*.

## Supporting information

As a service to our authors and readers, this journal provides supporting information supplied by the authors. Such materials are peer reviewed and may be re‐organized for online delivery, but are not copy‐edited or typeset. Technical support issues arising from supporting information (other than missing files) should be addressed to the authors.

Supplementary

## References

[1] V. Katritch , V. Cherezov , R. C. Stevens , Annu. Rev. Pharmacol. Toxicol. 2013, 53, 531–556. 23140243 10.1146/annurev-pharmtox-032112-135923 PMC3540149

[2a] D. Wootten , E. E. Savage , C. Valant , L. T. May , K. W. Sloop , J. Ficorilli , A. D. Showalter , F. S. Willard , A. Christopoulos , P. M. Sexton , Mol. Pharmacol. 2012, 82, 281–290; 22576254 10.1124/mol.112.079319

[2b] C. Koole , E. E. Savage , A. Christopoulos , L. J. Miller , P. M. Sexton , D. Wootten , Mol. Endocrinol. 2013, 27, 1234–1244. 23864649 10.1210/me.2013-1116 PMC3725346

[3] J. Broichhagen , N. R. Johnston , Y. von Ohlen , H. Meyer-Berg , B. J. Jones , S. R. Bloom , G. A. Rutter , D. Trauner , D. J. Hodson , Angew. Chem. Int. Ed. 2016, 55, 5865–5868; 10.1002/anie.201600957 PMC5031193 27059784

[4] J. E. Campbell , D. J. Drucker , Cell Metab. 2013, 17, 819–837. 23684623 10.1016/j.cmet.2013.04.008

[5] J. Broichhagen , J. A. Frank , D. Trauner , Acc. Chem. Res. 2015, 48, 1947–1960. 26103428 10.1021/acs.accounts.5b00129

[6a] F. S. Willard , D. Wootten , A. D. Showalter , E. E. Savage , J. Ficorilli , T. B. Farb , K. Bokvist , J. Alsina-Fernandez , S. G. Furness , A. Christopoulos , P. M. Sexton , K. W. Sloop , Mol. Pharmacol. 2012, 82, 1066–1073; 22930710 10.1124/mol.112.080432

[6b] W. M. Nolte , J. P. Fortin , B. D. Stevens , G. E. Aspnes , D. A. Griffith , L. R. Hoth , R. B. Ruggeri , A. M. Mathiowetz , C. Limberakis , D. Hepworth , P. A Carpino , Nat. Chem. Biol. 2014, 10, 629–631. 24997604 10.1038/nchembio.1581

[7a] A. B. Bueno , A. D. Showalter , D. B. Wainscott , C. Stutsman , A. Marin , J. Ficorilli , O. Cabrera , F. S. Willard , K. W. Sloop , J. Biol. Chem. 2016, 291, 10700–10715; 26975372 10.1074/jbc.M115.696039 PMC4865917

[7b] L. B. Knudsen , D. Kiel , M. Teng , C. Behrens , D. Bhumralkar , J. T. Kodra , J. J. Holst , C. B. Jeppesen , M. D. Johnson , J. C. de Jong , A. S. Jorgensen , T. Kercher , J. Kostrowicki , P. Madsen , P. H. Olesen , J. S. Petersen , F. Poulsen , U. G. Sidelmann , J. Sturis , L. Truesdale , J. May , J. Lau , Proc. Natl. Acad. Sci. USA 2007, 104, 937–942. 17213325 10.1073/pnas.0605701104 PMC1783418

[8] R. E. Penn , E. Block , L. K. Revelle , J. Am. Chem. Soc. 1978, 100, 3622–3623.

[9] H. Eng , R. Sharma , T. S. McDonald , D. J. Edmonds , J. P. Fortin , X. Li , B. D. Stevens , D. A. Griffith , C. Limberakis , W. M. Nolte , D. A. Price , M. Jackson , A. S. Kalgutkar , Drug Metab. Dispos. 2013, 41, 1470–1479. 23653442 10.1124/dmd.113.052183

[10] T. Ishiyama , M. Murata , N. Miyaura , J. Org. Chem. 1995, 60, 7508–7510.

[11a] C. A. Leech , I. Dzhura , O. G. Chepurny , G. Kang , F. Schwede , H. G. Genieser , G. G. Holz , Prog. Biophys. Mol. Biol. 2011, 107, 236–247; 21782840 10.1016/j.pbiomolbio.2011.07.005 PMC3200499

[11b] P. E. MacDonald , W. El-Kholy , M. J. Riedel , A. M. Salapatek , P. E. Light , M. B. Wheeler , Diabetes 2002, 51, S434–442. 10.2337/diabetes.51.2007.s434 12475787

[12] L. T. May , K. Leach , P. M. Sexton , A. Christopoulos , Annu. Rev. Pharmacol. Toxicol. 2007, 47, 1–51. 17009927 10.1146/annurev.pharmtox.47.120505.105159

[13] A. Emami-Nemini , T. Roux , M. Leblay , E. Bourrier , L. Lamarque , E. Trinquet , M. J. Lohse , Nat. Protoc. 2013, 8, 1307–1320. 23764938 10.1038/nprot.2013.073

[14] C. S. Tautermann , Curr. Opin. Pharmacol. 2016, 30, 22–26. 27428776 10.1016/j.coph.2016.07.004

[15] C. Klein Herenbrink , D. A. Sykes , P. Donthamsetti , M. Canals , T. Coudrat , J. Shonberg , P. J. Scammells , B. Capuano , P. M. Sexton , S. J. Charlton , J. A. Javitch , A. Christopoulos , J. R. Lane , Nat. Commun. 2016, 7, 10842. 26905976 10.1038/ncomms10842 PMC4770093

[16] R. S. Kuna , S. B. Girada , S. Asalla , J. Vallentyne , S. Maddika , J. T. Patterson , D. L. Smiley , R. D. DiMarchi , P. Mitra , Am. J. Physiol. Endocrinol. Metab. 2013, 305, E161–170. 10.1152/ajpendo.00551.2012 23592482

[17] C. E. Paulsen , K. S. Carroll , ACS Chem. Biol. 2010, 5, 47–62. 19957967 10.1021/cb900258z PMC4537063

[18] N. Lecat-Guillet , C. Monnier , X. Rovira , J. Kniazeff , L. Lamarque , J. M. Zwier , E. Trinquet , J. P. Pin , P. Rondard , Cell Chem. Biol 2017, 24, 229–245. 10.1016/j.chembiol.2017.02.011 28286129

[19] P. J. Conn , A. Christopoulos , C. W. Lindsley , Nat. Rev. Drug Discovery 2009, 8, 41–54. 19116626 10.1038/nrd2760 PMC2907734

[20a] D. Guo , L. H. Heitman , I. J. AP , ACS Med. Chem. Lett. 2016, 7, 819–821; 27660682 10.1021/acsmedchemlett.6b00273 PMC5018870

[20b] W. E. de Witte , M. Danhof , P. H. van der Graaf , E. C. de Lange , Trends Pharmacol. Sci. 2016, 37, 831–842. 27394919 10.1016/j.tips.2016.06.008

[21] H. R. Yeatman , J. R. Lane , K. H. Choy , N. A. Lambert , P. M. Sexton , A. Christopoulos , M. Canals , J. Biol. Chem. 2014, 289, 15856–15866. 24753247 10.1074/jbc.M113.536672 PMC4140939

