# Supplementary material for: Potent Prearranged Positive Allosteric Modulators of the Glucagon‐like Peptide‐1 Receptor
Source: ChemistryOpen. 2017 Jun 5;6(4):501–5. doi: 10.1002/open.201700062 (PMC5542757; doi:10.1002/open.201700062)
Supplement: Supplementary file 1 — Supplementary [file OPEN-6-501-s001.pdf]

ChemistryOPEN

Supporting Information

© 2017 The Authors. Published by Wiley-VCH Verlag GmbH & Co. KGaA, Weinheim

Potent Prearranged Positive Allosteric Modulators of the Glucagon-like Peptide-1 Receptor

Ben J. Jones,[a] Rosario Scopelliti,[b] Alejandra Tomas,[c] Stephen R. Bloom,[a] David J. Hodson,*[d, e] and Johannes Broichhagen*[b, f]

open_201700062_sm_miscellaneous_information.pdf

Table of Contents

1. General 5

2. Synthesis 8

2.1. 4-(3-(Benzyloxy)phenyl)-2-(methylthio)-6-(trifluoromethyl)pyrimidine (3) .. 8

2.2. 4-(3-(Benzyloxy)phenyl)-2-(methylsulfinyl)-6-(trifluoromethyl)pyrimidine (BMTP) 9

2.3. 1-Bromo-3-styrylbenzene (6) 10

2.4. 4,4,5,5-Tetramethyl-2-(3-styrylphenyl)-1,3,2-dioxaborolane (7) 12

2.5. 2-(Methylthio)-4-(3-styrylphenyl)-6-(trifluoromethyl)pyrimidine (8) 14

2.6. 2-(Methylsulfinyl)-4-(3-styrylphenyl)-6-(trifluoromethyl)pyrimidine (SMTP) 16

2.7. 2-(Methylsulfonyl)-4-(3-styrylphenyl)-6-(trifluoromethyl)pyrimidine (9) ... 18

2.8. 2-(Ethylthio)-4-(3-styrylphenyl)-6-(trifluoromethyl)pyrimidine (10) 20

2.9. 2-(Ethylsulfinyl)-4-(3-styrylphenyl)-6-(trifluoromethyl)pyrimidine (SETP) 22

3. Spectral data 24

3.1. 4-(3-(Benzyloxy)phenyl)-2-(methylthio)-6-(trifluoromethyl)pyrimidine (3) 24

3.2. 4-(3-(Benzyloxy)phenyl)-2-(methylsulfinyl)-6-(trifluoromethyl)pyrimidine (BMTP) 25

3.3. *cis*-1-Bromo-3-styrylbenzene (*cis*-6) 27

3.4. *trans*-1-Bromo-3-styrylbenzene (*trans*-6) 28

3.5. *cis*-4,4,5,5-Tetramethyl-2-(3-styrylphenyl)-1,3,2-dioxaborolane (*cis*-7) 29

3.6. *trans*-4,4,5,5-Tetramethyl-2-(3-styrylphenyl)-1,3,2-dioxaborolane (*trans*-7) 30

3.7. *cis*-2-(Methylthio)-4-(3-styrylphenyl)-6-(trifluoromethyl)pyrimidine (*cis*-8) 31

3.8. *trans*-2-(Methylthio)-4-(3-styrylphenyl)-6-(trifluoromethyl)pyrimidine (*trans*-8) 32

3.9. *cis*-2-(Methylsulfinyl)-4-(3-styrylphenyl)-6-(trifluoromethyl)pyrimidine (*cis*-SMTP) ... 33
3.10. *trans*-2-(Methylsulfinyl)-4-(3-styrylphenyl)-6-(trifluoromethyl)pyrimidine (*trans*-SMTP) ... 35
3.11. *cis*-2-(Methylsulfonyl)-4-(3-styrylphenyl)-6-(trifluoromethyl)pyrimidine (*cis*-9) 37
3.12. *trans*-2-(Methylsulfonyl)-4-(3-styrylphenyl)-6-(trifluoromethyl)pyrimidine (*trans*-9) ... 38
3.13. *cis*-2-(Ethylthio)-4-(3-styrylphenyl)-6-(trifluoromethyl)pyrimidine (*cis*-10) 39
3.14. *trans*-2-(Ethylthio)-4-(3-styrylphenyl)-6-(trifluoromethyl)pyrimidine (*trans*-10) ... 40
3.15. *cis*-2-(Ethylsulfinyl)-4-(3-styrylphenyl)-6-(trifluoromethyl)pyrimidine (*cis*-SETP) 41
3.16. *trans*-2-(Ethylsulfinyl)-4-(3-styrylphenyl)-6-(trifluoromethyl)pyrimidine (*trans*-SETP) ... 43

4. LCMS traces ... 45
4.1. 4-(3-(Benzyloxy)phenyl)-2-(methylsulfinyl)-6-(trifluoromethyl)pyrimidine 45
4.2. *cis*-2-(Methylsulfinyl)-4-(3-styrylphenyl)-6-(trifluoromethyl)pyrimidine ... 45
4.3. *trans*-2-(Methylsulfinyl)-4-(3-styrylphenyl)-6-(trifluoromethyl)pyrimidine 45
4.4. *cis*-2-(Ethylsulfinyl)-4-(3-styrylphenyl)-6-(trifluoromethyl)pyrimidine ... 46
4.5. *trans*-2-(Ethylsulfinyl)-4-(3-styrylphenyl)-6-(trifluoromethyl)pyrimidine .. 46

5. X-Ray crystallographic tables ... 47
5.1. Crystal data and structure refinement for *trans*-6 ... 47
5.2. Crystal data and structure refinement for BETP. ... 48

6. Biology ... 49
6.1. Peptides ... 49
6.2. Cell culture ... 49
6.3. cAMP assays ... 49
6.4. Ca^{2+} measurements ... 49

6.5. β-Arrestin assays 50
6.6. Cell surface binding kinetics 50
6.7. Endosomal binding studies 51
6.8. Confocal microscopy 51
6.9. Data analysis 52

7. References 59

1. General

All chemical reagents and anhydrous solvents for synthesis were purchased from commercial suppliers (Sigma-Aldrich, Fluka, Acros, Fluorochem, TCI) and were used without further purification or distillation. If necessary, solvents were degassed either by freeze-pump-thaw or by bubbling N_2 through the vigorously stirred solution for several minutes.

NMR spectra were recorded in deuterated solvents on a BRUKER DPX 400 or on a BRUKER Avance III HD 400 (equipped with a CryoProbe™) instruments and calibrated to residual solvent peaks ($^1H/^{13}C$ in ppm): $CDCl_3$ (7.26/77.00). Multiplicities are abbreviated as follows: s = singlet, d = doublet, t = triplet, q = quartet, p = pentet, br = broad, m = multiplet. Coupling constants *J* are reported in Hz and were partially obtained by Global Spectral Deconvolution (GSD) with MestReNova 8.1.2 (Metrelab Research S.L.). Spectra are reported based on appearance, not on theoretical multiplicities derived from structural information.

High-resolution mass spectra (HRMS) were measured on a Xevo G2-S QTOF mass spectrometer coupled to the Acquity UPLC Class Binary Solvent manager and BTN sample manager (Waters, Corporation, Milford, MA). The sample manager system temperature was maintained at 10 °C and the injection volume was 2 µL. Mass spectrometer detection was operated in positive ionization using the ZSpray™ dual-orthogonal multimode ESI/APCI/ESCi® source. The TOF mass spectra were acquired in the sensitive mode over the range of m/z 300-800 at an acquisition rate of 0.036 sec/spectra. The instrument was calibrated using a solution of sodium formate (0.01 mg/L in *i*-propanol/H_2O 90:10). A mass accuracy better than 5 ppm was achieved using a Leucine Enkephalin solution as lock-mass (200 pg/mL in MeCN/H_2O (50:50)) infused continuously using the LockSpray source. Source settings were as follows: cone, 25V; capillary, 3 kV, source temperature, 150 °C; desolvation temperature, 500 °C, cone gas, 10 L/h, desolvation gas, 500 L/h. Data were processed using MassLynx™ 4.1 software and QuanLynx application for quantification. Alternatively, spectra were measured on a LTQ Orbitrap FTMS instrument (LTQ Orbitrap Elite FTMS, Thermo Scientific, Bremen,

Germany) operated in the positive mode coupled with an APPI ionization source with a VUV Kr lamp (Syagen, Tustin, CA, USA) used with the commercial Thermo Scientific Ion Max APCI/APPI source bodies. A standard data acquisition and instrument control system was utilized (Thermo Scientific). Typical nebulizer temperature was fixed at 350 °C whereas the infusion rate was 10 µL/min. The temperature of ion transfer capillary was 275 °C, tube voltages. FTMS spectra were obtained in the 80-1000 m/z range in the reduce profile mode with a resolution set to 120,000. In all spectra one microscan was acquired with a maximum injection time value of 100 ms.

LC-MS was performed on a Shimadzu MS2020 connected to a Nexerra UHPLC system equipped with a Waters ACQUITY UPLC BEH C18 1.7 µm 2.1x50 mm column. Buffer A: 0.05% HCOOH in dH_2O Buffer B: 0.05% HCOOH in MeCN. Analytical gradient was from 10% to 90% B within 6.0 min with 0.5 ml/min flow.

Preparative RP-HPLC was performed on a Dionex system equipped with an UVD 170U UV/Vis detector for product visualization on a Waters SunFire™ Prep C18 OBDTM 5 µm 10×150 mm Column. Buffer A: 0.1% TFA in degassed dH_2O Buffer B: MeCN. Gradient was A/B = 90/10 for 2 min → gradient to 10/90 over 30 min → 10/90 over 8 min with 4 ml/min flow.

Flash column chromatography (FCC) was performed on a Teledyne ISCO CombiFlash® Rf+ with pre-packed silica columns or manually on silica gel (SilicaDlash® P60, 0.040–0.063 mm, 230-400 mesh, Silicycle). Reactions and chromatography fractions were monitored by thin layer chromatography (TLC) on Merck silica gel 60 F254 glass plates. The spots were visualized under UV light at 254 nm.

Microwave synthesis was performed in a CEM Discover SP Synthesizer with the following parameters: P = 150 W, *t* = 10 min, T = 100 °C.

The diffraction data of **BETP** and *trans*-**6** were measured at low temperature [140-100 K] using Cu *K*α and Mo *K*α radiation on an Agilent Technologies SuperNova dual system

in combination with an Atlas CCD detector. The data reduction was carried out by Crysalis PRO.[1] The solutions and refinements were performed by SHELX.[2] The crystal structures were refined using full-matrix least-squares based on F^2 with all non hydrogen atoms anisotropically defined. Hydrogen atoms were placed in calculated positions by means of the “riding” model. Pseudo merohedral twinning was found [TWIN instruction:-1 0 0 0 -1 0 0.18 0 1] for compound **BETP** and refined normally, obtaining a BASF value of: 0.062.[2] Some disorder was found in the S=O moiety of the molecule and the split model - with reasonable restraints and constraints - (SADI, EADP cards) was used to correctly treat it.

2. *Synthesis*

2.1. 4-(3-(Benzyloxy)phenyl)-2-(methylthio)-6-(trifluoromethyl)pyrimidine (3)

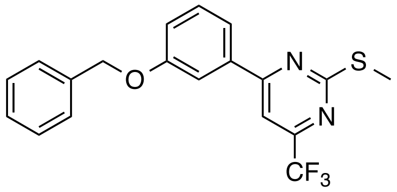

A Schlenk flask was charged under a nitrogen N_2 atmosphere with 137 mg (441 µmol, 1.0 equiv.) of 2-(3-(benzyloxy)phenyl)-4,4,5,5-tetramethyl-1,3,2-dioxaborolane (**1**), 101 mg (441 µmol, 1.0 equiv.) of 4-chloro-2-(methylthio)-6-(trifluoromethyl)pyrimidine (**2**) and 194 mg (595 µmol, 1.35 equiv.) of Cs_2CO_3 and dissolved in a degassed mixture of DME (4 mL) and H_2O (1 mL) before the addition of 101 mg (88.2 µmol, 0.2 equiv.) of $Pd(PPh_3)_4$. The suspension was heated to 85 °C for 18 h to become a clear yellow solution before the volatiles were removed *in vacuo* and the crude material was subjected to FCC (hexanes/DCM = 100/0 over 2 CV → gradient to 0/100 over 10 CV; elution ~60% DCM) to obtain 166 mg (441 µmol) of the desired product as a clear oil in quantitative yield.

^{1}H NMR (400 MHz, $CDCl_3$): δ [ppm] = 7.78 (dd, J = 2.7, 1.6 Hz, 1H), 7.68 (dt, J = 7.8, 1.2 Hz, 1H), 7.61 (s, 1H), 7.51–7.33 (m, 6H), 7.17 (ddd, J = 8.3, 2.6, 1.0 Hz, 1H), 5.17 (s, 2H), 2.66 (s, 3H).

^{13}C NMR (101 MHz, $CDCl_3$): δ [ppm] = 174.3, 165.9, 159.4, 156.3 (q, J = 35.7 Hz), 136.8, 136.5, 130.2, 128.7, 128.2, 127.5, 120.5 (q, J = 275.2 Hz), 120.0, 118.6, 113.75, 107.4 (q, J = 2.8 Hz), 70.3, 14.4.

HRMS (ESI): calc. for $C_{19}H_{16}F_3N_2OS$ $(M+H)^+$: 377.0930, found: 377.0922.

UV/Vis (hexanes/DCM): λ_{max1} = 252 nm, λ_{max2} = 324 nm.

R_f (hexanes/DCM = 1/1): 0.67.

2.2. 4-(3-(Benzyloxy)phenyl)-2-(methylsulfinyl)-6-(trifluoromethyl)pyrimidine (BMTP)

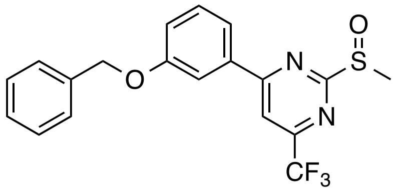

A round bottom flask was charged with 136 mg (370 µmol, 1.0 equiv.) of 2-(methylthio)-4-(3-(benzyloxy)phenyl)-2-(methylthio)-6-(trifluoromethyl)pyrimidine (**3**) dissolved in DCM (5 mL) and cooled to 0 °C before the addition of 83 mg (370 µmol, 1.0 equiv.) of *m*CPBA (77%) dissolved in DCM (5 mL). The reaction was stirred in an ice bath for 90 min before the volatiles were removed *in vacuo* and the crude directly subjected to FCC (EtOAc/DCM = 0/100 over 2 CV → gradient to 0/100 over 10 CV; elution ~50% EtOAc) to obtain 136 mg (346 µmol) of the desired product as a pale-yellow gum in 94% yield.

^{1}H NMR (400 MHz, $CDCl_3$): δ [ppm] = 8.03 (s, 1H), 7.87 (dd, *J* = 2.6, 1.7 Hz, 1H), 7.79 (ddd, *J* = 7.9, 1.8, 0.9 Hz, 1H), 7.51–7.32 (m, 6H), 7.22 (ddd, *J* = 8.4, 2.7, 0.9 Hz, 1H), 5.17 (s, 2H), 3.07 (s, 3H).

^{13}C NMR (101 MHz, $CDCl_3$): δ [ppm] = 174.9, 167.8, 159.2, 157.1 (q, *J* = 37.0 Hz), 135.9, 135.1, 130.2, 128.4, 127.9, 127.3, 120.1, 119.8 (d, *J* = 275.6 Hz), 119.3, 113.8, 112.6 (q, *J* = 2.8 Hz), 70.0, 39.8.

^{19}F NMR (376 MHz, $CDCl_3$): δ [ppm] = –69.5.

HRMS (ESI): calc. for $C_{19}H_{16}F_3N_2O_2S$ $(M+H)^+$: 393.0879, found: 393.0882.

UV/Vis (DCM/EtOAc): λ_{max1} = 228 nm, λ_{max2} = 293 nm.

R_f (DCM/EtOAc = 1/2): 0.48.

t_R (LCMS) = 4.364 min.

LRMS (ESI, LCMS**)**: calc. for $C_{19}H_{16}F_3N_2O_2S$ $(M+H)^+$: 393.1, found: 393.0.

2.3. 1-Bromo-3-styrylbenzene (6)

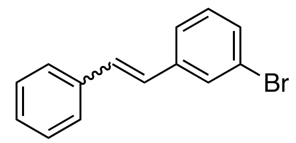

A round bottom flask was charged with benzyltriphenylphosphonium bromide (**4**) (2.00 g, 4.62 mmol, 1.0 equiv.) as a suspension in dry THF (15 mL) under a nitrogen atmosphere. LiHMDS (1.0 M in THF) (4.62 mmol, 4.62 mL, 1.0 equiv.) was added drop-wise, the solution turned orange and was stirred for additional 10 min at r.t.. Then, 3-bromobenzaldehyde (**5**) (854 mg, 4.62 mmol, 1.0 equiv) was added and the reaction mixture was stirred for 1.5 h before it was diluted with 50 mL Et_2O and the precipitate was filtered off. The crude was washed with dH_2O and then subjected to FCC (100% hexanes) to separate and afford 947 mg (3.65 mmol) of *cis*- and *trans*-1-bromo-3-styrylbenzene in 79% overall yield.

cis-1-Bromo-3-styrylbenzene (colorless liquid)

^{1}H NMR (400 MHz, $CDCl_3$): δ [ppm] = 7.40 (t, J = 1.8 Hz, 1H), 7.33 (dt, J = 7.9, 1.5 Hz, 1H), 7.29–7.19 (m, 5H), 7.16 (dt, J = 7.8, 1.3 Hz, 1H), 7.08 (t, J = 7.8 Hz, 1H), 6.66 (d, J = 12.2 Hz, 1H), 6.52 (d, J = 12.2 Hz, 1H).

^{13}C NMR (101 MHz, $CDCl_3$): δ [ppm] = 139.4, 136.6, 131.7, 131.6, 130.0, 129.7, 128.8, 128.6, 128.3, 127.4, 127.4, 122.2.

HRMS (APPI): calc. for $C_{14}H_{11}Br$ $(M)^{\bullet+}$: 258.0039 and 260.0018, found: 258.0040 and 260.0019.

R_f (hexanes): 0.58.

trans-1-Bromo-3-styrylbenzene (white crystalline solid)

Crystals suitable for X-ray crystallography were obtained by allowing a solution of *trans*-**6** in DCM to evaporate under benchtop conditions open to the atmosphere.

^{1}**H NMR** (400 MHz, $CDCl_3$): δ [ppm] = 7.61 (t, J = 1.8 Hz, 1H), 7.49–7.42 (m, 2H), 7.36–7.31 (m, 4H), 7.29–7.20 (m, 1H), 7.16 (t, J = 7.9 Hz, 1H), 7.04 (d, J = 16.3 Hz, 1H), 6.95 (d, J = 16.3 Hz, 1H).

^{13}**C NMR** (101 MHz, $CDCl_3$): δ [ppm] = 139.5, 136.7, 130.3, 130.1 (2C according to HSQC), 129.2, 128.7, 128.0, 127.0, 126.6, 125.1, 122.8.

HRMS (APPI): calc. for $C_{14}H_{11}Br$ $(M)^{\bullet +}$: 258.0039 and 260.0018, found: 258.0040 and 260.0019.

$\boldsymbol{R_f}$ (hexanes): 0.48.

2.4. 4,4,5,5-Tetramethyl-2-(3-styrylphenyl)-1,3,2-dioxaborolane (7)

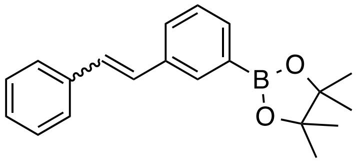

Miyaura-coupling was performed according to the protocol described by Ishiyama *et al.*.[3]

A Schlenk flask was charged with 1-bromo-3-styrylbenzene (**6**) (179 mg, 691 µmol, 1.0 equiv.) and KOAc (203 mg, 2.07 mmol, 3.0 equiv.), purged with N_2, and DMSO (4 mL) was added. To the solution was added under a nitrogen atmosphere $PdCl_2$(dppf)•CH_2Cl_2 (17 mg, 20.7 µmol, 0.03 equiv.) and bis(dipinacolato)diboron (193 mg, 760 µmol, 1.1 equiv.) and the reaction mixture was heated to 85 °C for 2 hours (the red solution turned black), cooled to r.t., diluted with DCM (100 mL), washed with water (2 x 50 mL) and brine (50 mL) and dried over $MgSO_4$ before the volatiles were removed *in vacuo* and the crude material subjected to FCC (hexanes/DCM = 100/0 over 2 CV → gradient to 0/100 over 10 CV; elution ~60% DCM) to obtain 165 and 164 mg (539 µmol and 536 µmol) of the desired product as a colorless oil and a white solid in 78% and 77% yield for *cis* and *trans*, respectively.

cis-4,4,5,5-Tetramethyl-2-(3-styrylphenyl)-1,3,2-dioxaborolane

^{1}H NMR (400 MHz, $CDCl_3$): δ [ppm] = 7.72 (d, J = 2.0 Hz, 1H), 7.64 (dd, J = 7.4, 1.6 Hz, 1H), 7.33 (dd, J = 7.9, 1.6 Hz, 1H), 7.25–7.14 (m, 6H), 6.63 (d, J = 12.3 Hz, 1H), 6.59 (d, J = 12.2 Hz, 1H), 1.34 (s, 12H).

^{13}C NMR (101 MHz, $CDCl_3$): δ [ppm] = 137.2, 136.6, 135.5, 133.4, 131.4, 130.2, 130.2, 128.9, 128.1, 127.4, 127.0, 83.8, 24.9 (one C missing presumably due to the quadrupole moment of ^{11}B).

HRMS (APPI): calc. for $C_{20}H_{23}O_2B$ (M)$^{\bullet+}$: 306.1786, found: 306.1790.

UV/Vis (hexanes/DCM): λ_{max1} = 233 nm, λ_{max2} = 284 nm.

***R*$_f$** (hexanes/DCM = 3/1): 0.35.

trans-4,4,5,5-Tetramethyl-2-(3-styrylphenyl)-1,3,2-dioxaborolane

^{1}H NMR (400 MHz, $CDCl_3$): *δ* [ppm] = 8.03–7.99 (m, 1H), 7.74 (dt, *J* = 7.4, 1.2 Hz, 1H), 7.63 (dt, *J* = 7.9, 1.6 Hz, 1H), 7.56–7.51 (m, 2H), 7.41 (d, *J* = 7.4 Hz, 1H), 7.37 (d, *J* = 7.6 Hz, 2H), 7.31–7.25 (m, 1H), 7.20 (d, *J* = 16.4 Hz, 1H), 7.15 (d, *J* = 16.4 Hz, 1H), 1.39 (s, 12H).

^{13}C NMR (101 MHz, $CDCl_3$): *δ* [ppm] = 137.4, 136.6, 134.0, 132.8, 129.3, 128.7, 128.6, 128.5, 128.1, 127.5, 126.4, 83.9, 24.9 (one C missing presumably due to the quadrupole moment of ^{11}B).

HRMS (APPI): calc. for $C_{20}H_{23}O_2B$ (M)$\bullet^{+}$: 306.1786, found: 306.1790.

UV/Vis (hexanes/DCM): λ_{max1} = 226 nm, λ_{max2} = 304 nm.

R_f (hexanes/DCM = 3/1): 0.33.

2.5. 2-(Methylthio)-4-(3-styrylphenyl)-6-(trifluoromethyl)pyrimidine (8)

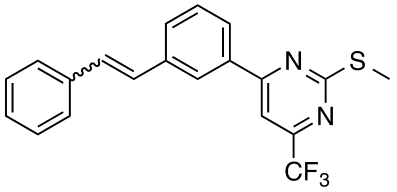

cis-2-(Methylthio)-4-(3-styrylphenyl)-6-(trifluoromethyl)pyrimidine

A Schlenk flask was charged under a nitrogen N_2 atmosphere with 101 mg (441 μmol, 1.0 equiv.) of 4-chloro-2-(methylthio)-6-(trifluoromethyl)pyrimidine (**2**), 135 mg (441 μmol, 1.0 equiv.) of 4,4,5,5-tetramethyl-2-(3-styrylphenyl)-1,3,2-dioxaborolane (*cis*-**7**) and 194 mg (595 μmol, 1.35 equiv.) of Cs_2CO_3 and dissolved in a degassed mixture of DME (4 mL) and H_2O (1 mL) before the addition of 102 mg (88.2 μmol, 0.2 equiv.) of $Pd(PPh_3)_4$. The suspension was heated to 85 °C for 18 h to become a clear yellow solution before the volatiles were removed *in vacuo* and the crude material was subjected to FCC (hexanes/DCM = 100/0 over 1 CV → gradient to 0/100 over 10 CV; elution ~55% DCM) to obtain 125 mg (336 μmol) of the desired product as a white wax in 76% yield.

^{1}H NMR (400 MHz, $CDCl_3$): *δ* [ppm] = 7.82 (dt, *J* = 7.4, 1.8 Hz, 1H), 7.78 (t, *J* = 1.7 Hz, 1H), 7.30–7.21 (m, 2H), 7.18 (s, 1H), 7.15–7.10 (m, 5H), 6.61 (d, *J* = 12.2 Hz, 1H), 6.51 (d, *J* = 12.2 Hz, 1H), 2.45 (s, 3H).

^{13}C NMR (101 MHz, $CDCl_3$): *δ* [ppm] = 173.9, 165.7, 155.9 (q, *J* = 36.1 Hz), 137.6, 136.7, 134.9, 132.3, 131.3, 128.9, 128.7, 128.4, 128.1, 127.6, 127.2, 125.8, 120.2 (q, *J* = 275.1 Hz), 107.0 (q, *J* = 2.8 Hz), 14.0.

HRMS (ESI): calc. for $C_{20}H_{16}F_3N_2S$ $(M+H)^+$: 373.0981, found: 373.0983.

UV/Vis (hexanes/DCM): λ_{max} = 262 nm.

R_f (hexanes/DCM = 3/1): 0.50.

trans-2-(Methylthio)-4-(3-styrylphenyl)-6-(trifluoromethyl)pyrimidine

A Schlenk flask was charged under a nitrogen N_2 atmosphere with 71 mg (310 µmol, 1.0 equiv.) of 4-chloro-2-(methylthio)-6-(trifluoromethyl)pyrimidine (**2**), 95 mg (310 µmol, 1.0 equiv.) of 4,4,5,5-tetramethyl-2-(3-styrylphenyl)-1,3,2-dioxaborolane (*trans*-**7**) and 136 mg (419 µmol, 1.35 equiv.) of Cs_2CO_3 and dissolved in a degassed mixture of DME (4 mL) and H_2O (1 mL) before the addition of 72 mg (88.2 µmol, 0.2 equiv.) of $Pd(PPh_3)_4$. The suspension was heated to 85 °C for 18 h to become a clear yellow solution before the volatiles were removed *in vacuo* and the crude material was subjected to FCC (hexanes/DCM = 100/0 over 1 CV → gradient to 0/100 over 10 CV; elution ~65% DCM) to obtain 109 mg (239 µmol) of the desired product as a white wax in 95% yield.

^{1}H NMR (400 MHz, $CDCl_3$): δ [ppm] = 8.08 (t, J = 1.8 Hz, 1H), 7.87–7.82 (m, 1H), 7.57 (dt, J = 7.8, 1.4 Hz, 1H), 7.53 (s, 1H), 7.45–7.40 (m, 2H), 7.37 (t, J = 7.7 Hz, 1H), 7.12–7.03 (m, 2H), 7.30–7.24 (m, 2H), 7.22–7.15 (m, 1H), 7.08 (d, J = 16.4 Hz, 1H), 7.03 (d, J = 16.3 Hz, 1H), 2.57 (s, 3H).

^{13}C NMR (101 MHz, $CDCl_3$): δ [ppm] = 174.0, 165.7, 155.9 (q, J = 35.8 Hz), 137.9, 136.5, 135.4, 129.7, 129.3, 129.0, 128.4, 127.7, 127.2, 126.3, 126.2, 125.2, 120.2 (q, J = 275.5 Hz), 107.0 (q, J = 2.9 Hz), 14.0.

HRMS (ESI): calc. for $C_{20}H_{16}F_3N_2S$ $(M+H)^+$: 373.0981, found: 373.0990.

UV/Vis (hexanes/DCM): λ_{max} = 270 nm.

R$_f$ (hexanes/DCM = 3/1): 0.43.

2.6. 2-(Methylsulfinyl)-4-(3-styrylphenyl)-6-(trifluoromethyl)pyrimidine (SMTP)

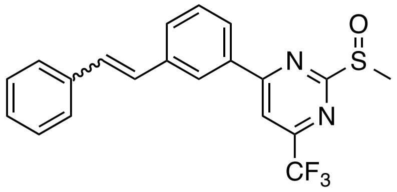

cis-2-(Methylsulfinyl)-4-(3-styrylphenyl)-6-(trifluoromethyl)pyrimidine

A round bottom flask was charged with 30.0 mg (80.6 µmol, 1.0 equiv.) of 2-(methylthio)-4-(3-styrylphenyl)-6-(trifluoromethyl)pyrimidine dissolved in DCM (5 mL) and cooled to 0 °C before the addition of 18.1 mg (80.6 µmol, 1.0 equiv.) of *m*CPBA (77%) as a slurry in DCM (2 mL). The reaction was stirred in an ice bath for 90 min before the volatiles were removed *in vacuo* and the crude directly subjected to FCC (EtOAc/DCM = 0/100 over 5 CV → gradient to 0/100 over 4 CV → EtOAc/DCM = 100/0 over 5 CV; elution ~90% EtOAc) to obtain 28.4 mg (73.1 µmol) of the desired product as a yellow wax in 91% yield.

^{1}H NMR (400 MHz, $CDCl_3$): δ [ppm] = 8.07 (dt, J = 7.7, 1.6 Hz, 1H), 7.95 (t, J = 1.7 Hz, 1H), 7.67 (s, 1H), 7.50–7.35 (m, 2H), 7.27–7.20 (m, 5H), 6.77 (d, J = 12.1 Hz, 1H), 6.64 (d, J = 12.1 Hz, 1H), 3.01 (s, 3H).

^{13}C NMR (101 MHz, $CDCl_3$): δ [ppm] = 175.0, 168.2, 157.3 (q, J = 37.0 Hz), 138.2, 137.0, 133.8 (d, J = 5.3 Hz), 132.0, 129.4, 128.9, 128.7, 128.5, 128.2, 127.5, 126.6, 120.1 (q, J = 275.8 Hz), 112.7 (q, J = 2.7 Hz), 40.0.

^{19}F NMR (376 MHz, $CDCl_3$): δ [ppm] = –69.5.

HRMS (ESI): calc. for $C_{20}H_{16}F_3N_2OS$ $(M+H)^+$: 389.0930, found: 389.0935.

UV/Vis (EtOAc): λ_{max1} = 230 nm, λ_{max2} = 290 nm.

R_f (DCM): 0.16.

t_R (LCMS) = 4.190 min.

LRMS (ESI, LCMS**)**: calc. for $C_{20}H_{16}F_3N_2OS$ $(M+H)^+$: 389.1, found: 389.0.

trans-2-(Methylsulfinyl)-4-(3-styrylphenyl)-6-(trifluoromethyl)pyrimidine

A round bottom flask was charged with 30.0 mg (80.6 µmol, 1.0 equiv.) of 2-(methylthio)-4-(3-styrylphenyl)-6-(trifluoromethyl)pyrimidine dissolved in DCM (5 mL) and cooled to 0 °C before the addition of 18.1 mg (80.6 µmol, 1.0 equiv.) of *m*CPBA (77%) as a slurry in DCM (2 mL). The reaction was stirred in an ice bath for 90 min before the volatiles were removed *in vacuo* and the crude directly subjected to FCC (EtOAc/DCM = 0/100 over 5 CV → gradient to 0/100 over 20 CV; elution ~65% EtOAc) to obtain 27.8 mg (71.6 µmol) of the desired product as yellow wax in 89% yield.

^{1}H NMR (400 MHz, $CDCl_3$): δ [ppm] = 8.34 (t, J = 1.8 Hz, 1H), 8.12–8.06 (m, 2H), 7.77 (dt, J = 8.0, 1.3 Hz, 1H), 7.58–7.51 (m, 3H), 7.38 (dd, J = 8.5, 6.8 Hz, 2H), 7.34–7.27 (m, 1H), 7.23 (d, J = 16.3 Hz, 1H), 7.17 (d, J = 16.4 Hz, 1H), 3.10 (s, 3H).

^{13}C NMR (101 MHz, $CDCl_3$): δ [ppm] = 174.9, 168.0, 157.1 (q, J = 37.0 Hz), 138.4, 136.3, 134.2, 130.3, 130.2, 129.4, 128.4, 127.8, 126.8, 126.5, 126.3, 125.7, 119.8 (q, J = 275.7 Hz), 112.5 (q, J = 2.7 Hz), 39.8.

^{19}F NMR (376 MHz, $CDCl_3$): δ [ppm] = –69.4.

HRMS (ESI): calc. for $C_{20}H_{15}F_3N_2NaOS$ $(M+Na)^+$: 411.0749, found: 411.0752.

UV/Vis (EtOAc): λ_{max1} = 230 nm, λ_{max2} = 295 nm.

R_f (DCM): 0.12.

t_R (LCMS) = 4.167 min.

LRMS (ESI, LCMS**)**: calc. for $C_{20}H_{16}F_3N_2OS$ $(M+H)^+$: 389.1, found: 389.0.

2.7. 2-(Methylsulfonyl)-4-(3-styrylphenyl)-6-(trifluoromethyl)pyrimidine (9)

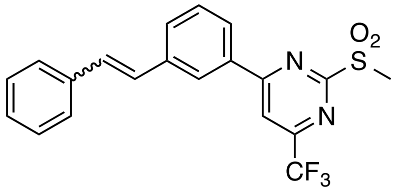

cis-2-(Methylsulfonyl)-4-(3-styrylphenyl)-6-(trifluoromethyl)pyrimidine

A round bottom flask was charged with 95.0 mg (255 µmol, 1.0 equiv.) of 2-(methylthio)-4-(3-styrylphenyl)-6-(trifluoromethyl)pyrimidine (*cis*-**8**) dissolved in DCM (10 mL) and cooled to 0 °C before the addition of 114 mg (511 µmol, 2.0 equiv.) of *m*CPBA (77%) as a slurry in DCM (5 mL). The reaction was stirred in an ice bath for 2 h before it was allowed to warm to r.t. o.n.. The crude was directly subjected to FCC (EtOAc/DCM = 0/100 over 3 CV → gradient to 0/100 over 10 CV; elution ~5% EtOAc) to obtain 98.2 mg (243 µmol) of the desired product as yellow oil in 95% yield.

^{1}H NMR (400 MHz, $CDCl_3$): δ [ppm] = 8.06 (dt, J = 7.8, 1.5 Hz, 1H), 7.97–7.93 (m, 1H), 7.79 (s, 1H), 7.51–7.39 (m, 2H), 7.27 –7.22 (m, 5H), 6.78 (d, J = 12.1 Hz, 1H), 6.64 (d, J = 12.1 Hz, 1H), 3.37 (s, 3H).

^{13}C NMR (101 MHz, $CDCl_3$): δ [ppm] = 168.8, 166.8, 157.3 (q, J = 37.4 Hz), 138.4, 136.9, 134.2, 133.3, 132.2, 129.6, 128.7, 128.7, 128.5, 128.2, 127.6, 126.6, 119.9 (q, J = 275.9 Hz), 114.6 (q, J = 2.7 Hz), 39.0.

HRMS (ESI): calc. for $C_{20}H_{15}F_3N_2NaO_2S$ $(M+Na)^+$: 427.0699, found: 427.0698.

UV/Vis (EtOAc/DCM): λ_{max} = 230 nm, λ_{max2} = 290 nm.

R$_f$ (hexanes/DCM = 1/4): 0.33.

trans-2-(Methylsulfonyl)-4-(3-styrylphenyl)-6-(trifluoromethyl)pyrimidine

A round bottom flask was charged with 79.0 mg (212 µmol, 1.0 equiv.) of 2-(methylthio)-4-(3-styrylphenyl)-6-(trifluoromethyl)pyrimidine (*trans*-**8**) dissolved in DCM (10 mL) and cooled to 0 °C before the addition of 95 mg (415 µmol, 2.0 equiv.) of *m*CPBA (77%) as a slurry in DCM (5 mL). The reaction was stirred in an ice bath for 2 h before it was allowed to warm to r.t. o.n.. The crude was directly subjected to FCC (EtOAc/DCM = 0/100 over 3 CV → gradient to 0/100 over 10 CV; elution ~5% EtOAc) to obtain 50.0 mg (124 µmol) of the desired product as yellow solid in 58% yield.

^{1}H NMR (400 MHz, $CDCl_3$): δ [ppm] = 8.31–8.28 (m, 1H), 8.21 (s, 1H), 8.09–8.04 (m, 1H), 7.78 (dt, J = 7.9, 1.3 Hz, 1H), 7.56–7.51 (m, 2H), 7.41–7.35 (m, 3H), 7.33–7.28 (m, 1H), 7.21 (d, J = 16.4 Hz, 1H), 7.15 (d, J = 16.4 Hz, 1H), 3.49 (s, 3H).

^{13}C NMR (101 MHz, $CDCl_3$): δ [ppm] = 169.0, 166.9, 157.3 (q, J = 37.3 Hz), 138.9, 136.6, 134.0, 131.0, 130.7, 129.9, 128.8, 128.3, 127.1, 126.9, 126.8, 126.2, 120.0 (q, J = 275.8 Hz), 114.8 (q, J = 2.8 Hz), 39.2.

HRMS (ESI): calc. for $C_{20}H_{15}F_3N_2NaO_2S$ $(M+Na)^+$: 427.0699, found: 427.0702.

UV/Vis (EtOAc/DCM): λ_{max1} = 227 nm, λ_{max2} = 293 nm.

R_f (hexanes/DCM = 1/4): 0.28.

2.8. 2-(Ethylthio)-4-(3-styrylphenyl)-6-(trifluoromethyl)pyrimidine (10)

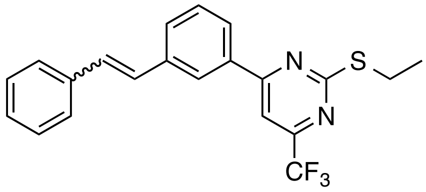

cis-2-(Ethylthio)-4-(3-styrylphenyl)-6-(trifluoromethyl)pyrimidine

A microwave vial was charged with 12.8 mg (31.7 µmol, 1.0 equiv.) of *cis*-2-(methylsulfonyl)-4-(3-styrylphenyl)-6-(trifluoromethyl)pyrimidine (*cis*-**9**) dissolved in a mixture of dry THF (3 mL) and EtSH (50 µL) before the addition of 2.7 mg (31.7 µmol, 1.0 equiv.) of EtSNa*. The reaction was heated in a microwave for 10 min at 100 °C (P = 150 W), cooled to r.t. and the volatiles removed *in vacuo* before the crude material was directly subjected to FCC (hexanes/DCM = 100/0 over 2 CV → gradient to 0/100 over 10 CV; elution ~70% DCM) to obtain 9.8 mg (25.4 µmol) of the desired product in 80% yield as a clear oil. Due to convincing characterization by ^{1}H NMR (sulfonyl methyl group replaced by ethyl thioether signature), HRMS and R_f shift, no ^{13}C was obtained.

^{1}H NMR (400 MHz, $CDCl_3$): δ [ppm] = 7.91 (dt, *J* = 7.3, 1.8 Hz, 1H), 7.86 (t, *J* = 1.8 Hz, 1H), 7.40–7.30 (m, 2H), 7.25–7.17 (m, 6H), 6.70 (d, *J* = 12.2 Hz, 1H), 6.60 (d, *J* = 12.1 Hz, 1H), 3.13 (q, *J* = 7.3 Hz, 2H), 1.36 (t, *J* = 7.3 Hz, 3H).

^{19}F NMR (376 MHz, $CDCl_3$): δ [ppm] = –70.2.

HRMS (ESI): calc. for $C_{21}H_{18}F_3N_2S$ $(M+H)^+$: 387.1137, found: 387.1143.

UV/Vis (hexanes/DCM): λ_{max} = 262 nm.

R_f (hexanes/DCM = 2/1): 0.59.

---

* EtSNa was freshly prepared by the following procedure: An oven-dried Schlenk flask was charged with a stirring bar and 200 mg of NaH (60% in mineral oil) and washed under a N_2 stream with hexanes (2x) by using a Pasteur pipette and carefully removing the solvent without taking up any solids. CAUTION: Dry NaH ist highly flammable; keep under inert gas all the time. After drying under a N_2 stream, 360 µL of EtSH was added dropwise under vigorous stirring and the resulting hydrogen gas (visible as fuming) was removed constantly by a gentle N_2 stream. EtSNa was obtained as a white powder and stored under N_2.

trans-2-(Ethylthio)-4-(3-styrylphenyl)-6-(trifluoromethyl)pyrimidine

A microwave vial was charged with 6.8 mg (16.8 µmol, 1.0 equiv.) of *trans*-2-(methylsulfonyl)-4-(3-styrylphenyl)-6-(trifluoromethyl)pyrimidine (*trans*-**9**) dissolved in a mixture of dry THF (3 mL) and EtSH (50 µL) before the addition of 1.4 mg (16.8 µmol, 1.0 equiv.) of EtSNa*. The reaction was heated in a microwave for 10 min at 100 °C (P = 150 W), cooled to r.t. and the volatiles removed *in vacuo* before the crude material was directly subjected to FCC (hexanes/DCM = 100/0 over 2 CV → gradient to 0/100 over 10 CV; elution ~70% DCM) to obtain 5.8 mg (15.0 µmol) of the desired product in 89% yield as a clear oil. Due to convincing characterization by ^{1}H NMR (sulfonyl methyl group replaced by ethyl thioether signature), HRMS and R_f shift, no ^{13}C was obtained.

^{1}H NMR (400 MHz, $CDCl_3$): δ [ppm] = 8.24 (t, J = 1.7 Hz, 1H), 7.99 (dt, J = 7.7, 1.4 Hz, 1H), 7.72 (dt, J = 7.9, 1.3 Hz, 1H), 7.68 (s, 1H), 7.59–7.49 (m, 3H), 7.41–7.37 (m, 2H), 7.34–7.27 (m, 1H), 7.26–7.20 (m, 2H), 3.29 (q, J = 7.3 Hz, 2H), 1.49 (t, J = 7.3 Hz, 3H).

^{19}F NMR (376 MHz, $CDCl_3$): δ [ppm] = –70.2.

HRMS (ESI): calc. for $C_{21}H_{18}F_3N_2S$ $(M+H)^+$: 387.1137, found: 387.1143.

UV/Vis (hexanes/DCM): λ_{max} = 278 nm.

R_f (hexanes/DCM = 2/1): 0.54.

2.9. 2-(Ethylsulfinyl)-4-(3-styrylphenyl)-6-(trifluoromethyl)pyrimidine (SETP)

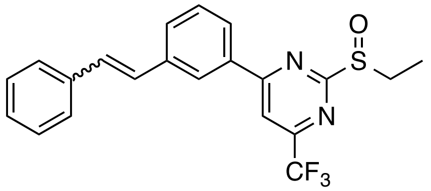

cis-2-(Ethylsulfinyl)-4-(3-styrylphenyl)-6-(trifluoromethyl)pyrimidine

A round bottom flask was charged with 9.8 mg (25.4 µmol, 1.0 equiv.) of *cis*-2-(ethylthio)-4-(3-styrylphenyl)-6-(trifluoromethyl)pyrimidine (*cis*-**10**) dissolved in DCM (5 mL) and cooled to 0 °C before the addition of 5.7 mg (25.4 µmol, 1.0 equiv.) of *m*CPBA (77%) dissolved in DCM (2 mL). The reaction was stirred in an ice bath for 90 min before the volatiles were removed *in vacuo* and the crude directly subjected to FCC (EtOAc/DCM = 0/100 over 2 CV → gradient to 0/100 over 10 CV; elution ~55% EtOAc). The product containing fractions were pooled and the solvent removed *in vacuo* and the solid subjected to RP-HPLC (*vide supra*, elution ~38 min, monitoring λ = 290 nm for product collection) to obtain 7.8 mg (18.9 µmol) of the desired product (with <10mol% TFA according to ^{19}F NMR) after freeze-drying as a yellowish solid in 74% yield.

^{1}H NMR (400 MHz, $CDCl_3$): *δ* [ppm] = 8.05 (m, 1H), 7.95 (t, *J* = 1.6 Hz, 1H), 7.67 (s, 1H), 7.51–7.38 (m, 2H), 7.31–7.23 (m, 5H), 6.78 (d, *J* = 12.1 Hz, 1H), 6.66 (d, *J* = 12.1 Hz, 1H), 3.27 (dq, *J* = 13.4, 7.4 Hz, 1H), 3.16 (dq, *J* = 13.4, 7.4 Hz, 1H), 1.34 (t, *J* = 7.4 Hz, 3H).

^{13}C NMR (101 MHz, $CDCl_3$): *δ* [ppm] = 173.8, 167.8, 156.9 (q, *J* = 36.9 Hz), 137.9, 136.7, 133.6, 133.5, 131.7, 129.1, 128.6, 128.4, 128.2, 127.8, 127.2, 126.3, 119.8 (q, *J* = 275.7 Hz), 112.3 (q, *J* = 2.9 Hz), 47.2, 6.2.

^{19}F NMR (376 MHz, $CDCl_3$): *δ* [ppm] = –69.5, –76.0.

HRMS (ESI): calc. for $C_{21}H_{17}F_3N_2NaOS$ $(M+Na)^+$: 425.0906, found: 425.0911.

UV/Vis (DCM/EtOAc): λ_{max1} = 226 nm, λ_{max2} = 290 nm.

R_f (DCM/EtOAc = 4/1): 0.70.

t_R (LCMS) = 4.364 min.

LRMS (ESI, LCMS**)**: calc. for $C_{21}H_{18}F_3N_2OS$ $(M+H)^+$: 403.1, found: 403.1.

trans-2-(Ethylsulfinyl)-4-(3-styrylphenyl)-6-(trifluoromethyl)pyrimidine

A round bottom flask was charged with 5.8 mg (16.8 µmol, 1.0 equiv.) of *trans*-2-(ethylthio)-4-(3-styrylphenyl)-6-(trifluoromethyl)pyrimidine (*trans*-**10**) dissolved in DCM (5 mL) and cooled to 0 °C before the addition of 3.8 mg (16.8 µmol, 1.0 equiv.) of *m*CPBA (77%) dissolved in DCM (2 mL). The reaction was stirred in an ice bath for 90 min before the volatiles were removed *in vacuo* and the crude directly subjected to FCC (EtOAc/DCM = 0/100 over 2 CV → gradient to 0/100 over 10 CV; elution ~55% EtOAc). The product containing fractions were pooled and the solvent removed *in vacuo* and the solid subjected to RP-HPLC (*vide supra,* elution ~38 min, monitoring λ = 290 nm for product collection) to obtain 5.9 mg (14.3 µmol) of the desired product (with <10mol% TFA according to ^{19}F NMR) as a yellowish oil after freeze-drying in 85% yield.

^{1}H NMR (400 MHz, $CDCl_3$): *δ* [ppm] = 8.33 (t, *J* = 1.8 Hz, 1H), 8.11–8.09 (m, 2H), 7.80 (dt, *J* = 7.9, 1.3 Hz, 1H), 7.60–7.54 (m, 3H), 7.39 (dd, *J* = 8.4, 6.8 Hz, 2H), 7.34–7.28 (m, 1H), 7.25 (d, *J* = 15.7 Hz, 1H), 7.20 (d, *J* = 16.6 Hz, 1H), 3.38 (dq, *J* = 13.3, 7.4 Hz, 1H), 3.28 (dq, *J* = 13.5, 7.3 Hz, 1H), 1.41 (t, *J* = 7.4 Hz, 3H).

^{13}C NMR (101 MHz, $CDCl_3$): *δ* [ppm] = 174.0, 168.3, 157.4 (q, *J* = 36.9 Hz), 138.9, 136.7, 134.6, 130.7, 130.6, 129.8, 128.8, 128.2, 127.2, 126.9, 126.7, 126.1, 120.2 (q, *J* = 276.0 Hz), 112.8, 47.6, 6.6.

^{19}F NMR (376 MHz, $CDCl_3$): *δ* [ppm] = –69.5, –76.0.

HRMS (ESI): calc. for $C_{21}H_{17}F_3N_2NaOS$ $(M+Na)^+$: 425.0906, found: 425.0911.

UV/Vis (DCM/EtOAc): λ_{max1} = 230 nm, λ_{max2} = 296 nm.

R$_f$ (DCM/EtOAc = 4/1): 0.62.

t$_R$ (LCMS) = 4.281 min.

LRMS (ESI, LCMS**)**: calc. for $C_{42}H_{35}F_6N_4O_2S_2$ $(2M+H)^+$: 805.2, found: 805.1.

3. *Spectral data*

3.1. 4-(3-(Benzyloxy)phenyl)-2-(methylthio)-6-(trifluoromethyl)pyrimidine (3)

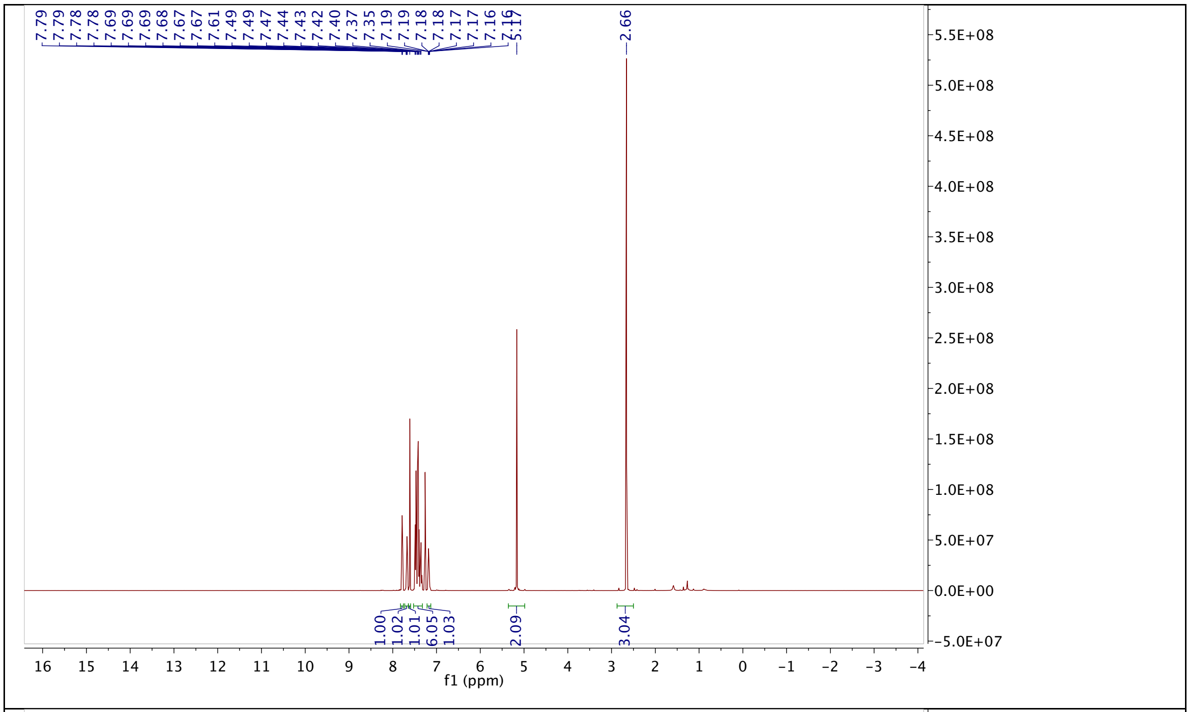

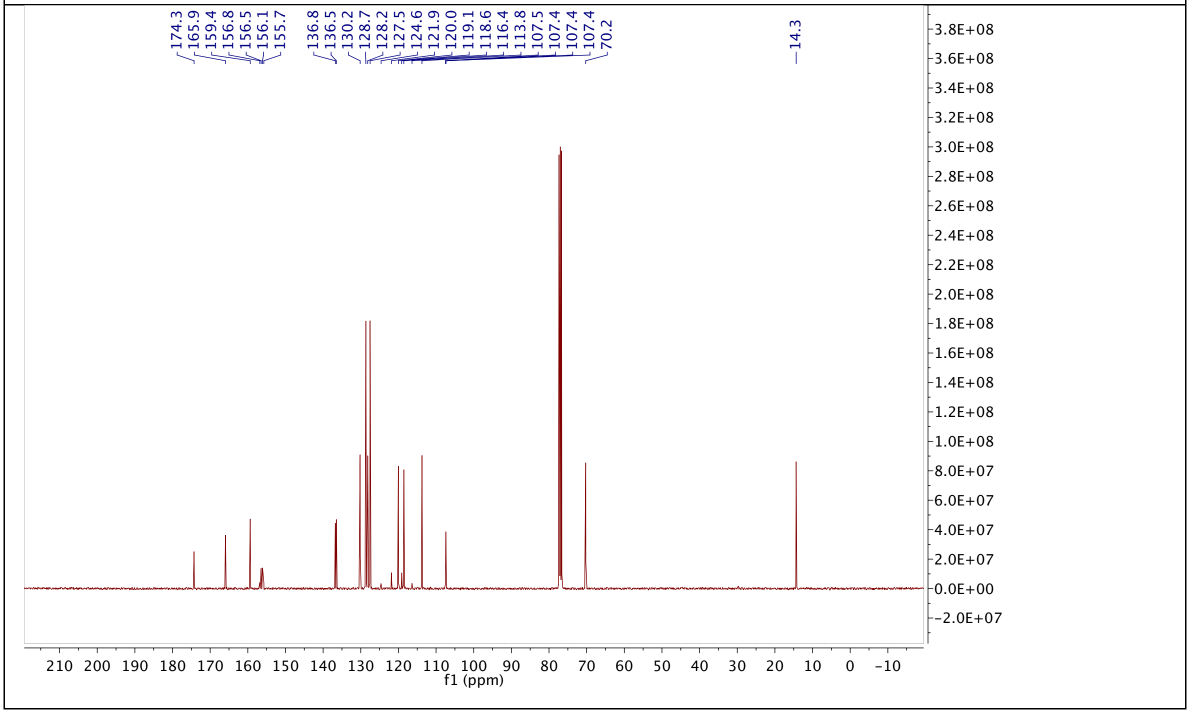

3.2. 4-(3-(Benzyloxy)phenyl)-2-(methylsulfinyl)-6-(trifluoromethyl)pyrimidine (BMTP)

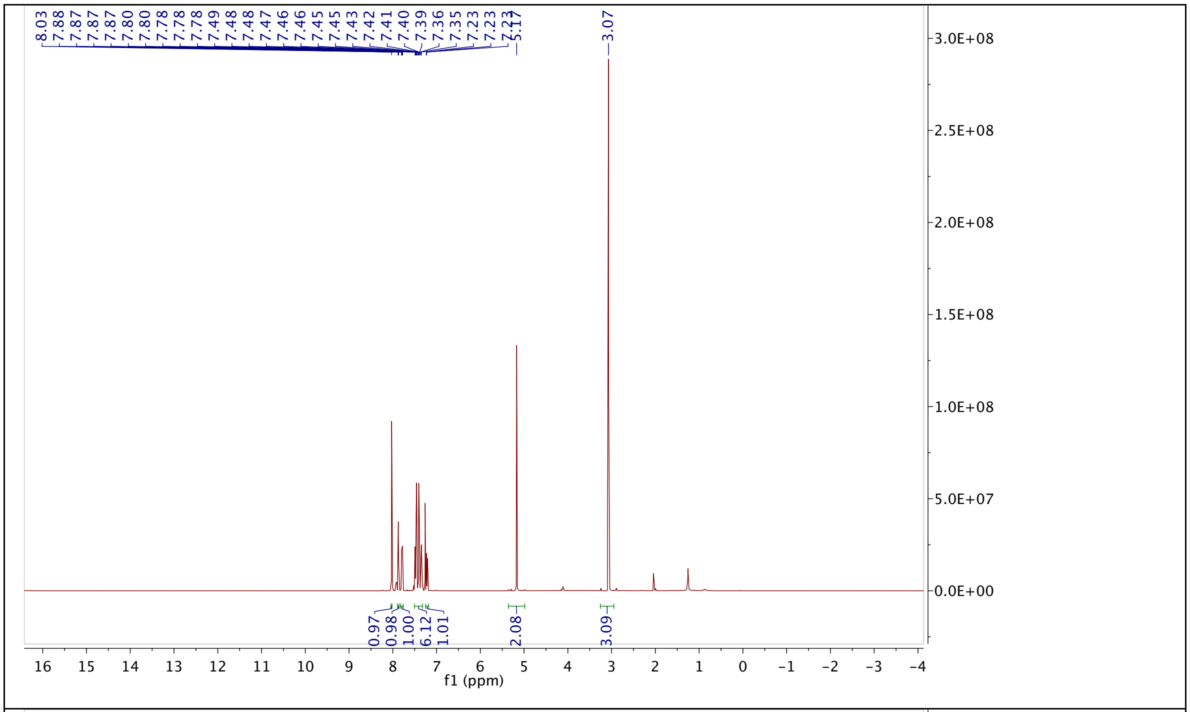

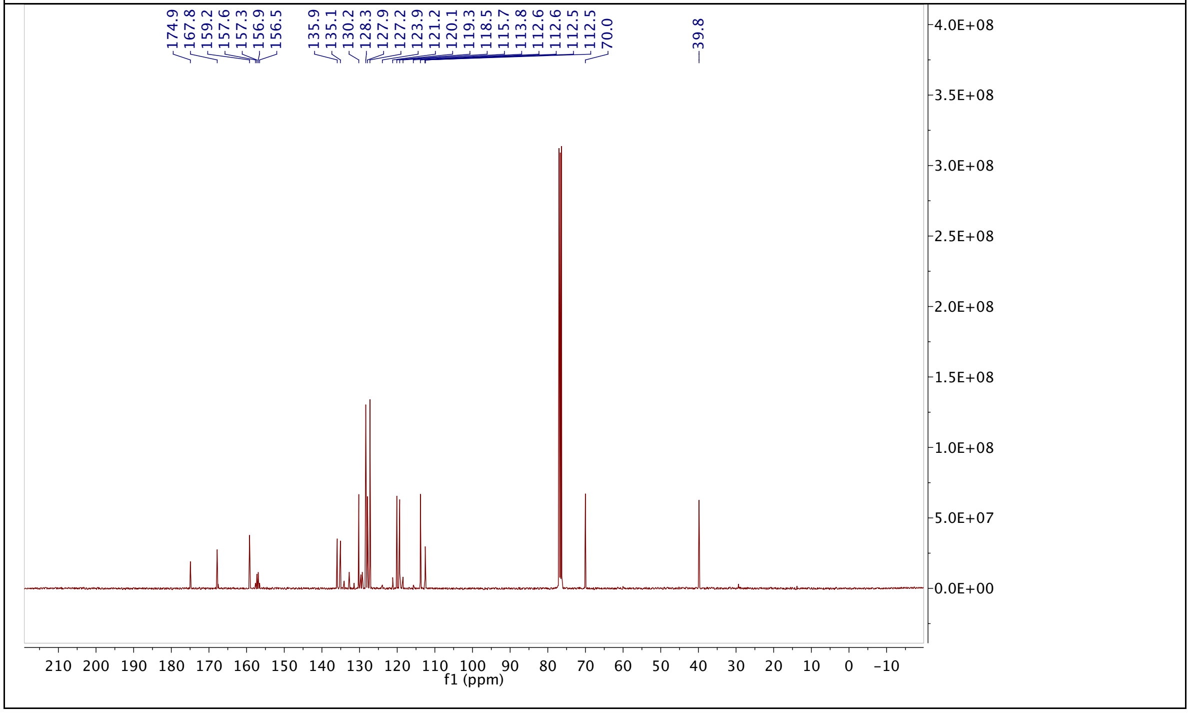

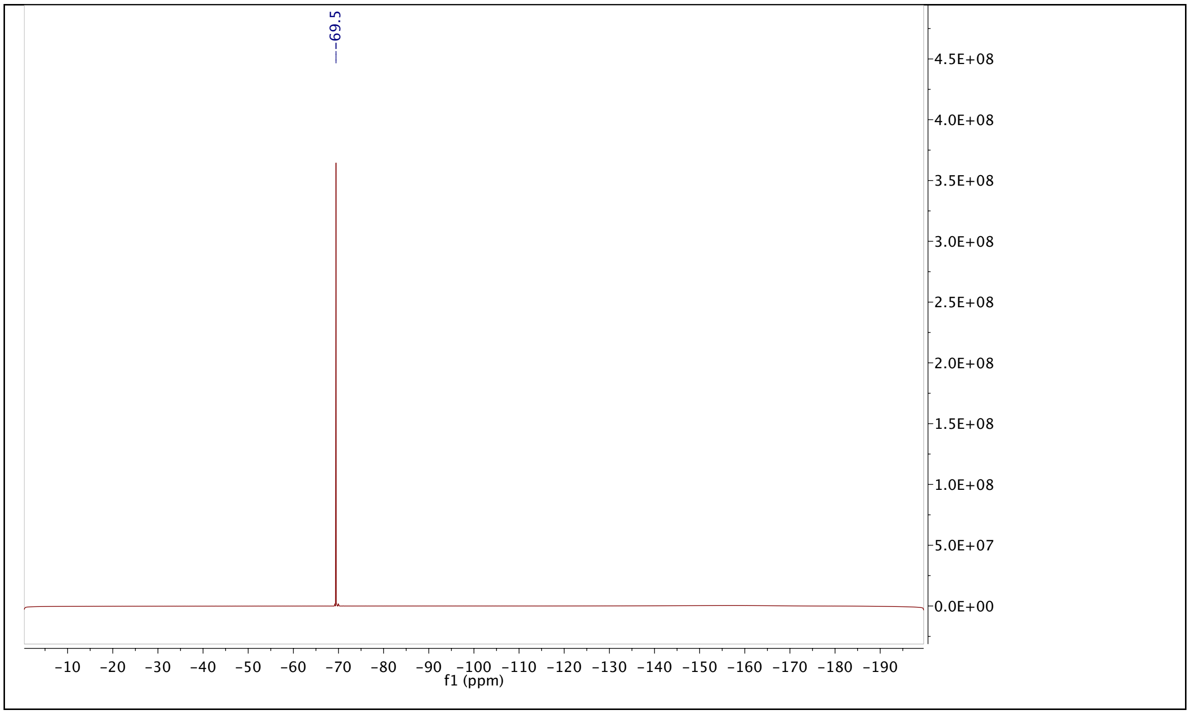

--69.5
4.5E+08
4.0E+08
3.5E+08
3.0E+08
2.5E+08
2.0E+08
1.5E+08
1.0E+08
5.0E+07
0.0E+00
−10 −20 −30 −40 −50 −60 −70 −80 −90 −100 −110 −120 −130 −140 −150 −160 −170 −180 −190
f1 (ppm)

3.3. *cis*-1-Bromo-3-styrylbenzene (*cis*-6)

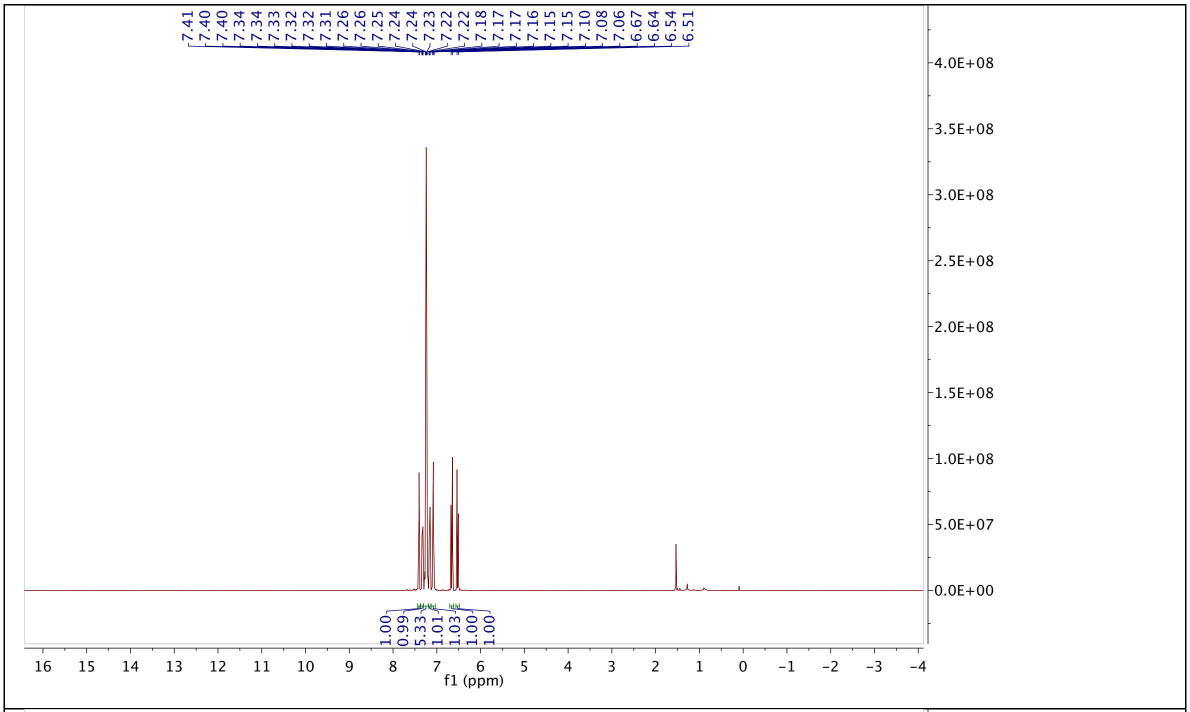

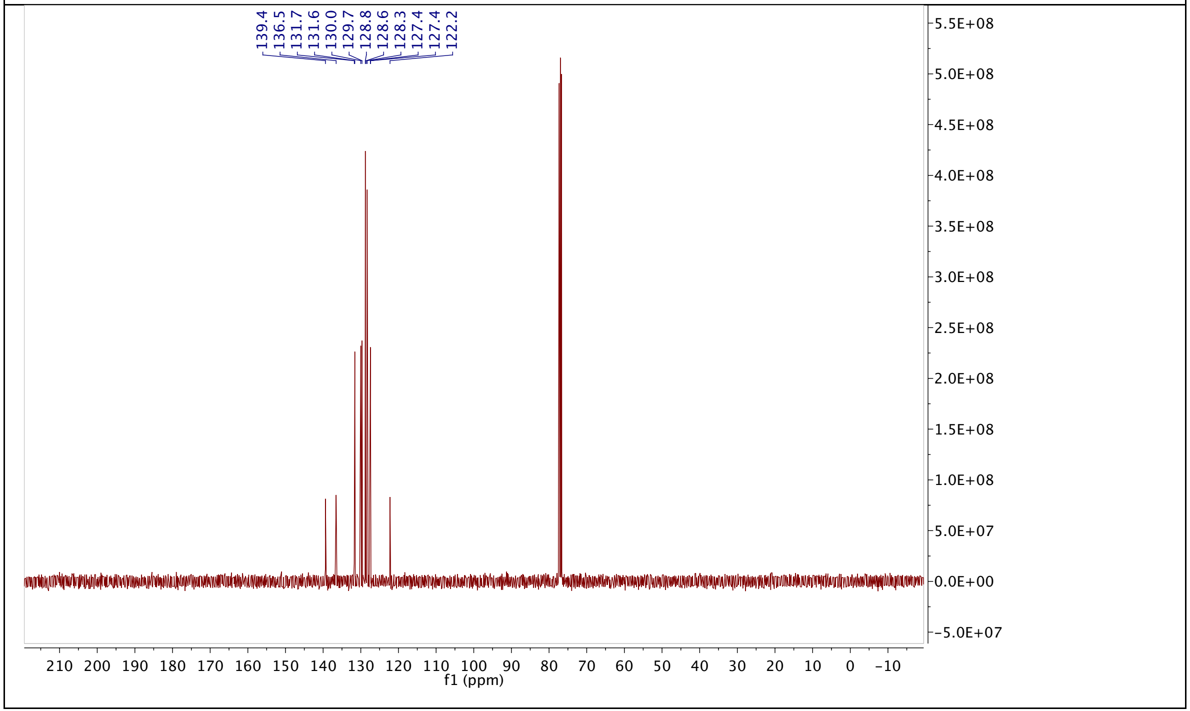

3.4. *trans*-1-Bromo-3-styrylbenzene (*trans*-6)

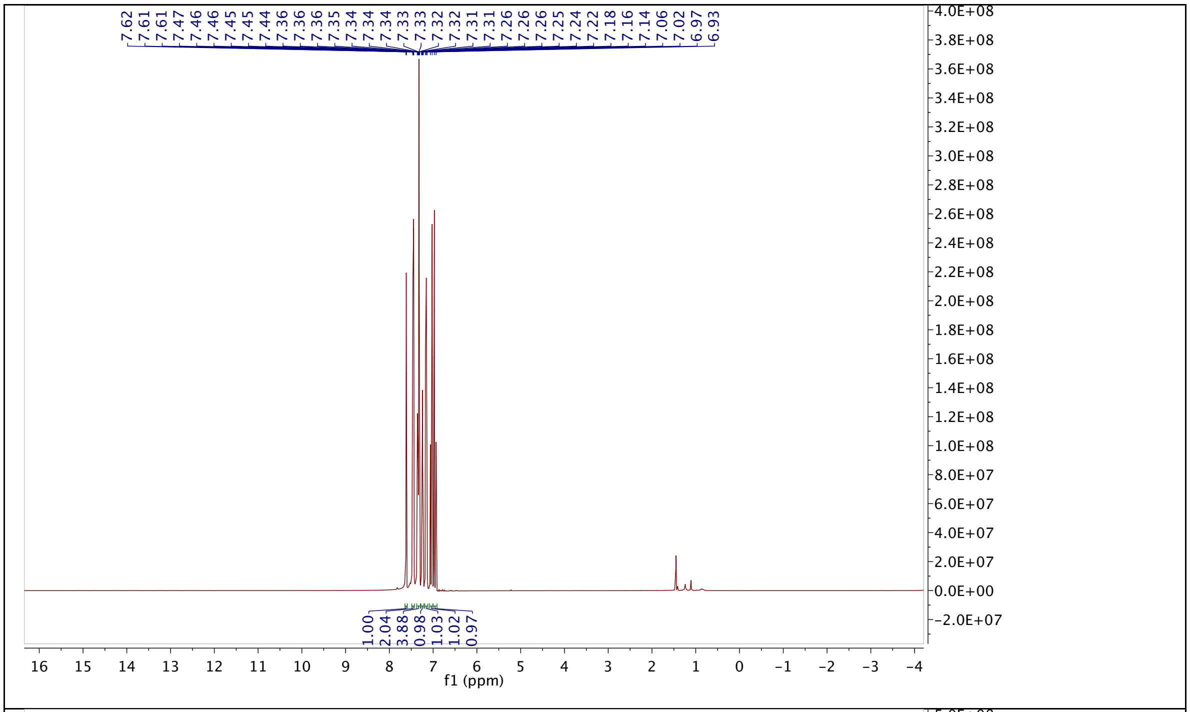

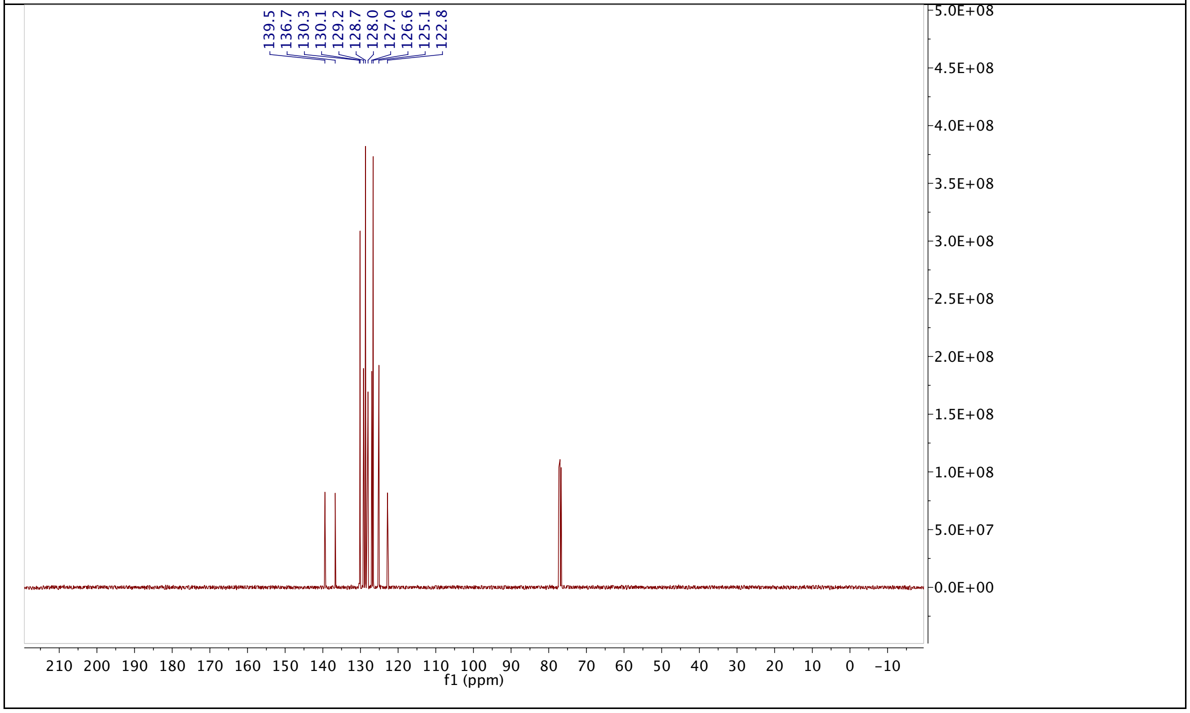

3.5. *cis*-4,4,5,5-Tetramethyl-2-(3-styrylphenyl)-1,3,2-dioxaborolane (*cis*-7)

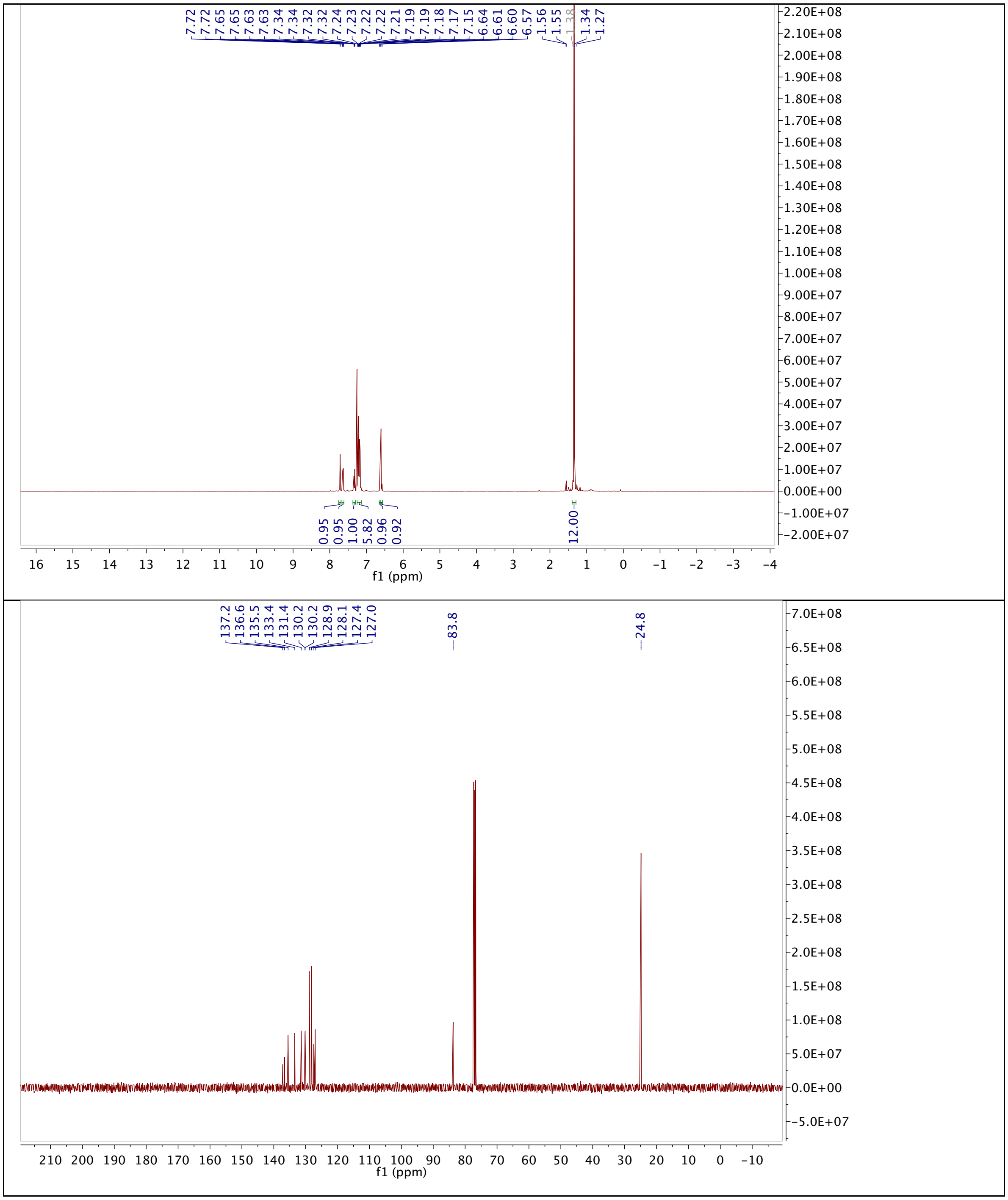

3.6. *trans*-4,4,5,5-Tetramethyl-2-(3-styrylphenyl)-1,3,2-dioxaborolane (*trans*-7)

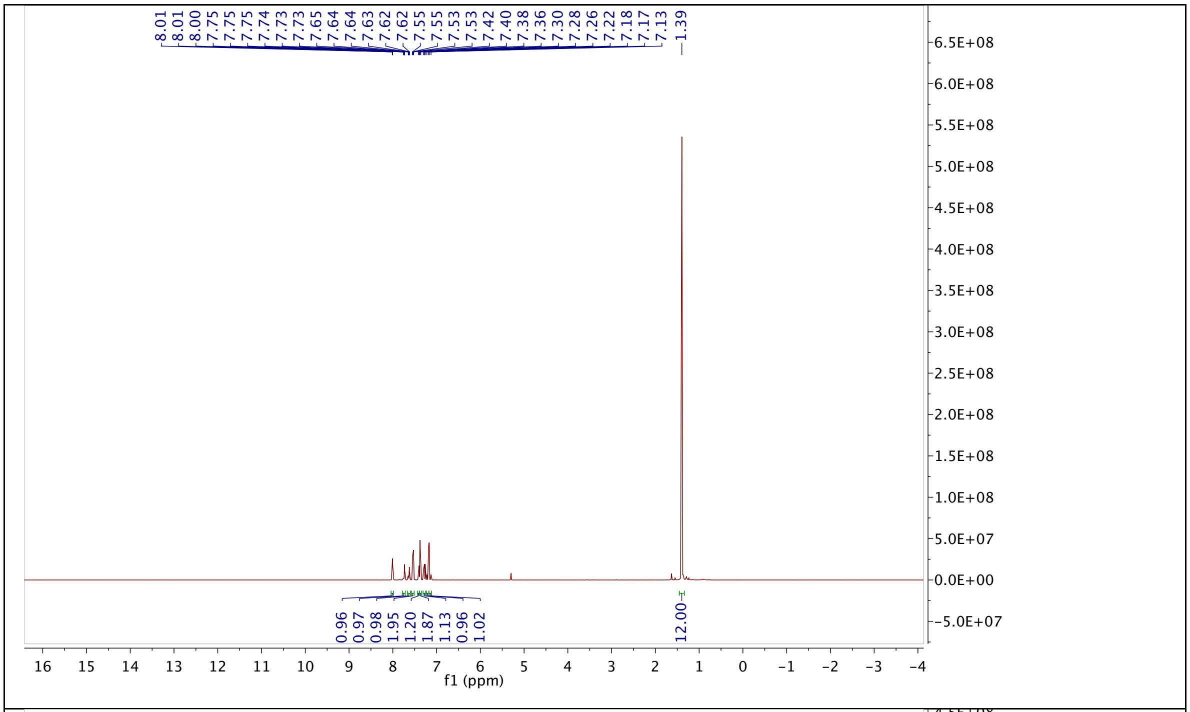

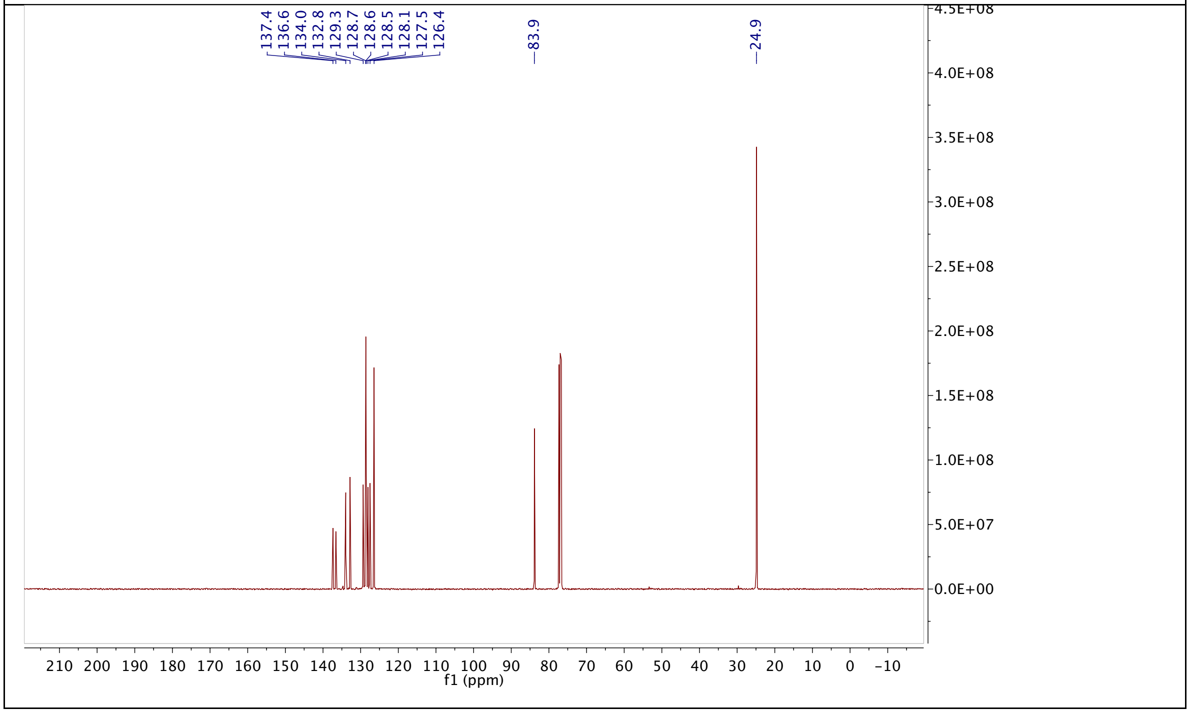

3.7. *cis*-2-(Methylthio)-4-(3-styrylphenyl)-6-(trifluoromethyl)pyrimidine (*cis*-8)

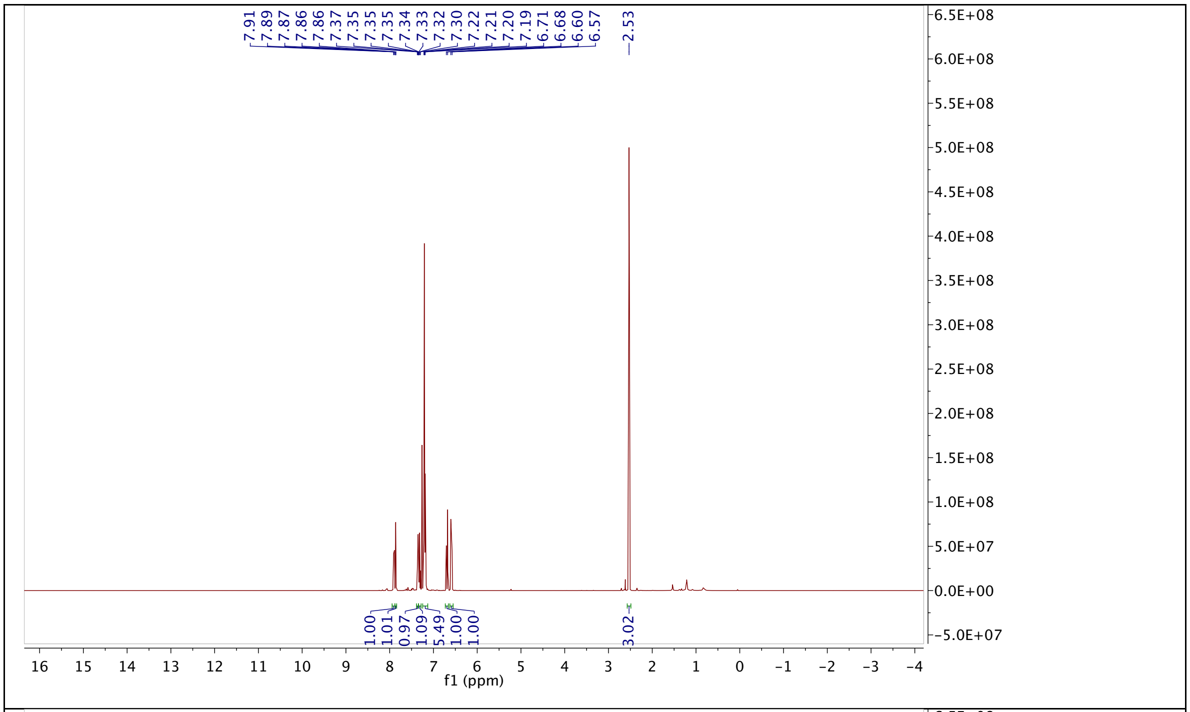

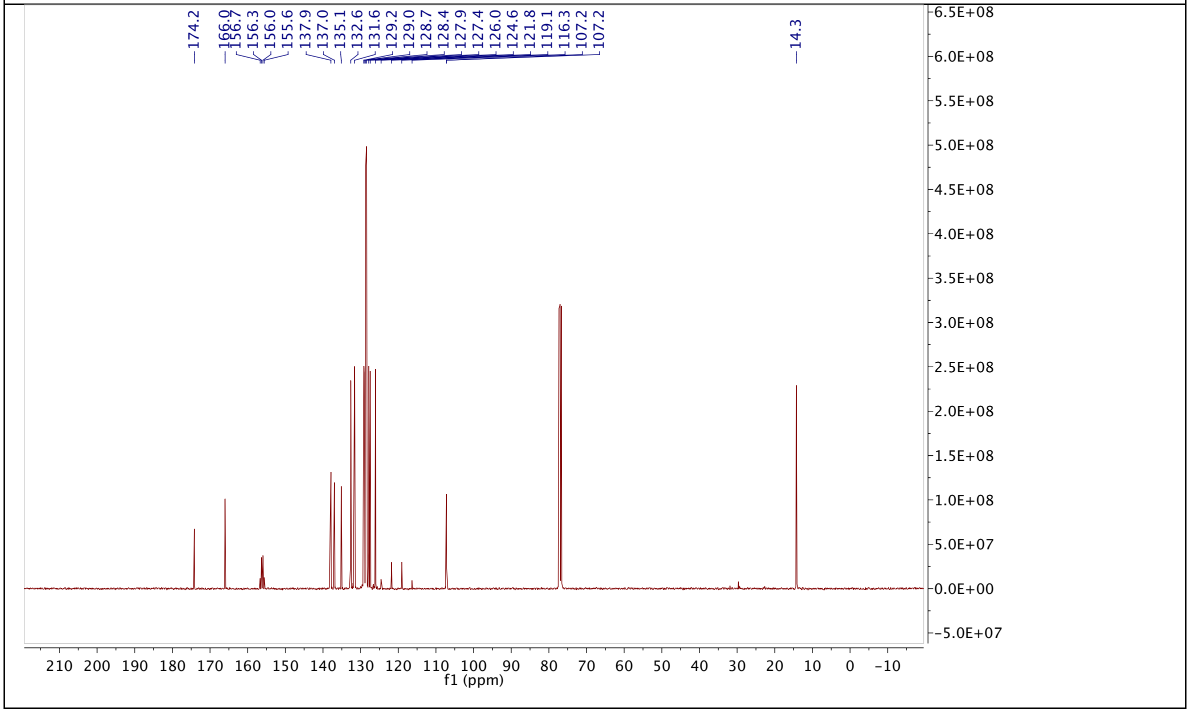

3.8. *trans*-2-(Methylthio)-4-(3-styrylphenyl)-6-(trifluoromethyl)pyrimidine (*trans*-8)

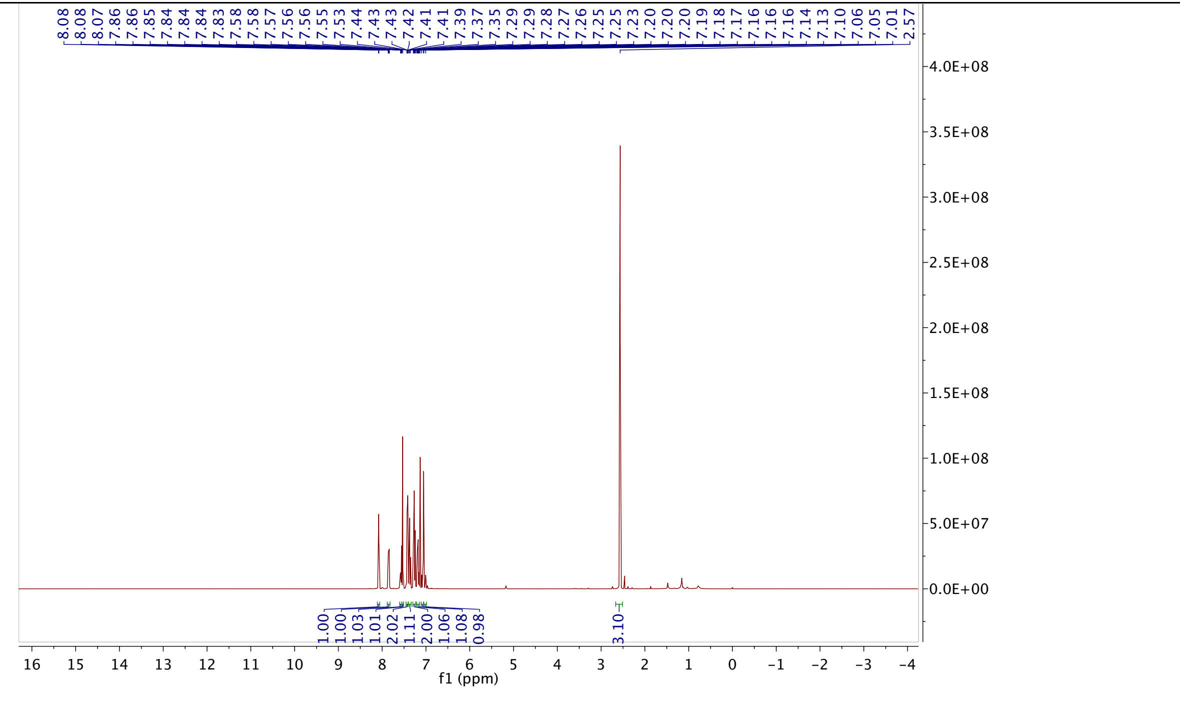

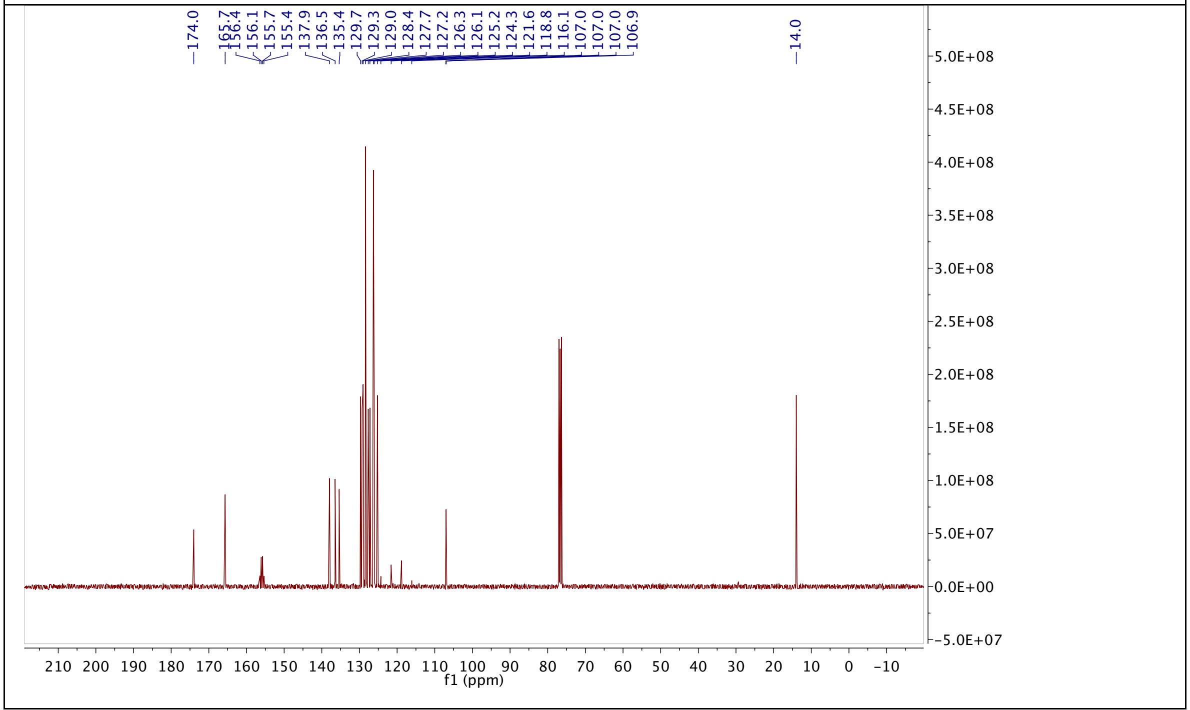

3.9. *cis*-2-(Methylsulfinyl)-4-(3-styrylphenyl)-6-(trifluoromethyl)pyrimidine (*cis*-SMTP)

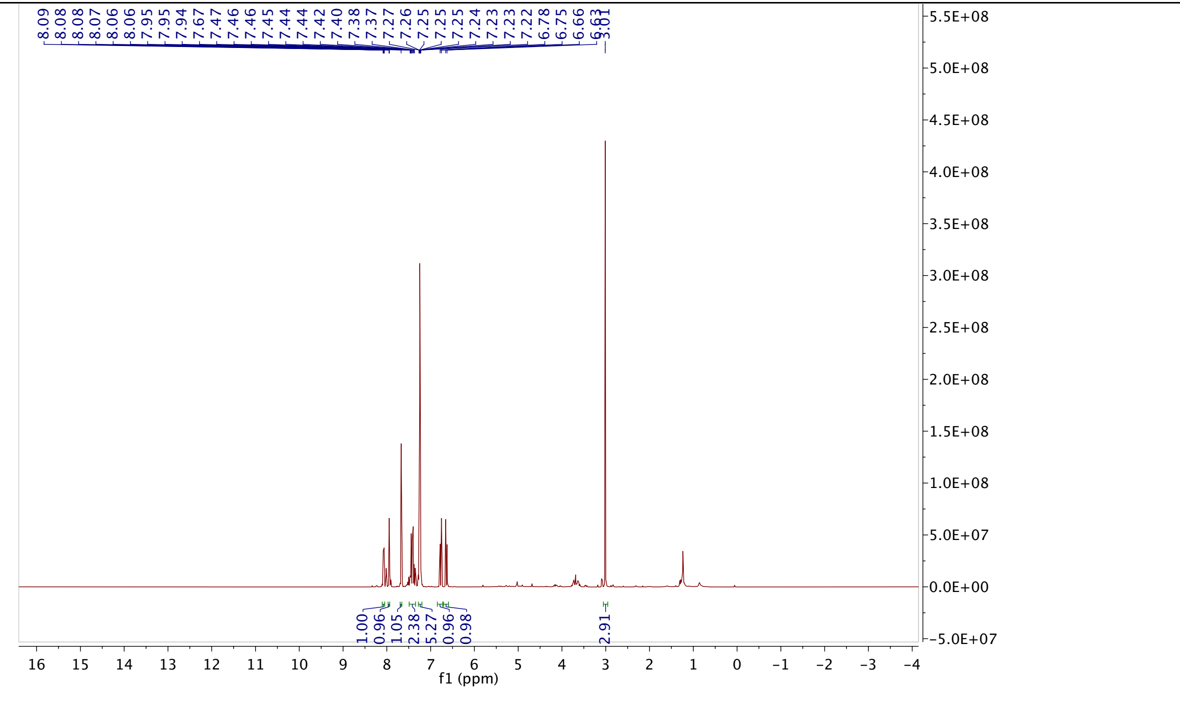

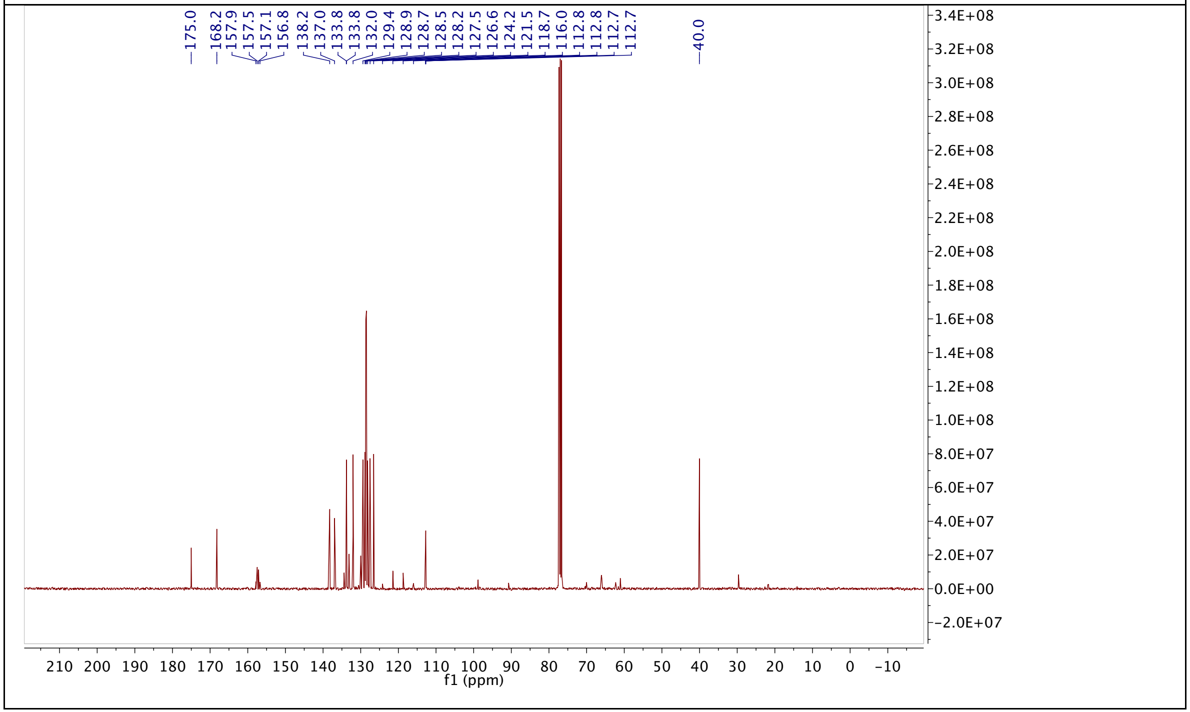

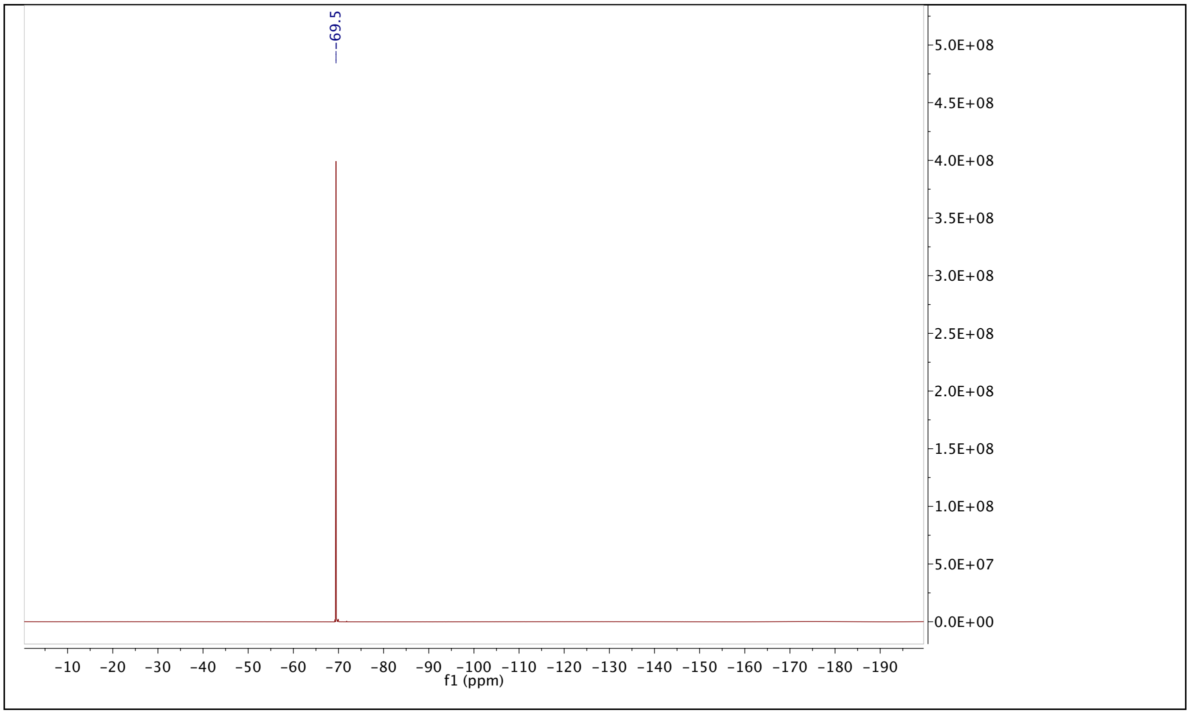

-69.5
5.0E+08
4.5E+08
4.0E+08
3.5E+08
3.0E+08
2.5E+08
2.0E+08
1.5E+08
1.0E+08
5.0E+07
0.0E+00
-10 -20 -30 -40 -50 -60 -70 -80 -90 -100 -110 -120 -130 -140 -150 -160 -170 -180 -190
f1 (ppm)

3.10. *trans*-2-(Methylsulfinyl)-4-(3-styrylphenyl)-6-(trifluoromethyl)pyrimidine (*trans*-SMTP)

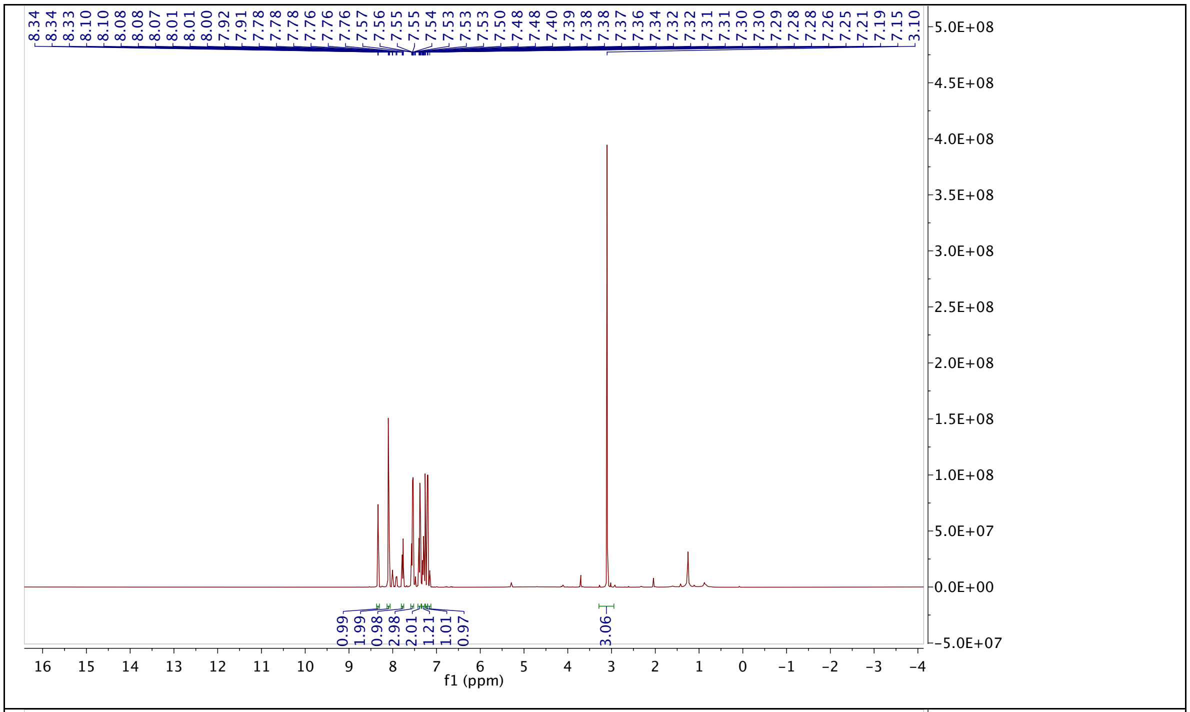

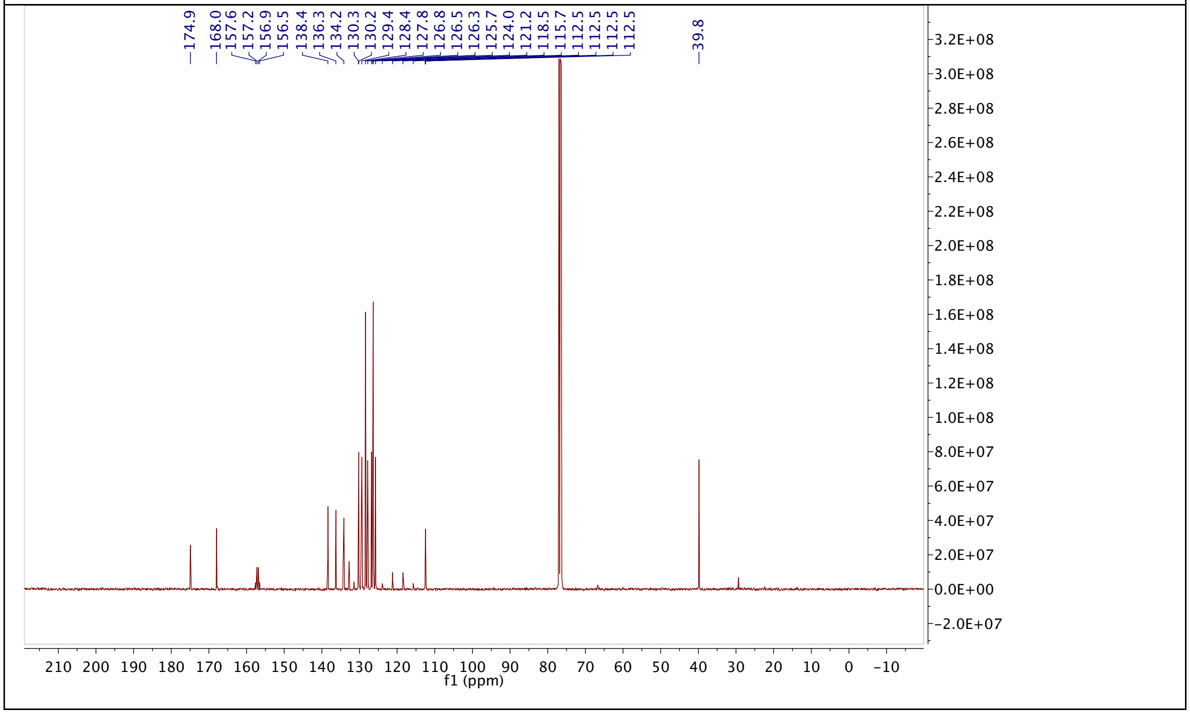

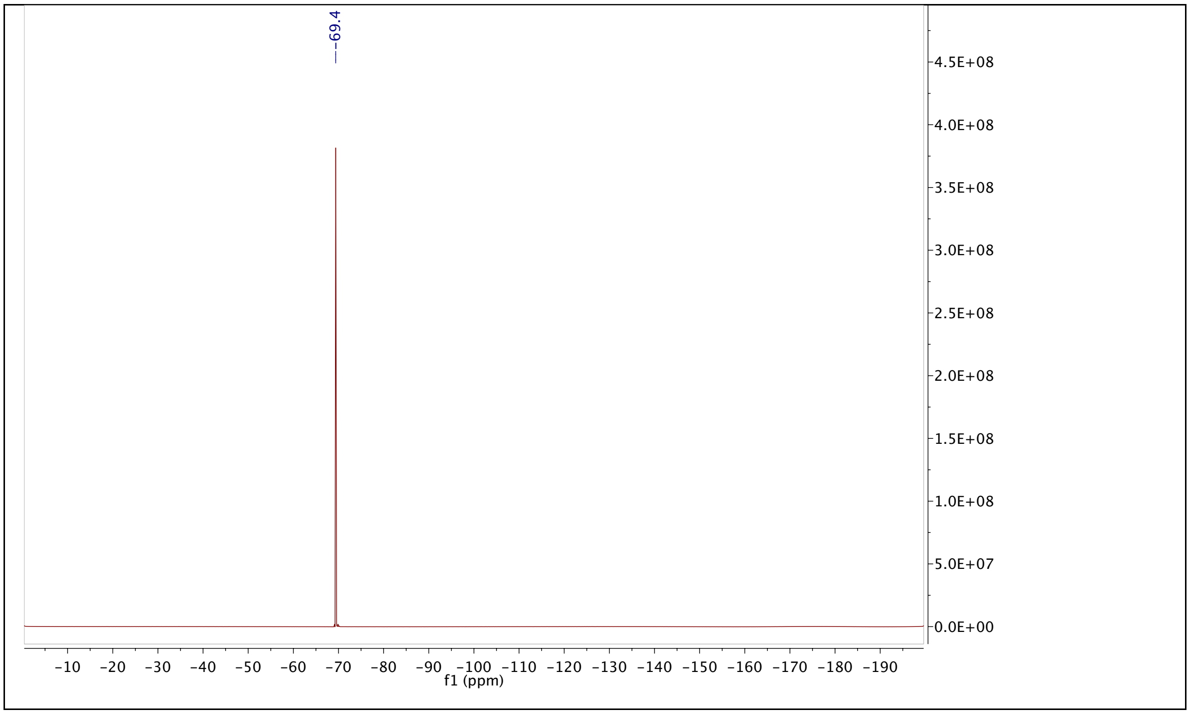
-69.4
4.5E+08
4.0E+08
3.5E+08
3.0E+08
2.5E+08
2.0E+08
1.5E+08
1.0E+08
5.0E+07
0.0E+00
-10 -20 -30 -40 -50 -60 -70 -80 -90 -100 -110 -120 -130 -140 -150 -160 -170 -180 -190
f1 (ppm)

3.11. *cis*-2-(Methylsulfonyl)-4-(3-styrylphenyl)-6-(trifluoromethyl)pyrimidine (*cis*-9)

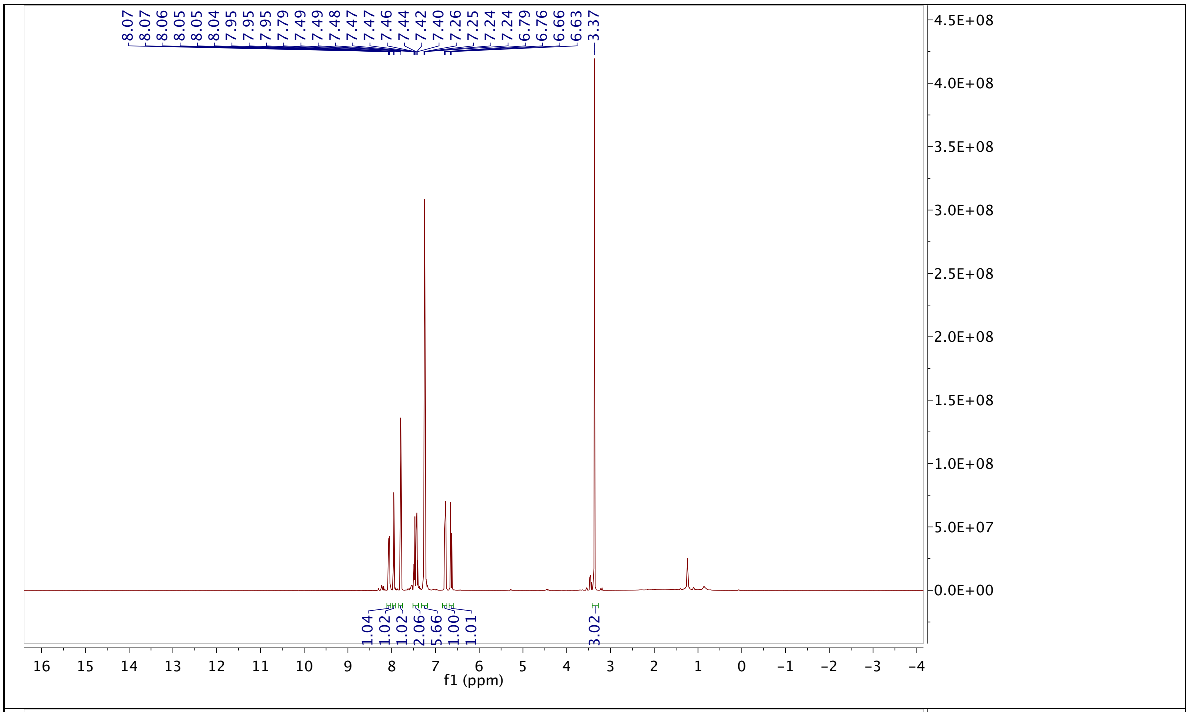

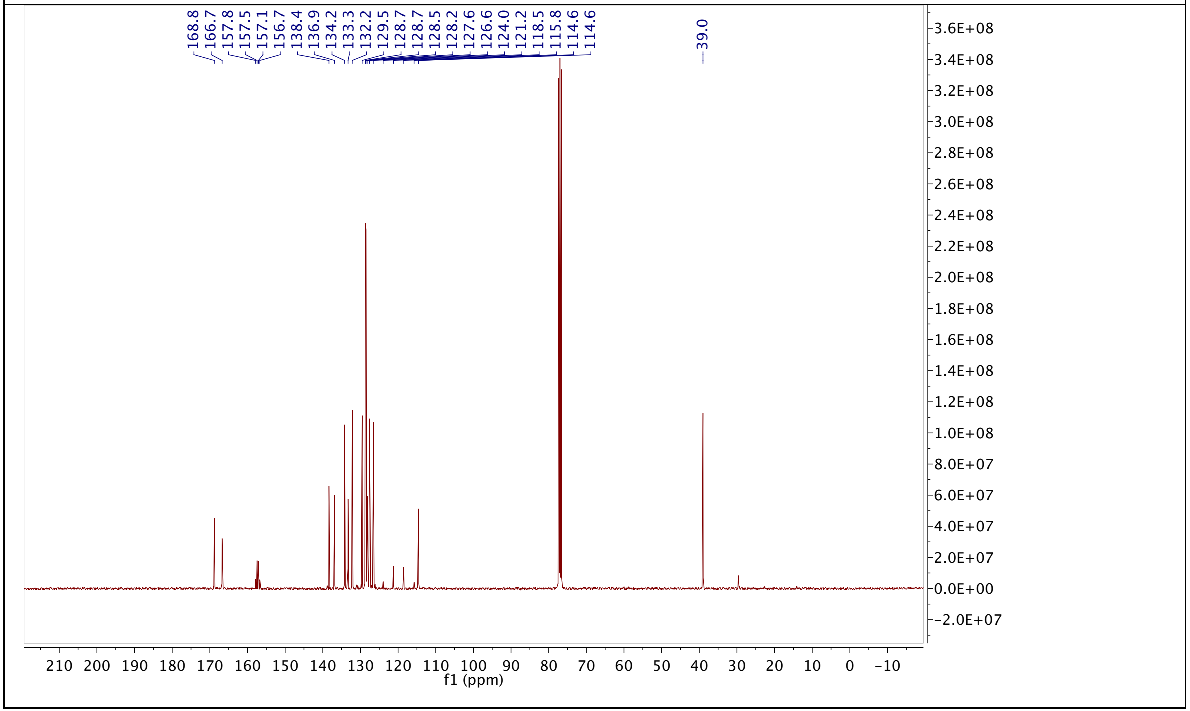

3.12. *trans*-2-(Methylsulfonyl)-4-(3-styrylphenyl)-6-(trifluoromethyl)pyrimidine (*trans*-9)

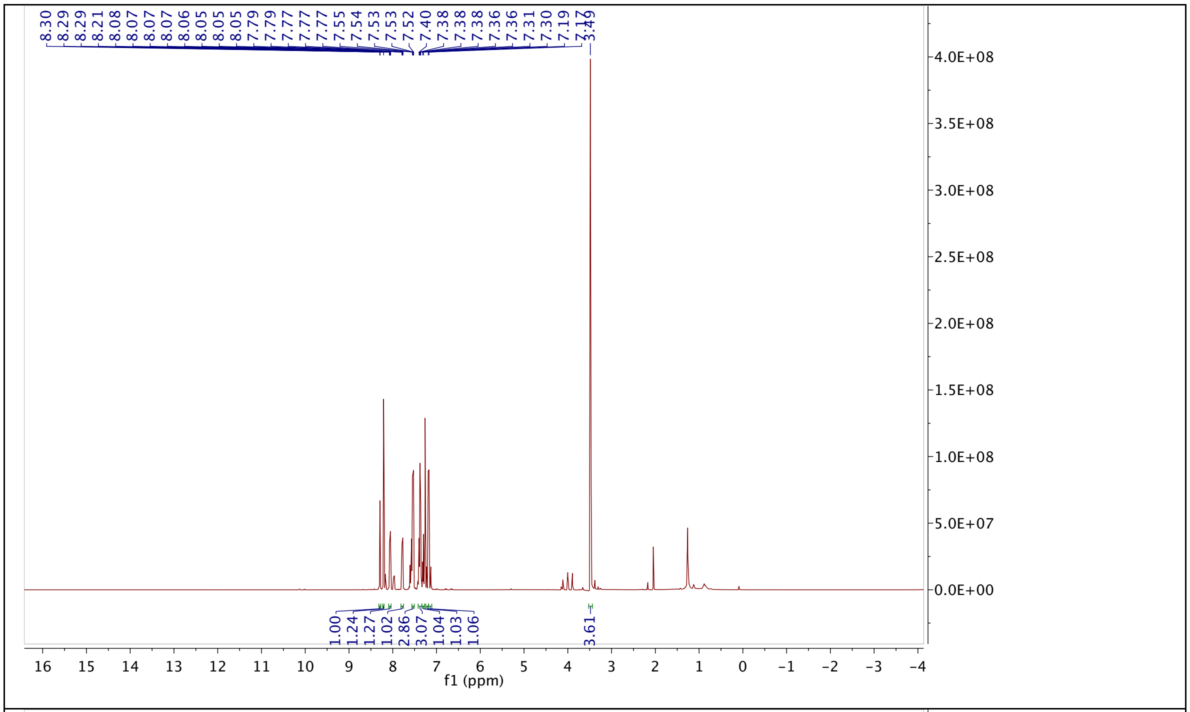

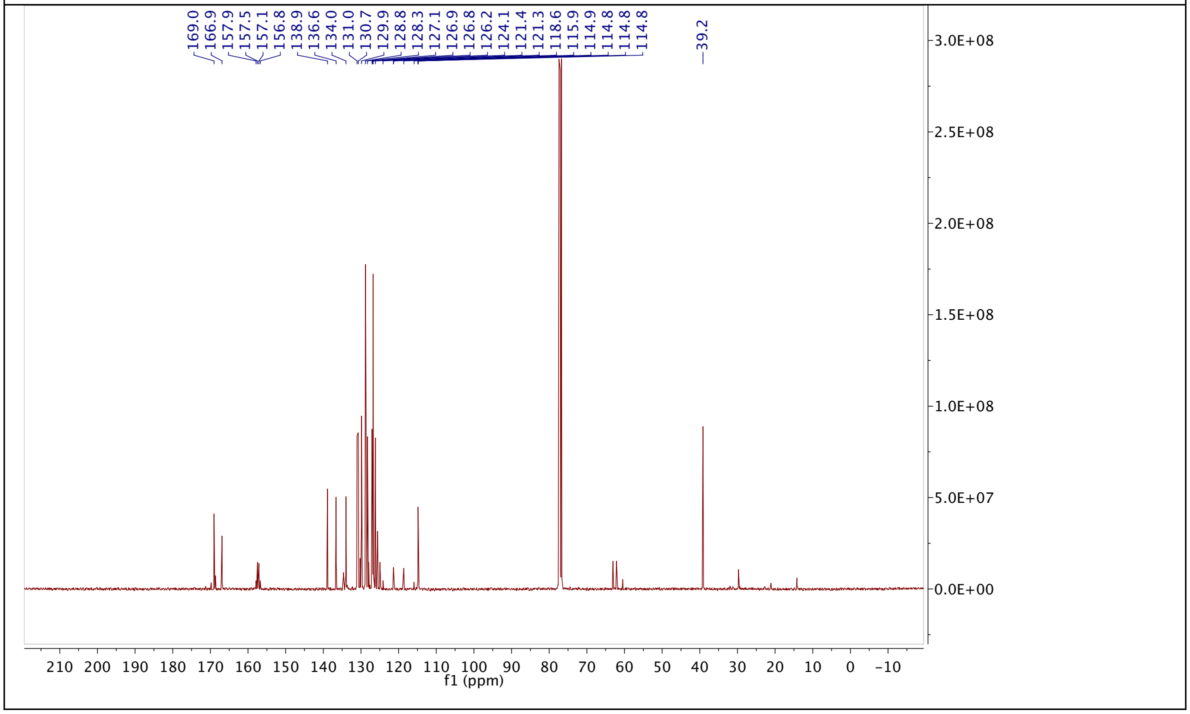

3.13. *cis*-2-(Ethylthio)-4-(3-styrylphenyl)-6-(trifluoromethyl)pyrimidine (*cis*-10)

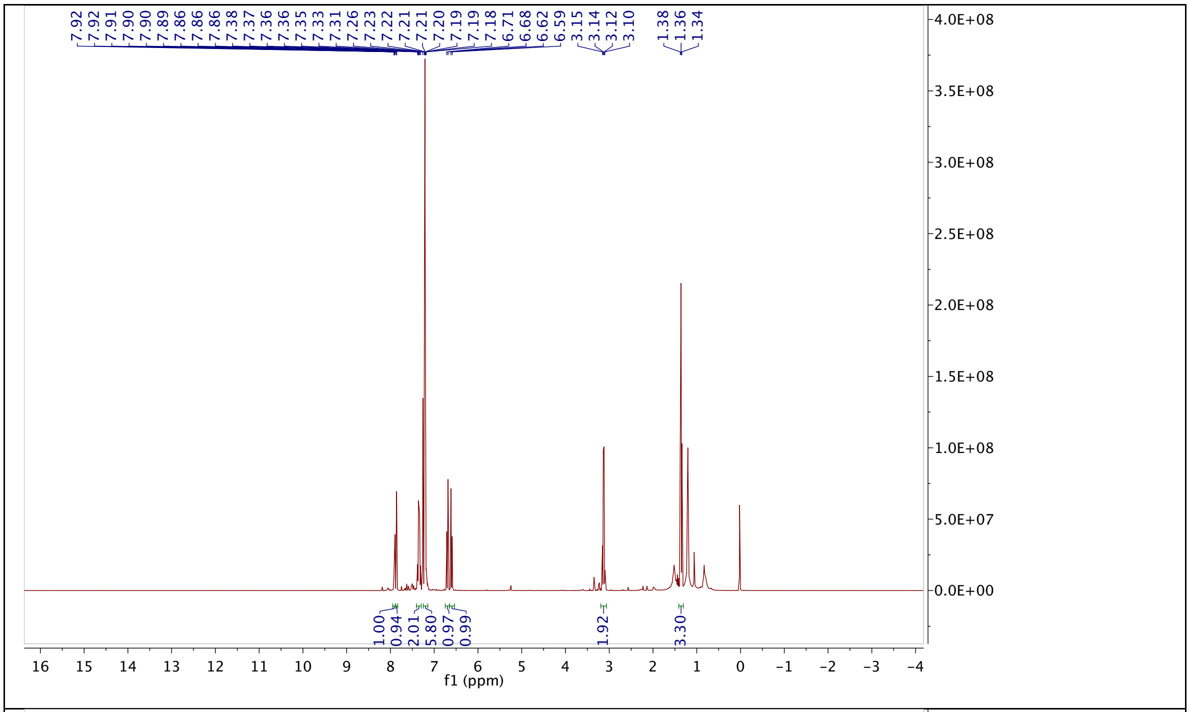

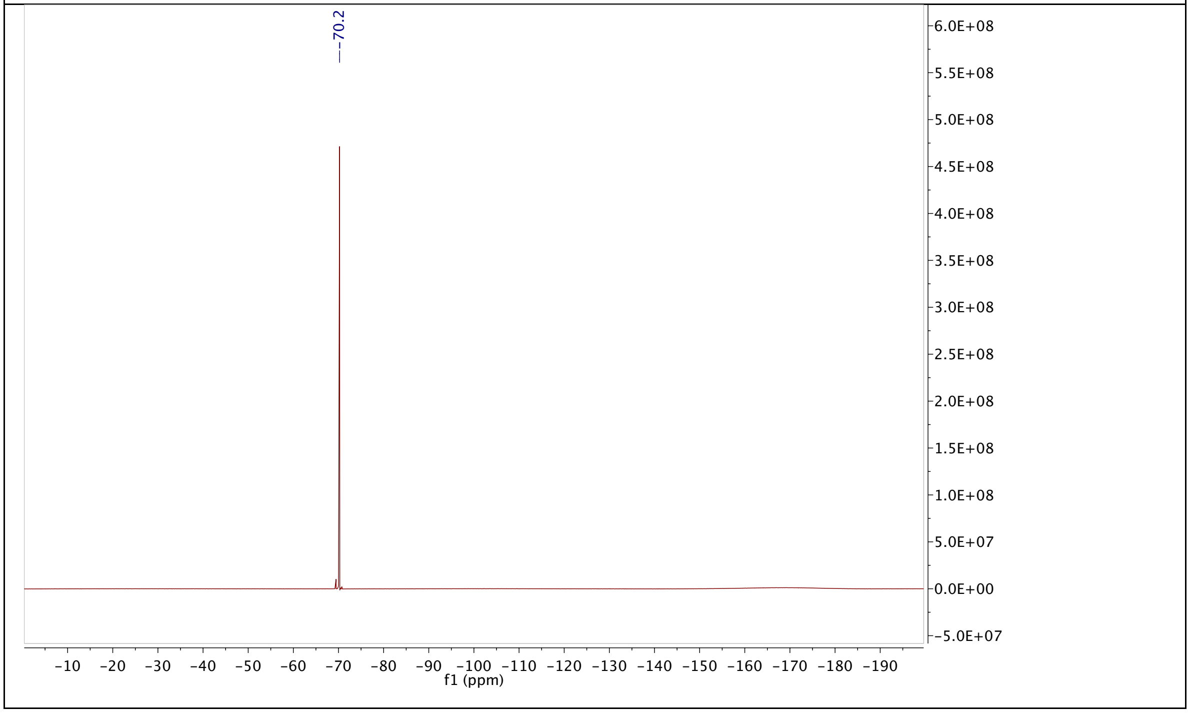

3.14. *trans*-2-(Ethylthio)-4-(3-styrylphenyl)-6-(trifluoromethyl)pyrimidine (*trans*-10)

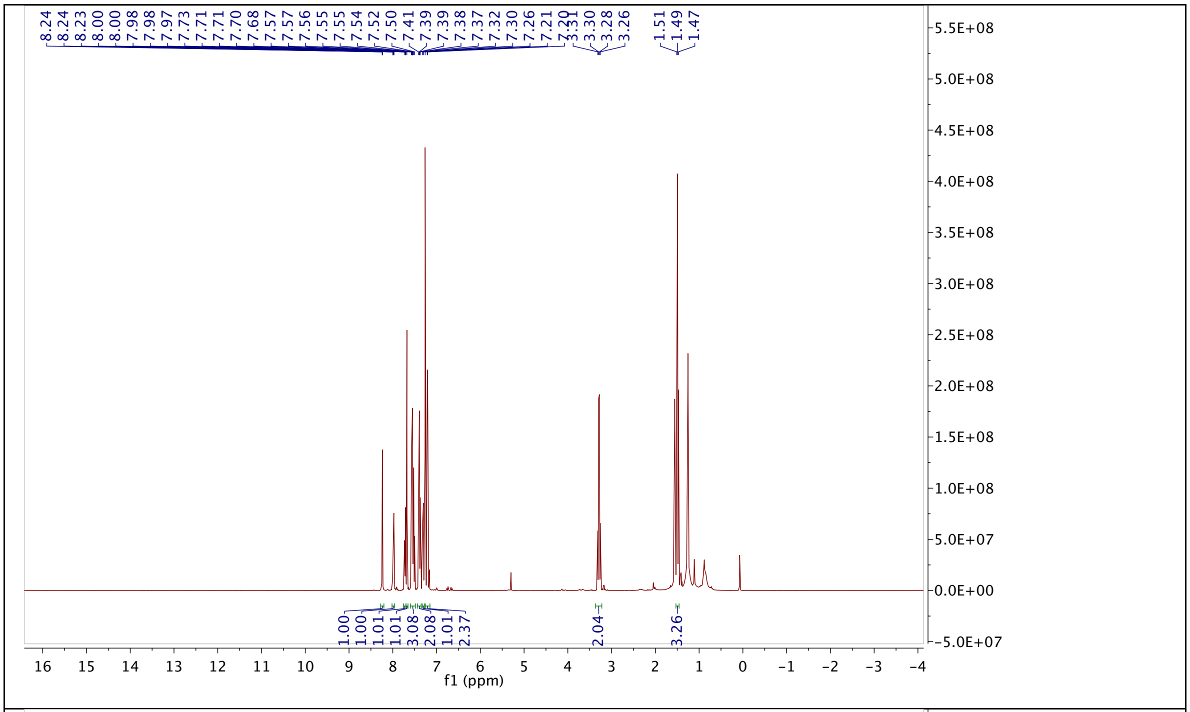

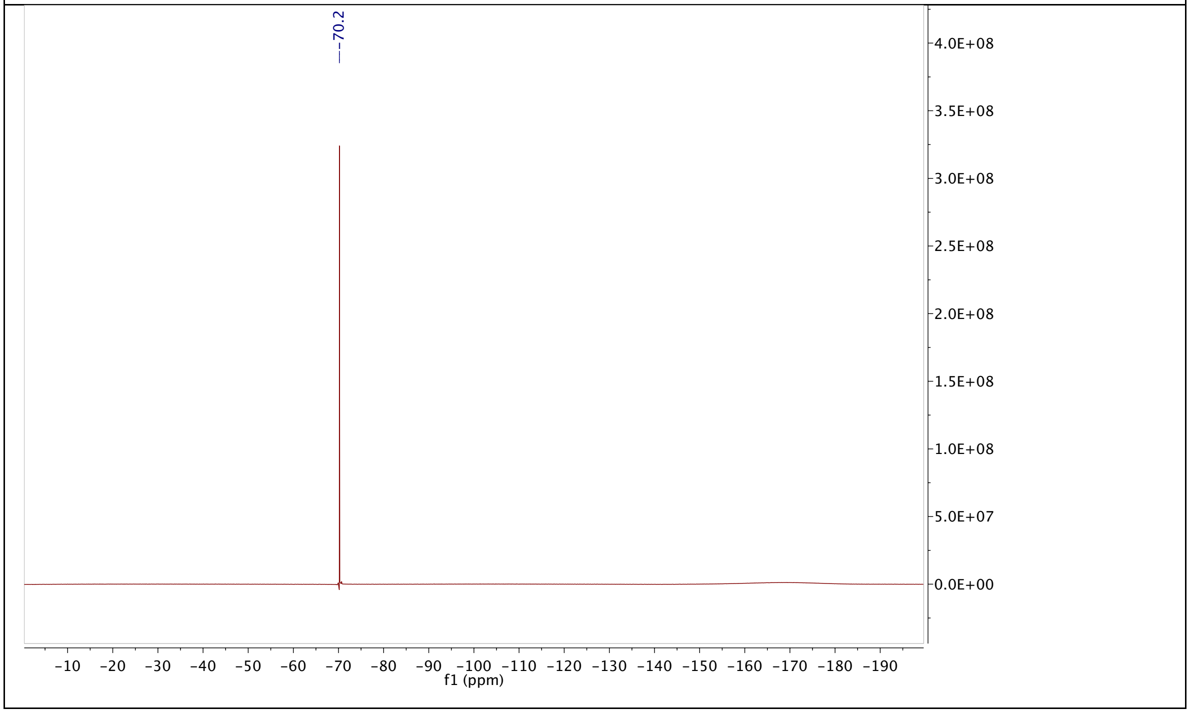

3.15. *cis*-2-(Ethylsulfinyl)-4-(3-styrylphenyl)-6-(trifluoromethyl)pyrimidine (*cis*-SETP)

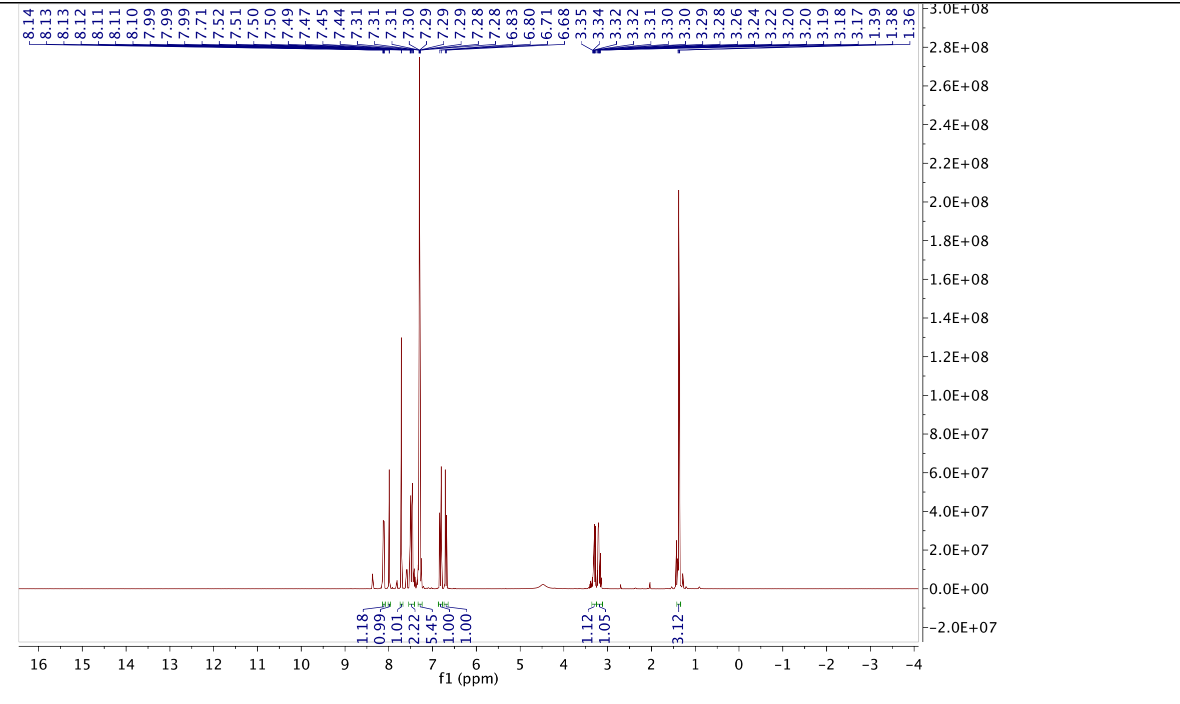

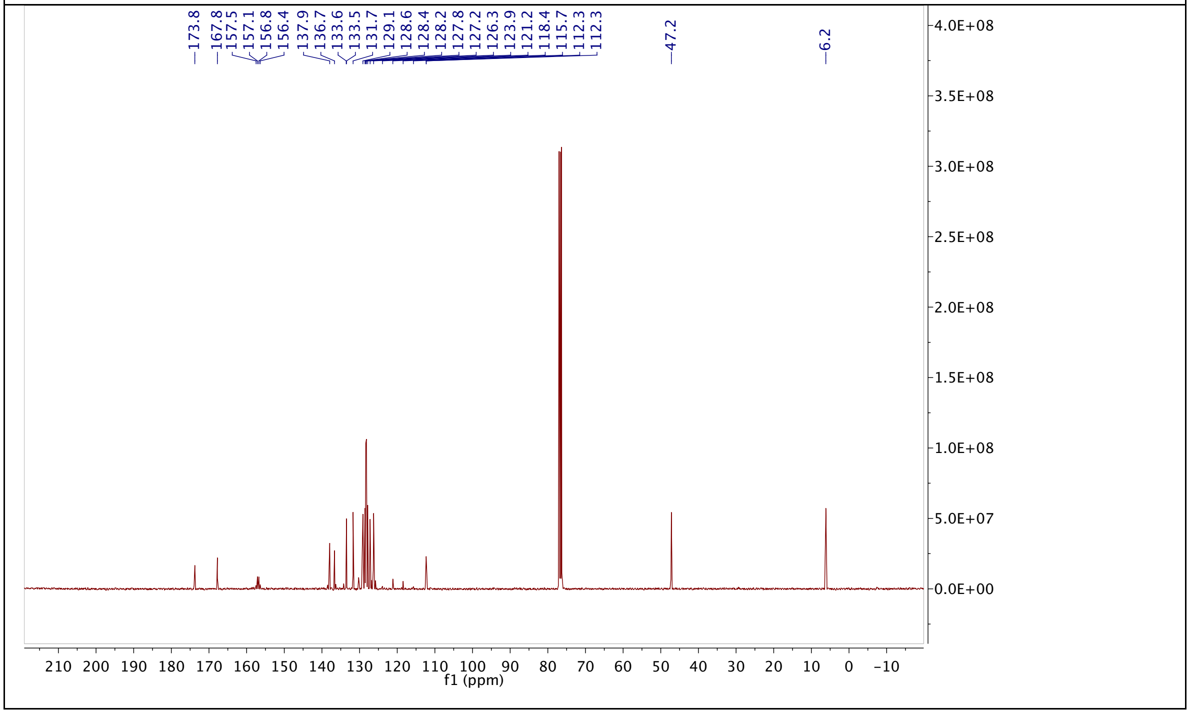

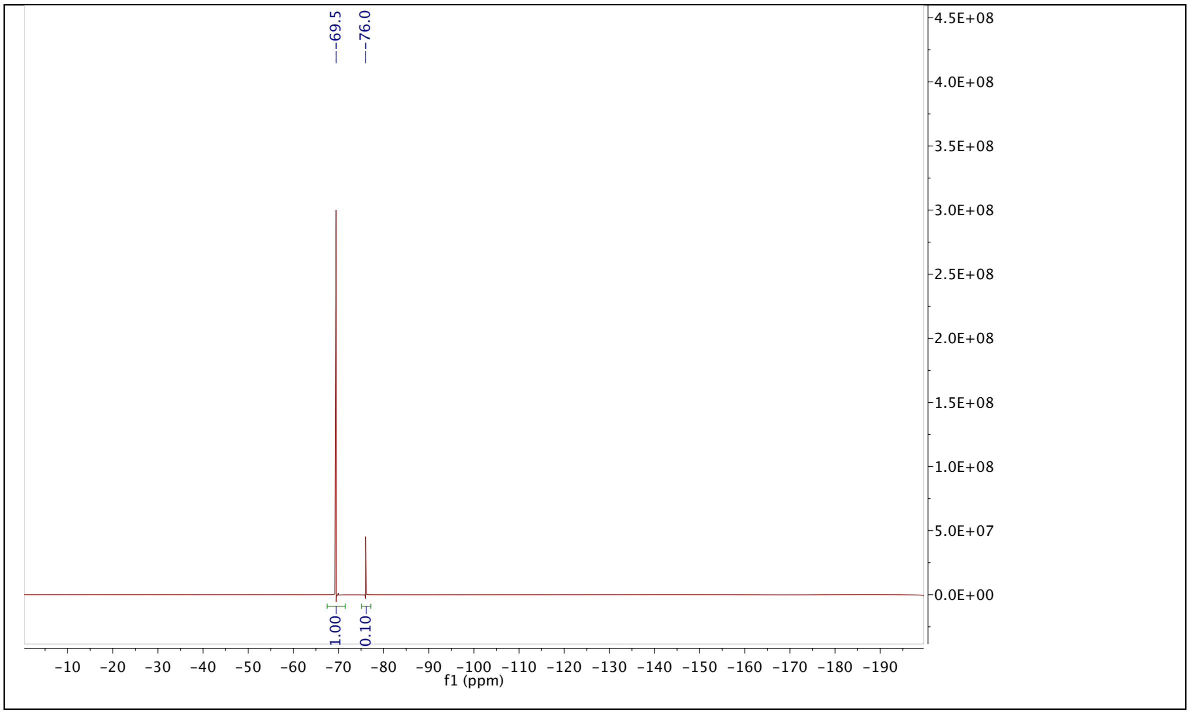

-69.5
-76.0
1.00
0.10
4.5E+08
4.0E+08
3.5E+08
3.0E+08
2.5E+08
2.0E+08
1.5E+08
1.0E+08
5.0E+07
0.0E+00
-10 -20 -30 -40 -50 -60 -70 -80 -90 -100 -110 -120 -130 -140 -150 -160 -170 -180 -190
f1 (ppm)

3.16. *trans*-2-(Ethylsulfinyl)-4-(3-styrylphenyl)-6-(trifluoromethyl)pyrimidine (*trans*-SETP)

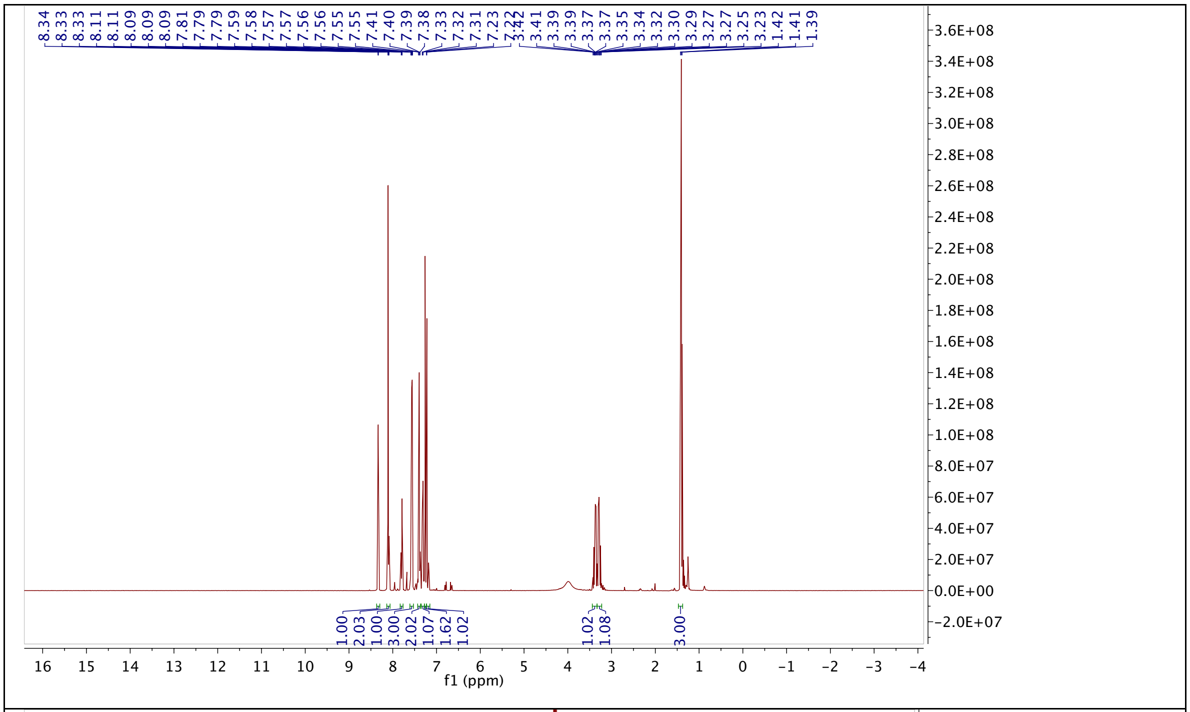

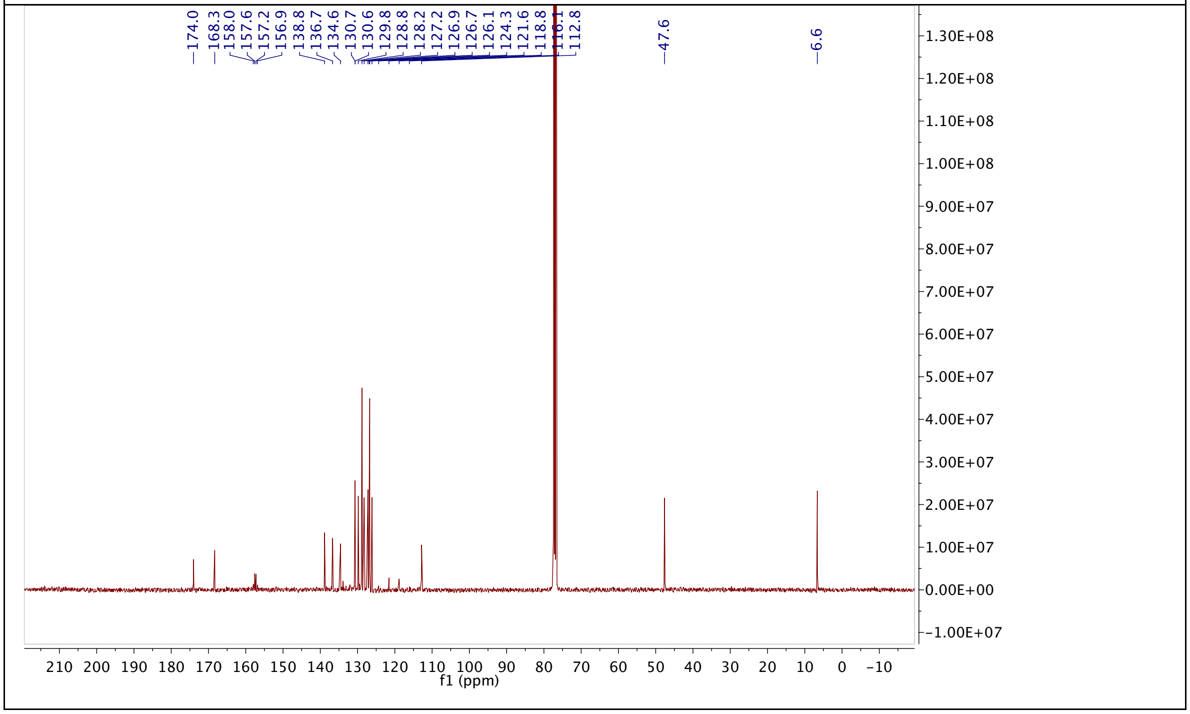

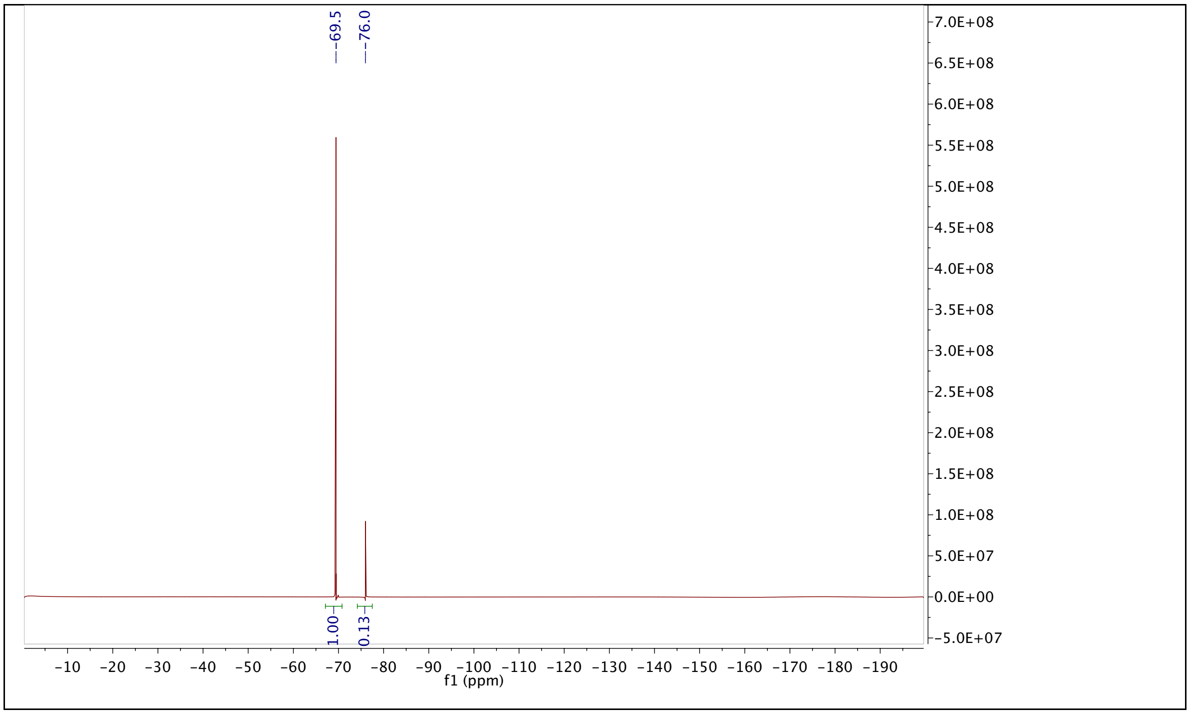

-69.5
-76.0
1.00
0.13
7.0E+08
6.5E+08
6.0E+08
5.5E+08
5.0E+08
4.5E+08
4.0E+08
3.5E+08
3.0E+08
2.5E+08
2.0E+08
1.5E+08
1.0E+08
5.0E+07
0.0E+00
-5.0E+07
-10 -20 -30 -40 -50 -60 -70 -80 -90 -100 -110 -120 -130 -140 -150 -160 -170 -180 -190
f1 (ppm)

4. LCMS traces

4.1. 4-(3-(Benzyloxy)phenyl)-2-(methylsulfinyl)-6-(trifluoromethyl)pyrimidine

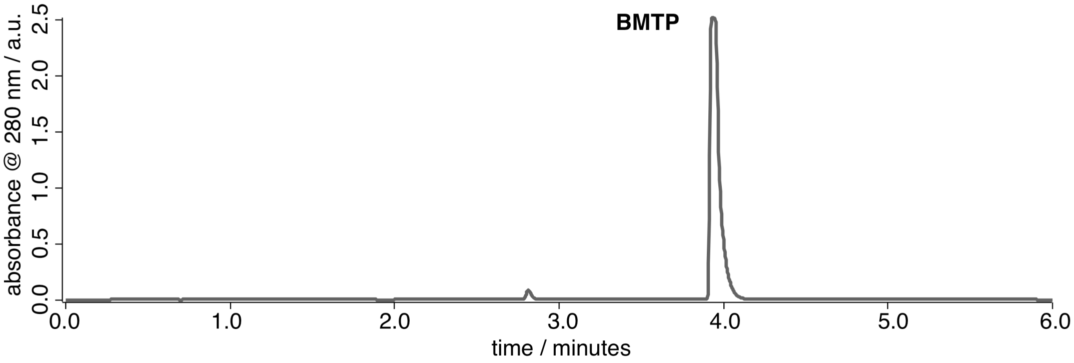

4.2. *cis*-2-(Methylsulfinyl)-4-(3-styrylphenyl)-6-(trifluoromethyl)pyrimidine

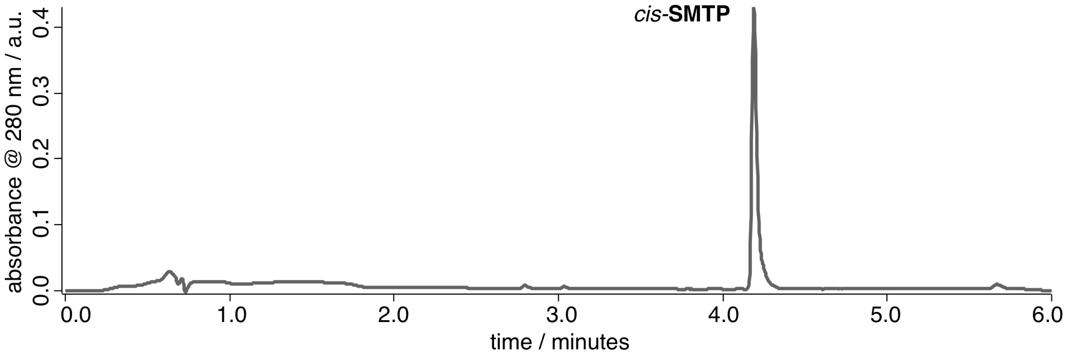

4.3. *trans*-2-(Methylsulfinyl)-4-(3-styrylphenyl)-6-(trifluoromethyl)pyrimidine

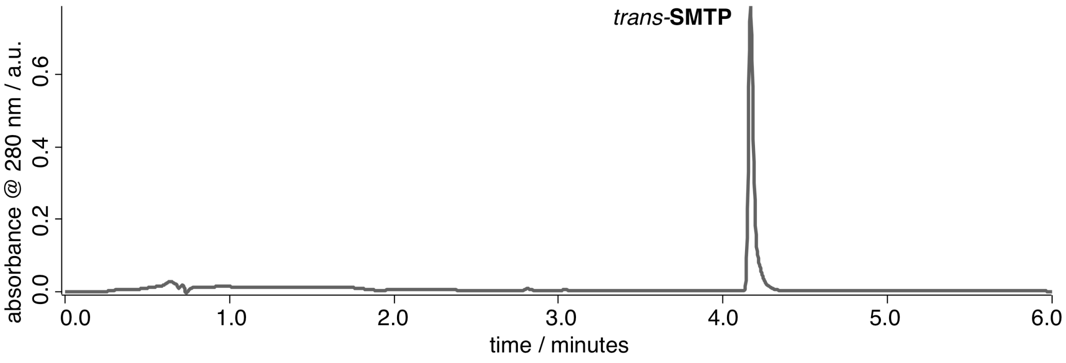

4.4. *cis*-2-(Ethylsulfinyl)-4-(3-styrylphenyl)-6-(trifluoromethyl)pyrimidine

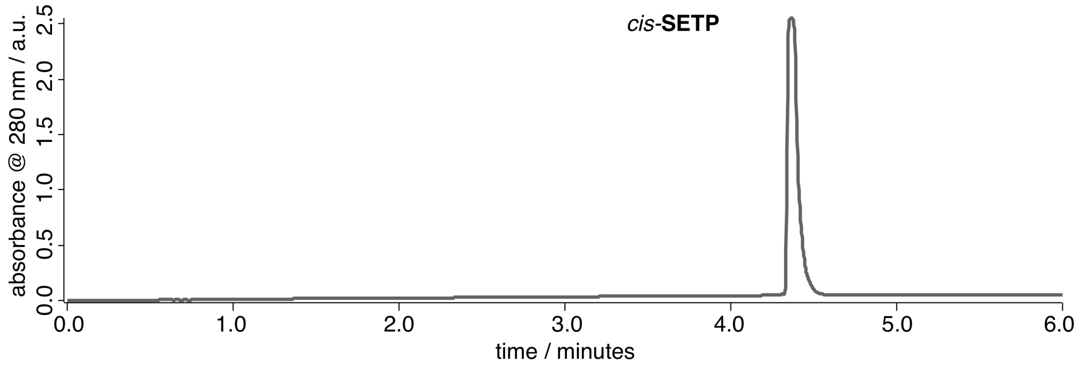

4.5. *trans*-2-(Ethylsulfinyl)-4-(3-styrylphenyl)-6-(trifluoromethyl)pyrimidine

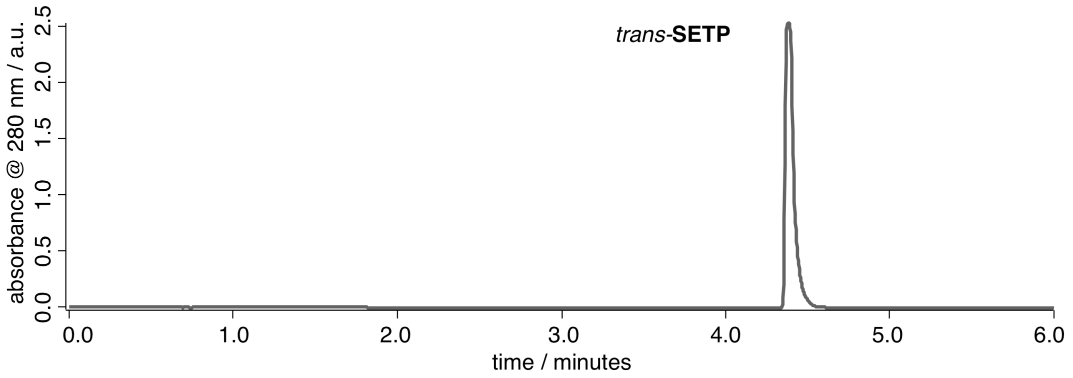

5. X-Ray crystallographic tables

5.1. Crystal data and structure refinement for *trans*-6

Identification code	*trans*-**6**	
Empirical formula	$C_{14}H_{11}Br$	
Formula weight	259.14	
Temperature	100(2) K	
Wavelength	0.71073 Å	
Crystal system	Monoclinic	
Space group	$P2_1$	
Unit cell dimensions	a = 5.6882(2) Å	a= 90°.
	b = 7.9539(3) Å	b= 98.274(4)°.
	c = 12.5338(5) Å	g = 90°.
Volume	561.17(4) Å^3	
Z	2	
Density (calculated)	1.534 Mg/m^3	
Absorption coefficient	3.624 mm^{-1}	
F(000)	260	
Crystal size	0.553 x 0.393 x 0.239 mm^3	
Theta range for data collection	3.62 to 32.79°.	
Index ranges	$-8 \leq h \leq 8$, $-11 \leq k \leq 11$, $-18 \leq l \leq 18$	
Reflections collected	12131	
Independent reflections	3821 [R(int) = 0.0409]	
Completeness to theta = 32.79°	95.0%	
Absorption correction	Semi-empirical from equivalents	
Max. and min. transmission	1.00000 and 0.34762	
Refinement method	Full-matrix least-squares on F^2	
Data / restraints / parameters	3821 / 1 / 137	
Goodness-of-fit on F^2	1.058	
Final R indices [I>2sigma(I)]	R1 = 0.0339, wR2 = 0.0612	
R indices (all data)	R1 = 0.0418, wR2 = 0.0646	
Absolute structure parameter	-0.005(9)	
Extinction coefficient	0.0066(15)	
Largest diff. peak and hole	0.524 and -0.566 e.Å^{-3}	
CCDC	1530566	

5.2. Crystal data and structure refinement for BETP.

Identification code	**BETP**	
Empirical formula	$C_{20}H_{17}F_3N_2O_2S$	
Formula weight	406.42	
Temperature	140.00(10) K	
Wavelength	1.54184 Å	
Crystal system	Monoclinic	
Space group	$P2_1/c$	
Unit cell dimensions	a = 8.4974(4) Å	a= 90°.
	b = 5.2063(2) Å	b= 91.181(5)°.
	c = 41.169(3) Å	g = 90°.
Volume	1820.94(18) $Å^3$	
Z	4	
Density (calculated)	1.482 Mg/m^3	
Absorption coefficient	2.027 mm^{-1}	
F(000)	840	
Crystal size	0.59 x 0.08 x 0.06 mm^3	
Theta range for data collection	3.22 to 75.36°.	
Index ranges	$-10 \leq h \leq 10$, $-6 \leq k \leq 4$, $-51 \leq l \leq 50$	
Reflections collected	12366	
Independent reflections	3814 [R(int) = 0.0458]	
Completeness to theta = 75.36°	98.3%	
Absorption correction	Gaussian	
Max. and min. transmission	0.977 and 0.869	
Refinement method	Full-matrix least-squares on F^2	
Data / restraints / parameters	3814 / 1 / 262	
Goodness-of-fit on F^2	1.184	
Final R indices [I>2sigma(I)]	R1 = 0.1108, wR2 = 0.2687	
R indices (all data)	R1 = 0.1149, wR2 = 0.2705	
Largest diff. peak and hole	0.673 and -0.541 e.$Å^{-3}$	
CCDC	1530565	

6. *Biology*

6.1. Peptides

GLP-1(7-36)NH2 and exendin(9-39) were purchased from Sigma. GLP-1(9-36)NH2 was purchased from Insight Biotechnology. GLP-1-FITC, i.e. GLP-1 7-36NH2 conjugated with fluorescein isothiocyanate at Lys28, was purchased from Insight Biotechnology. All peptides had a purity of at least 90%.

6.2. Cell culture

Pathhunter CHO-GLP-1R β-arrestin2 reporter cells (DiscoverX) were maintained in Culture Media 6 (DiscoverX) at 37°C, 5% CO_2 and used beween passages 1-10. Monoclonal CHO-SNAP-GLP-1R cells were prepared in house by transfecting wildtype CHO-K1 cells with the N-terminally SNAP-tagged human GLP-1R (pSNAP-GLP-1R, Cisbio), G418 selection, and flow cytometric sorting to single cell colonies. CHO-SNAP-GLP-1R cells were maintained in complete CHO medium (DMEM, 10% FBS, 1% penicillin/streptomycin, 5 mM glucose, 10 mM HEPES, 1% non-essential amino acids) supplemented with G418 500 µg/ml.

6.3. cAMP assays

Pathhunter CHO-GLP-1R β-arrestin2 reporter cells were seeded overnight in CHO medium at a density of 10,000 cells/well in 96-well white plates. GLP-1R PAMs or DMSO vehicle control from frozen DMSO aliquots were prepared in complete medium to guarantee full dissolution. Peptides were prepared fresh from lyophilised stocks. No phosphodiesterase inhibitors were used. After aspirating overnight medium, compounds were added at the indicated concentration and incubated for 30 minutes at 37°C. The assay was terminated by addition of lysis buffer from the measurement kit, and cAMP determined by HTRF (cAMP Dynamic 2, Cisbio) read on a i3x plate reader (Molecular Devices).

6.4. Ca^{2+} measurements

Pathhunter CHO-GLP-1R β-arrestin2 reporter cells were seeded overnight in CHO medium at a density of 50,000 cells/well in 96-well black plates. Medium was aspirated

and replaced with Calcium 6 dye (Molecular Devices) prepared in complete medium and supplemented with probenecid (2.5 mM). Cells were loaded for 90 minutes at 37°C. Baseline fluorescence (excitation 485 nm, emission 520 nm) was read using a Flexstation 3 instrument (Molecular Devices). GLP-1R PAMs, prepared as before, were added to wells, and fluorescence was serially monitored at 37°C, with or without robotic addition of GLP-1(7-36)NH2 (100 nM) at specific time points, depending on the assay. Where indicated, ionomycin (3 µM) was added to determine if dye saturation had been achieved, or ATP (10 µM) added to identify intracellular Ca^{2+} store depletion. For inhibitor studies, the protocol was adapted to reduce the amount of serum in the assay, as this is known to interfere with the action of the PLC inhibitor U73122;[4] therefore, cells were loaded with Calcium 6 dye prepared in HBSS + 10 mM HEPES, and GLP-1R PAMs were dissolved in HBSS + 1% FBS. Inhibitors (ESI-09 10 µM, U73122 5 µM or DMSO control) were prepared in HBSS and added 15 minutes before PAMs.

6.5. β-Arrestin assays

Pathhunter CHO-GLP-1R β-arrestin2 reporter cells were seeded overnight in CHO medium at a density of 10,000 cells/well in 96 well white plates. After aspirating overnight medium, compounds prepared in CP2 complete medium (DiscoverX) were added and incubated for 90 minutes at 37°C. Pathhunter detection reagents (DiscoverX) were added at the end of the incubation period and β-arrestin recruitment determined as luminescent signal read using an i3x plate reader.

6.6. Cell surface binding kinetics

Ligand-receptor interaction was monitored using an adaptation of a previously published technique which utilises time-resolved Förster resonance energy transfer (TR-FRET) between N-terminally SNAP-tagged GPCRs and fluroescently conjugated ligand.[5] Long-lived emission from the lanthanide probe Lumi4-Tb enables time-gated signal detection and avoids short-lived autofluoresence from cells and plasticware. This probe displays several emission peaks including one at 485 nm which makes fluorescein and derivatives suitable FRET acceptors. SNAP-CHO-GLP-1R cells were seeded overnight, 20,000 cells/well, in 96 well white plates, and labeled for 60 minutes with 40 nM Lumi4-Tb (a kind gift from Dr. Louise Affleck, Cisbio) in HBSS + 1% BSA for 1 hour at 37°C.

To prevent GLP-1R endocytosis, a cocktail of metabolic inhibitors[6] consisting of 2-deoxyglucose (20 mM) and NaN_3 (10 mM) was added 20 minutes before the end of labelling, and present during the assay thereafter. Cells were washed 3 times in HBSS and PAMs allowed to bind for 10 minutes prior to addition of GLP-1-FITC at several concentrations, with serial measurement of TR-FRET (excitation 350 nm, emissions 520 nm and 620 nm, delay 50 ms, integration time 400 ms) every minute for 120 minutes. Binding was calcuated at each timepoint as the ratio of signals at 520 nm (FITC) and 620 nm (Lumi4-Tb).

6.7. Endosomal binding studies

The TR-FRET binding protocol was further adapted to detect binding within endosomes. SNAP-CHO-GLP-1R cells were labeled as before but without endocytosis inhibition. Freshly prepared GLP-1-FITC (100 nM) was added for the last 30 minutes of the labelling period to induce complete GLP-1R internalization; for non-specfic binding 10 µM unlabelled exendin-4 was added 5 minutes in advance. Agonist was then removed by washing 3 times with HBSS, and replaced with PAMs and exendin(9-39) at 5 µM (to prevent possible rebinding events). The plate was then quickly moved to the plate reader and TR-FRET was monitored as before On the basis that this concentration of GLP-1-FITC induces virtually complete GLP-1R internalisation, followed by extensive washing and the presence of exendin(9-39), it is expected that the FRET results from internalised ligand-receptor complexes, which are accessed by the membrane-permeating GLP-1R PAMs.

6.8. Confocal microscopy

CHO-SNAP-GLP-1R cells were seeded onto glass coverslips and adhered overnight. Labeling of cell surface SNAP-GLP-1Rs with 1 mM SNAP-Surface-549 (New England Biolabs) was performed in complete DMEM for 60 minutes. After washing, cells were treated ± 100 nM GLP-1-FITC at 37°C for 30 minutes, prior to fixation with 2% PFA. Coverslips were mounted in Vectashield Hardset with 4,6-diamidino-2-phenylindole (Vector Laboratories) and images were acquired with a Zeiss LSM-780 inverted confocal laser-scanning microscope with a 63x/1.4 numerical aperture oil-immersion objective.

Images were subjected to a Gaussian smooth (1.30) and linear changes in brightness and contrast using ZenLite (Zeiss) and prepared in ImageJ (NIH).

6.9. Data analysis

All biological data analyses were performed in Graphpad Prism 6 (Graphpad Software). Where indicated, data were normalised to relevant baseline or maximal response. Dose-response data were fitted using a 4-parameter logistic fit to determine EC_{50} values and their negative logarithm, pEC_{50}. Additionally, when exendin(9-39) was used as the orthosteric probe in cAMP assays, agonist activity was determined as transduction ratios (τ/K_A) using previously described equations.[7] FITC-agonist binding data were globally fit by non-linear regression as previously described[8] to determine rate constants for association (k_{on}) and dissociation (k_{off}). Binding affinity (K_d) is k_{off} / k_{on}, and residence time is the reciprocal of k_{off}. Experiments were performed with a matched design and statistical analysis was performed using randomized block ANOVAs, with post hoc testing according to the primary question of the analysis – for example, when specifically comparing *cis- vs. trans-* isoforms, Sidak's test was used. Other post hoc tests are indicated in the figure legends.

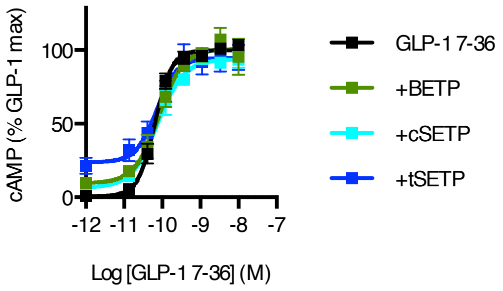

Figure S1. Cyclic AMP responses to GLP-1(7-36)NH_2 (GLP-1 7-36) in PathHunter CHO-GLP-1R cells in the presence of 10 μM PAM or CL-PAM (30 min incubation, $n = 3$). Data indicated as mean ± SEM.

	pEC_{50} ± SEM		Log (τ/K_A) ± SEM
	GLP-1 7-36	GLP-1 9-36	Exendin(9-39)
vehicle	10.2 ± 0.1	6.7 ± 0.1	N.C.
+BETP	10.1 ± 0.1	7.6 ± 0.0	7.9 ± 0.0
+cSETP	10.1 ± 0.1	7.5 ± 0.1	7.8 ± 0.2
+tSETP	10.2 ± 0.1	7.9 ± 0.1 *	8.1 ± 0.1 *
+BMTP	10.1 ± 0.1	7.6 ± 0.1	8.0 ± 0.1
+cSMTP	10.2± 0.1	7.3 ± 0.1	7.9 ± 0.1
+tSMTP	10.2 ± 0.0	7.8 ± 0.0 #	8.3 ± 0.0 #

Table S1. Potency estimates (pEC_{50}, *i.e.* negative logarithm of EC_{50}) or log transduction ratios (log (τ/K_A)) for cAMP responses to GLP-1(7-36)NH_2, GLP-1(9-36)NH_2 and exendin(9-39) ± allosteric enhancement by 10 µM indicated PAM. Transduction ratios used for exendin(9-39) due to variable E_{max}. Estimates derived from data in Figures 2a,b and Supplementary Figure 1. * $p<0.05$, *cis*-**SETP** *vs. trans*-***S*ETP**; # $p<0.05$, *cis*-**SMTP** *vs. trans*-**SMTP**.

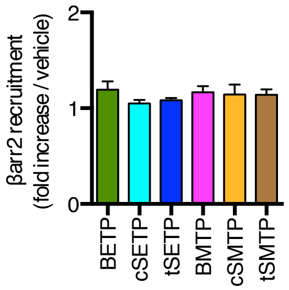

Figure S2. 10 μM PAM-induced β-Arrestin2 (βarr2) recruitment (90 min incubation, *n* = 3), without orthosteric ligand. Expressed as fold increase *vs*. vehicle alone. Values represent the mean + SEM.

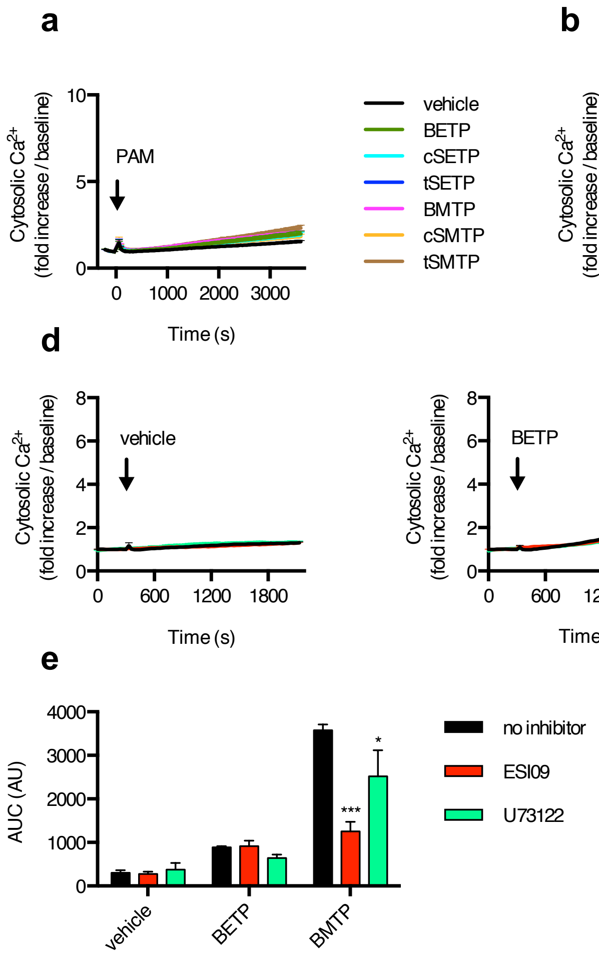

Figure S3. Investigation of PAM-induced Ca^{2+} responses. a) 10 μM PAM-induced Ca^{2+} response in wildtype CHO-K1 cells (without GLP-1R overexpression) (*n* = 3). **b**) Inhibition of GLP-1(7-36)NH_2 (GLP-1)–induced Ca^{2+} response in PathHunter CHO-GLP-1R cells. Cells pretreated with ESI09 (10 μM) or U73122 (5 μM) before addition of 100 nM GLP-1 (*n* = 3). **c**) AUC from b) determined relative to normalized baseline. **d**) Vehicle / **BETP** / **BMTP** Ca^{2+} responses after pretreatment with inhibitor as above (*n* = 3) **e)** As for d), but AUC quantification relative to normalized baseline.. *P<0.05, ***P<0.0001; one-way or two-way randomized block ANOVA followed by Dunnett's test *vs.* no inhibitor response. Values represent the mean + SEM.

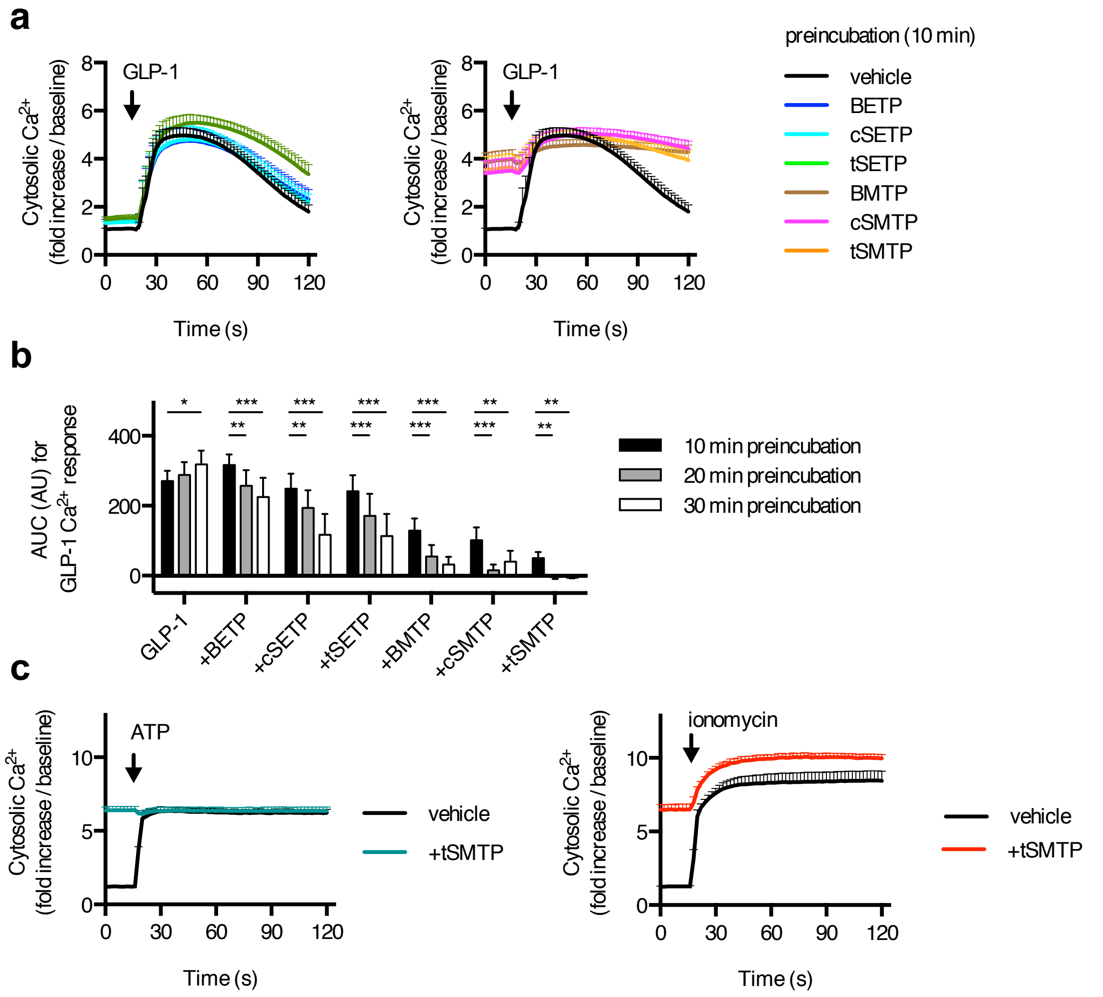

Figure S4. Allosteric modulation of GLP-1-induced Ca^{2+} responses. a) Allosteric modulation of 100 nM GLP-1 (7-36)NH_2 Ca^{2+} response following 10 min pre-incubation with PAM or CL-PAM (10 µM), trace expressed relative to baseline recorded before PAM addition (*n* = 5). **b**) Effect of 10 µM PAM preincubation duration on subsequent GLP-1(7-36)NH_2 (100 nM) –induced Ca^{2+} rise, indicated as AUC relative to progressively elevated baseline with ongoing PAM exposure (*n* = 3), two-way randomized block ANOVA followed by Dunnett's test *vs.* 10 min preincubation response. (**c**) Response to ATP (10 µM – positive control for intracellular Ca^{2+} release) or ionomycin (3 µM – positive control to exclude Ca^{2+} dye saturation as induces Ca^{2+} influx

from extracellular buffer) after 30 min precincubation with tSMTP or vehicle (*n* = 3). Values represent the mean + SEM.

7. *References*

[1] Agilent Technologies, *Crysalis PRO*, 1.171.138.143.

[2] G. Sheldrick, *Acta Crystallographica Section A* **2008**, *64*, 112-122.

[3] T. Ishiyama, M. Murata, N. Miyaura, *Journal of Organic Chemistry* **1995**, *60*, 7508-7510.

[4] N. E. Wilsher, W. J. Court, R. Ruddle, Y. M. Newbatt, W. Aherne, P. W. Sheldrake, N. P. Jones, M. Katan, S. A. Eccles, F. I. Raynaud, *Drug Metab. Dispos.* **2007**, *35*, 1017-1022.

[5] A. Emami-Nemini, T. Roux, M. Leblay, E. Bourrier, L. Lamarque, E. Trinquet, M. J. Lohse, *Nat. Protoc.* **2013**, *8*, 1307-1320.

[6] C. Widmann, W. Dolci, B. Thorens, *Biochem. J.* **1995**, *310 (Pt 1)*, 203-214.

[7] a) T. Kenakin, C. Watson, V. Muniz-Medina, A. Christopoulos, S. Novick, *ACS Chemical Neuroscience* **2012**, *3*, 193-203; b) E. T. van der Westhuizen, B. Breton, A. Christopoulos, M. Bouvier, *Mol. Pharmacol.* **2014**, *85*, 492-509.

[8] C. Klein Herenbrink, D. A. Sykes, P. Donthamsetti, M. Canals, T. Coudrat, J. Shonberg, P. J. Scammells, B. Capuano, P. M. Sexton, S. J. Charlton, et al., *Nat. Commun.* **2016**, *7*, 10842.
